# Genome-wide DNA methylation analysis in blood cells from patients with Werner syndrome

**DOI:** 10.1186/s13148-017-0389-4

**Published:** 2017-08-30

**Authors:** T. Guastafierro, M. G. Bacalini, A. Marcoccia, D. Gentilini, S. Pisoni, A. M. Di Blasio, A. Corsi, C. Franceschi, D. Raimondo, A. Spanò, P. Garagnani, F. Bondanini

**Affiliations:** 1 UOC of Clinical Biochemistry, Sandro Pertini Hospital, Rome, Italy; 2 CRIIS (Interdisciplinary, Interdepartmental and Specialistic Reference Center for Early Diagnosis of Scleroderma, Treatment of Sclerodermic Ulcers and Videocapillaroscopy), Sandro Pertini Hospital, Rome, Italy; 3 IRCCS Institute of Neurological Sciences, Bologna, Italy; 4 UOSD Ischemic Microangiopathy and Sclerodermic Ulcers, Sandro Pertini Hospital, Rome, Italy; 5 Centre for Biomedical Research and Technologies, Italian Auxologic Institute, IRCCS, Milan, Italy; 6 Department of Molecular Medicine, Sapienza University of Rome, Rome, Italy; 7 Department of Experimental, Diagnostic and Specialty Medicine, University of Bologna, Bologna, Italy; 8 Interdepartmental Center “L. Galvani”, University of Bologna, Bologna, Italy; 9 Center for Applied Biomedical Research (CRBA), St. Orsola-Malpighi University Hospital, Bologna, Italy; 10 Clinical Chemistry, Department of Laboratory Medicine, Karolinska Institute at Huddinge University Hospital, S-141 86 Stockholm, Sweden; 11 CNR Institute for Molecular Genetics, Unit of Bologna, Bologna, Italy; 12 Laboratory of Musculoskeletal Cell Biology, Rizzoli Orthopedic Institute, Bologna, Italy; 13 UOC of Clinical Pathology, Saint’ Eugenio Hospital, Rome, Italy

**Keywords:** Werner syndrome, DNA methylation, Systemic sclerosis

## Abstract

**Background:**

Werner syndrome is a progeroid disorder characterized by premature age-related phenotypes. Although it is well established that autosomal recessive mutations in the WRN gene is responsible for Werner syndrome, the molecular alterations that lead to disease phenotype remain still unidentified.

**Results:**

To address whether epigenetic changes can be associated with Werner syndrome phenotype, we analysed genome-wide DNA methylation profile using the Infinium MethylationEPIC BeadChip in the whole blood from three patients affected by Werner syndrome compared with three age- and sex-matched healthy controls. Hypermethylated probes were enriched in glycosphingolipid biosynthesis, FoxO signalling and insulin signalling pathways, while hypomethylated probes were enriched in PI3K-Akt signalling and focal adhesion pathways. Twenty-two out of 47 of the differentially methylated genes belonging to the enriched pathways resulted differentially expressed in a publicly available dataset on Werner syndrome fibroblasts. Interestingly, differentially methylated regions identified *CERS1* and *CERS3*, two members of the ceramide synthase family. Moreover, we found differentially methylated probes within *ITGA9* and *ADAM12* genes, whose methylation is altered in systemic sclerosis, and within the *PRDM8* gene, whose methylation is affected in dyskeratosis congenita and Down syndrome.

**Conclusions:**

DNA methylation changes in the peripheral blood from Werner syndrome patients provide new insight in the pathogenesis of the disease, highlighting in some cases a functional correlation of gene expression and methylation status.

**Electronic supplementary material:**

The online version of this article (doi:10.1186/s13148-017-0389-4) contains supplementary material, which is available to authorized users.

## Introduction

Werner syndrome (WS) is a rare adult premature ageing disease. Individuals affected by WS generally have a normal development until the third decade of life, when premature ageing phenotypes and symptoms begin to manifest including premature greying or loss of hair, bird-like faces, cataracts, sclerodermiform skin atrophy [1] and skin ulcers [2]. Because of apparent similar skin changes, WS is often misdiagnosed as systemic sclerosis (SSc) [3].

Particularly, WS represents an important part of the differential diagnosis in patients who present with scleroderma-like skin changes, as they share with SSc patients the main histological changes of skin. These include replacement of the subcutaneous tissue by hyalinized connective, hyalinized connective tissue in the lower dermis and hyalinization and formation of aneurysms in the dermal blood vessels [4, 5]. Otherwise, progeria, premature cataract, type 2 diabetes mellitus, sensorineural hearing loss, premature atherosclerosis and dyslipidemia in WS besides the absence of anti-scleroderma antibodies may be helpful for the differential diagnosis of WS [6]. WS is associated to an autosomal recessive mutation of Werner syndrome gene (*WRN*) [7, 8]; up today, more than 50 different disease-causing mutations in the *WRN* gene have been identified [8]. The *WRN* gene encodes a 180-kDa nuclear protein member of the RecQ subfamily of DNA and RNA helicases [8, 9] with an intrinsic 3′ to 5′ DNA helicase activity [10]. DNA helicases are involved in many aspects of DNA metabolism including transcription, replication and recombination [9]. Given its role as a 3′ to 5′ exonuclease and based on interactions between this protein and Ku70/80 heterodimer in DNA end processing, WRN plays a critical role in the repair of DNA double-strand breaks [11]. Additionally, recent studies suggest a role for WRN in maintaining DNA telomere stability [10]. It is well known that defects in telomere structure and/or function may have a strong impact on human health, leading to premature ageing and a variety of diseases [12, 13]. Overall, WRN protein is crucial in maintaining genome structure and integrity, and accordingly, WS patients exhibit early age-associated biomarkers like DNA damage accumulation and chromosomal instability [14]. Despite these evidences, the molecular bases of WS phenotype are still largely unknown.

Alterations in epigenetic patterns, in particular in DNA methylation profiles, could contribute to WS phenotype. DNA methylation, which consists in the addition of a methyl group to the cytosine in a CpG dinucleotide, is a key mechanism in development and differentiation, and profound changes in DNA methylation patterns occur during ageing and age-related diseases [15, 16]. At present, few studies assessed DNA methylation changes in WS. Heyn and colleagues evaluated genome-wide DNA methylation in lymphoblastoid cell lines (LCLs) derived from Epstein-Barr virus (EBV)-immortalized B cells of four WS patients and three healthy controls [7], observing profound DNA methylation changes in WS. More recently, a WRN promoter was found hypermethylated in naïve B lymphocytes and lymphoblastoid cell lines from two brothers carrying a novel homozygous WRN mutation. Finally, Maierhofer et al. applied Horvath’s epigenetic clock to a dataset including the whole blood from WS patients and age-matched controls, demonstrating that the disease is associated with an increase in epigenetic age [17].

In the present study, we analysed genome-wide DNA methylation in the peripheral whole blood from three WS patients and their age- and sex-matched controls using the Infinium MethylationEPIC BeadChip. Our results indicate that specific genome-wide alterations occur in WS, which partially overlap those that occur in diffuse and limited SSc [18].

## Methods

### Samples

**Fig. 1:**
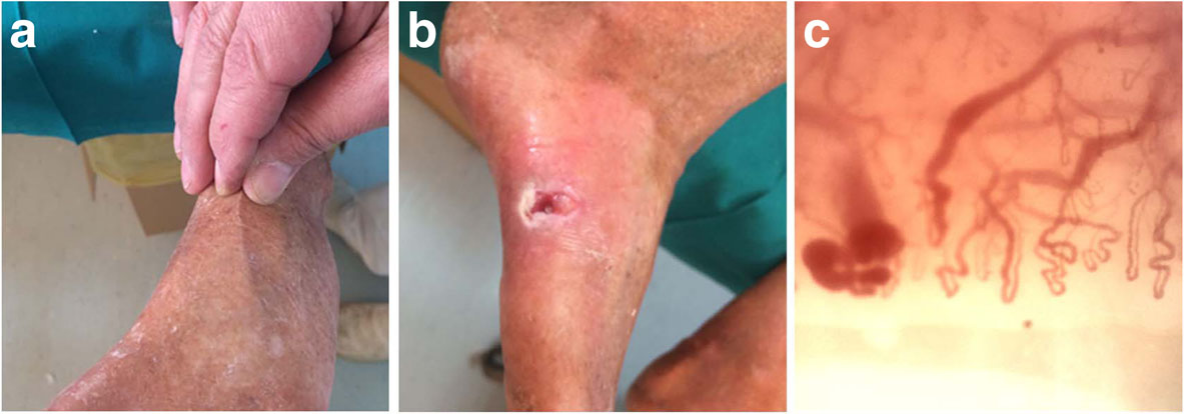
SSc typical skin signs in WS patients. **a** Skin unable to be lifted in pliers and/or pinched. **b** Cutaneous ulcer with calcinosis. **c** Capillaroscopic alterations and ectasias

Subjects were enrolled in the study “Early diagnosis of scleroderma and identification of predictor factors of disease development”. The study was approved by the Ethic Committee of ASL RM/B, and all participants signed the informed consent forms. WS was diagnosed according to clinical appearance and genetic testing. Two patients were siblings and carried the mutation c.2630+1G>A in the *WRN* coding sequence; the third patient carried the homozygous g.nt 77177 a>g mutation (GenBank acc. no. AY44237) in the *WRN* gene. They suffer of skin ulcers of suspected SSc derivation as they showed typical SSc skin signs being bound tightly to the underlying tissues and around joints causing an inability to pinch or lift the skin up (Fig. 1a) and calcinosis cutis, a type of calcinosis which characterized scleroderma skin ulcers [19–21] (Fig. 1b). Serum was collected, and anti-nuclear antibodies (ANAs) were analysed using indirect immunofluorescence (IIF) assays on ANA-HEp-2 cells (A. Menarini Diagnostics) according to the manufacturer’s protocol. Anti-extractable nuclear antigen (ENA) antibodies [anti-Sjögren’s syndrome A (SS-A), anti-Sjögren’s syndrome B (SS-B), anti-Smith (SM), anti-ribonucleoprotein (RNP), anti-topoisomerase I (SCL-70) and anti-histidyl-tRNA synthetase (JO-1)] were also dosed using chemiluminescence assay (Zenit RA autoimmunity, A. Menarini Diagnostics) according to the manufacturer’s protocol. These autoantibodies, considered as SSc diagnostic markers, were undetectable in selected patients (data not shown). SSc patients were also subjected to nailfold video capillaroscopy (NVC). Capillaroscopic alterations and ectasias compatible with diagnosis of acrocyanosis were detected in all three patients (Fig. 1c). Peripheral blood was collected from the three patients.

### Genome-wide DNA methylation analysis

Genomic DNA was extracted from whole blood using the Duplica^®^ Blood DNA Kit (EuroClone) on Duplica^®^ Prep Platform (EuroClone). DNA was bisulfite-converted using the EZ DNA Methylation-Gold Kit (Zymo Research) and analysed using the Infinium HumanMethylationEPIC BeadChip (Illumina) following the manufacturer’s instructions. Signal intensity files were extracted using the *minfi* Bioconductor package. Normalization was performed using the functional normalization function implemented in *minfi*. DNA methylation data are available at the NCBI Gene Expression Omnibus (GEO) database (http://www.ncbi.nlm.nih.gov/geo/) under accession number GSE100825.

DNA methylation differences between WS patients and healthy controls were assessed using two different approaches: (1) we used analysis of variance (ANOVA) to find out whether there was any statistically significant difference between WS patients and healthy controls when we consider differentially methylated positions (DMPs), that is single-CpG sites whose methylation differs between the groups under investigation. DMPs with a non-adjusted *P* value < 0.001 were retained as significantly differentially methylated. (2) We applied multivariate analysis of variance (MANOVA) on sliding windows including three adjacent CpG sites as described in Bacalini et al. [15] in order to identify statistically significantly differentially methylated regions (DMRs), that is regions in which multiple adjacent CpG sites differ between the two groups under investigation. This approach was applied only to the CpG probes mapping in CpG-rich regions (CpG islands, shores and shelves) associated to a gene. DMRs with a non-adjusted *P* value < 0.001 and in which at least two adjacent CpG sites had a minimum absolute DNA methylation difference of 0.3 were retained as significant. Although a 0.15 threshold for mean DNA methylation difference is often suggested [22], here we preferred to use a more stringent criterion, given the small sample size, in order to provide a short, but more likely reproducible, list of DMRs.

Infinium450k datasets GSE42865 and GSE75310 were downloaded from the GEO database. GSE42865 includes DNA methylation data from the LCL of three Hutchinson-Gilford progeria syndrome patients, four WS patients and three healthy patients, together with peripheral blood mononuclear cells from three healthy subjects and naïve B cells from three healthy subjects. We compared the four WS patients and the three LCL controls by ANOVA. GSE75310 includes DNA methylation data from four dyskeratosis congenita (DKC) patients. To investigate DNA methylation differences in DKC, we performed ANOVA using as controls the same four GEO samples (GSM796678, GSM796674, GSM796667 and GSM796671) used in the original paper [23].

### Pathway enrichment

Pathway analysis of differentially methylated genes and gene clusters was performed with the publicly available tool Enrichr (http://amp.pharm.mssm.edu/Enrichr) that provides access to various gene set libraries [24, 25]. Enrichr currently contains annotated gene sets from 102 gene set libraries organized in eight categories. Details of the gene set libraries in Enrichr can be found in previously published studies [24, 25]. Enrichment analysis checks whether the input set of genes significantly overlaps with annotated gene sets. Each gene set within the Enrichr database is associated with a functional term or an enrichment term such as a pathway, cell line or disease. The output of Enrichr is ranked lists of terms, one list for each gene set library. The most highly ranked enrichment terms for the user’s input gene list provide knowledge about the input list. We used the publicly Kyoto Encyclopedia of Genes and Genomes (KEGG) pathway gene sets to identify the relatively enriched biological pathways and then extracted the enriched gene sets for each pathway. We considered pathways as enriched if their *P* value was lower than 0.05 and ranked them using the combined score (CS). CS is a multiplication of the *P* value computed using Fisher’s exact test with the z-score of the deviation from the expected rank. In particular, *c* is equal to log (*p*) × *z* where *c* is the combined score, *p* is the *P* value computed using Fisher’s exact test and *z* is the z-score computed to assess the deviation from the expected rank.

### Differential gene expression analysis

In order to perform differential expression analysis, we used shinyGEO (http://gdancik.github.io/shinyGEO/) [26], a Web-based tool that allows a user to download the expression and sample data from a GEO dataset, select a gene of interest and perform a survival or differential expression analysis using the available data [27]. For both analyses, shinyGEO is able to generate R code, ensuring that all analyses are reproducible. The tool is developed using shiny, a Web-based application framework for R, a language for statistical computing and graphics. Fold change (FC) and *P* value are calculated by two-sample Student’s *t* test for differential expression.

## Results

We used the Infinium MethylationEPIC BeadChip to compare whole blood genome-wide DNA methylation profiles between three WS patients and three age- and sex-matched controls (CTRs). We conducted principal component analysis to identify major traits of variation between samples (Additional file 1: Figure S1). No clear separation between WS patients and CTRs was observed along the first component (which explained 32% of the variance) or the second component (which explained 51% of the remaining variance).

**Fig. 2:**
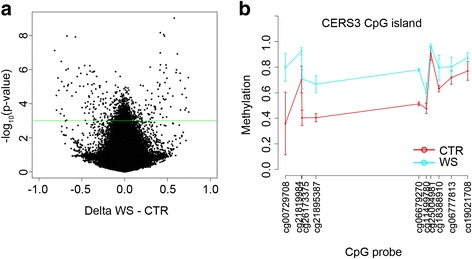
DNA methylation alterations in Werner syndrome. **a** Volcano plot of DMPs between WS patients and CTRs. The difference between mean DNA methylation values in WS patients and in CTRs is plotted on the *x*-axis, while the non-adjusted *P* value for ANOVA between the two groups is on the *y*-axis (− 1 × log10 scale). The green line corresponds to a non-adjusted *P* value of 0.001. **b** DNA methylation profile of the CpG island located in the *CERS3* gene

ANOVA identified 1125 DMPs that distinguished WS patients from CTRs (non-adjusted *P* value < 0.001) (Fig. 2a, Additional file 2, Table 1). Of these, 511 probes (mapping in 382 genic regions) were hypermethylated and 614 probes (mapping in 416 genic regions) were hypomethylated in WS patients compared to CTRs. Several DMPs showed large DNA methylation differences between the two groups, with 87/511 hypermethylated and 110/614 hypomethylated probes having mean methylation differences larger than 0.15 [22]. The volcano plot in Fig. 2a shows many CpG sites with non-adjusted *P* values lower than 0.001 but having low DNA methylation differences between the groups. This behaviour might be related to the small number of analysed WS samples.

**Table 1:** List of the top 20 DMPs

Probe	CHR	MAPINFO	Gene	CpG island name	Relation with respect to the CpG island	Non-adjusted *P* value
cg15294279	3	174,842,010	NAALADL2	–	–	9.48*E*−10
cg26845082	3	13,555,664	–	–	–	6.75*E*−09
cg23432430	12	125,538,377	–	chr12:125534060-125534527	S_Shelf	8.75*E*−09
cg00597723	5	158,691,793	UBLCP1	chr5:158690013-158690541	S_Shore	1.05*E*−08
cg13956086	7	2,434,521	–	–	–	1.08*E*−08
cg16995742	2	237,992,612	COPS8	chr2:237994004-237994876	N_Shore	1.19*E*−08
cg17779733	22	49,589,242	–	–	–	7.55*E*−08
cg06052372	16	83,967,808	–	–	–	1.04*E*−07
cg23928292	12	21,815,474	–	–	–	1.19*E*−07
cg10360725	8	144,139,316	–	–	–	3.31*E*−07
cg14782559	6	33,131,893	COL11A2	chr6:33129291-33129718	S_Shelf	5.19*E*−07
cg18673341	7	22,481,962	STEAP1B	–	–	5.28*E*−07
cg26822175	22	27,018,010	CRYBA4	–	–	5.71*E*−07
cg08161337	22	45,814,116	RIBC2	–	–	5.86*E*−07
cg22664298	5	128,795,827	ADAMTS19	chr5:128795503-128797417	Island	7.16*E*−07
cg13885829	1	17,482,041	–	–	–	7.98*E*−07
cg07584620	1	2,265,881	MORN1	chr1:2266007-2266432	N_Shore	8.00*E*−07
cg20757478	6	31,012,262	–	–	–	8.99*E*−07
cg17628377	5	180,121,337	–	–	–	1.03*E*−06
cg15865722	11	68,860,657	–	–	–	1.03*E*−06

Next, we performed a gene set enrichment analysis in order to examine in silico the biological functions of DMPs in WS patients compared to CTR samples. To this aim, we searched for the overrepresented biological pathways associated with the differentially methylated genes, using as input for the Enrichr tool the list of genes associated with DMPs. Enrichr’s combined score, a combination of the *P* value and z-score, was considered in order to prioritize enriched pathways (see “Methods” for more details). As detailed in the Enrichr manuscript [24], the combined score provides a compromise between both methods (*P* value and z-score) and, in several benchmarks, has reported the best ranking when compared with the other scoring schemes.

We took into account the most highly ranked enriched pathways according to the combined score while we considered significantly overrepresented pathways, those showing a *P* value < 0.05. Results for enriched biological pathways are shown in Table 2. The hypermethylated genes in KEGG pathway enrichment analysis results were associated with the enriched pathways with *P* value < 0.05, including “glycosphingolipid biosynthesis (HSA-00603)”, “FoxO signalling pathway (HSA-04068)” and “insulin signalling pathway (HSA-04910)”. The hypomethylated genes were associated with the enriched pathways with *P* value < 0.05, including “PI3K-Akt signalling (HSA-04151)” and “focal adhesion (HSA-04510)”.

**Table 2:** Gene set enrichment analysis details are reported for DPMs (hypo- and hypermethylated genes) and DMRs

	Enriched pathways	Pathway ID	Overlap	*P* value	Combined score	Overlapping genes
Hypermethylated probes	Glycosphingolipid biosynthesis	HSA-00603	3/14	0.001	2.29	ST3GAL1, GBGT1, ST3GAL2
	FoxO signal pathway	HSA-04068	8/133	0.003	2.60	MAPK10, USP7, AKT2, STAT3, PTEN, FOXO3, SKP2, GABARAP
	Insulin signalling pathway	HSA-04910	8/139	0.004	2.49	MAPK10, PTPN1, SHC2, AKT2, PRKAK1B, FASN, TSC2, CRKL
Hypomethylated probes	PI3K-Akt signalling	HSA_04151	16/341	0.006	3.46	CSF-1R, CDKN1B, TNXB, VWF, LAMA1, FLT4, THBS1, PTK2, LPAR5, PPP2R2B, PPP2R2D, MAPK1, COL6A6, FGFR1, BCL2L1, ITGA9
	Focal adhesion	HSA_04510	12/202	0.01	3.26	MAPK10, TNXB, VWF, ROCK2, LAMA1, FLT4, PXN, MAPK1, COL6A6, THBS1, PTK2, ITGA9
DMRs	Sphingolipid metabolism	HSA_00600	2/120	0.003	4.54	CERS3, CERS1
	Sphingolipid signalling pathway	HSA_047071	1/47	0.001	5.36	CERS3

Overlap indicates the number of hits from the differentially methylated gene sets compared to the KEGG gene set library, while “overlapping genes” column contains names of these hits. Differentially expressed genes between normal and WS fibroblasts in Cheung HH dataset [30] are reported. Enriched pathways were selected based on the *P* value. Combined score is a multiplication of the *P* value computed using Fisher’s exact test with the z-score of the deviation from the expected rank (see “Methods” for details)

Finally, we also used the pipeline reported by Bacalini et al. [15] (see “Methods” for details) to identify differentially methylated regions (DMRs) between WS patients and CTRs. We found 27 DMRs with a non-adjusted *P* value < 0.001 (Additional file 3, Additional file 4, Table 3), 20 of which were clearly hypermethylated in WS patients compared to CTRs. Even these 27 DMRs (corresponding to 37 genes) were employed in the Enrichr pathway analysis approach, revealing “sphingolipid metabolism (HSA-00600)” and “sphingolipid signalling pathway (HSA-04071)” among the most enriched pathways with *P* value < 0.05. Both pathways contain *CERS3* and *CERS1* genes, which are two members of the ceramide synthase family.

**Table 3:** List of the 27 DMRs

CHR	CpG island name	Relation with respect to the CpG island	Gene	Non-adjusted *P* value
18	chr18:30349690-30352302	S_Shore	KLHL14	1.70*E*−05
12	chr12:50297580-50297988	S_Shore	FAIM2	0.00013152
6	chr6:33396050-33396296	S_Shelf	SYNGAP1	0.000168391
5	chr5:110074605-110075223	N_Shore	SLC25A46	0.000215085
22	chr22:46366726-46368726	S_Shore	WNT7B	0.000233192
17	chr17:46620367-46621373	S_Shore	HOXB2; HOXB-AS1	0.000244681
10	chr10:5930914-5932389	N_Shore	ANKRD16; FBXO18	0.000261161
3	chr3:9811466-9811736	S_Shore	CAMK1	0.000281325
2	chr2:129075197-129077639	Island	HS6ST1	0.000292046
15	chr15:71145995-71146820	N_Shore	LARP6; LRRC49	0.000302033
15	chr15:101084428-101085178	Island	CERS3	0.000341282
6	chr6:143999154-143999667	N_Shore	PHACTR2	0.000393635
6	chr6:166755812-166756510	S_Shore	LOC100289495; SFT2D1	0.000423645
4	chr4:146540053-146540656	N_Shore	MMAA	0.000441415
17	chr17:20059028-20060060	N_Shore	SPECC1	0.000444431
6	chr6:31939730-31940559	S_Shore	DXO; STK19	0.000488824
15	chr15:99791328-99792042	N_Shore	TTC23; LRRC28	0.0004949
19	chr19:19006031-19007546	S_Shore	GDF1; CERS1	0.000509929
22	chr22:20134462-20134705	S_Shelf	LOC388849	0.000585452
19	chr19:19624954-19627258	Island	NDUFA13; TSSK6	0.000650774
1	chr1:160990718-160991225	N_Shore	F11R	0.000652612
6	chr6:33266302-33267582	N_Shore	RGL2	0.000759464
8	chr8:117886284-117887319	S_Shore	RAD21-AS1; RAD21; MIR3610	0.000773036
19	chr19:35491151-35492020	N_Shore	GRAMD1A	0.000798561
17	chr17:4692249-4693977	N_Shore	GLTPD2	0.000853965
4	chr4:9783035-9784960	Island	DRD5	0.000911924
4	chr4:1396291-1401730	Island	NKX1-1	0.000928038

To assess if the epigenetic pattern that we described in our WS cohort was reproducible in other WS DNA methylation datasets, we compared our results with those available from a publicly available dataset (GEO accession ID: GSE42865). GSE42865 includes DNA methylation data from LCL from four WS patients and three CTRs, measured using the previous version of the microarray, the Infinium450k BeadChip [7]. Of the 1125 DMPs identified in our analysis, 581 are included in the Infinium450k design. Of these, only two probes were differentially methylated between LCLs of WS patients and CTRs with a comparable statistical threshold (non-adjusted *P* value < 0.001), while 30 probes were found differentially methylated, considering a less stringent threshold (non-adjusted *P* value < 0.05) (Additional file 2). To investigate the reason of this low reproducibility of the data, we focused on the DMPs mapping in the genes resulting from the pathway enrichment analysis that are present in both the microarray versions. We included in this analysis also the probes mapping in *CERS1* and *CERS3* DMRs. As shown in Additional file 5, DNA methylation in LCL showed a larger variation in WS and/or CTR subjects, possibly as a consequence of the immortalization process [28]. Accordingly, DNA methylation differences were observed between LCL and naïve B cells (Additional file 5). It is therefore plausible that low overlapping between the GSE42865 dataset and ours is related to the different sources of genomic DNA (LCL vs whole blood). Despite these differences, we noted that several CpG sites showed the same trend in DNA methylation changes between WS patients and CTRs, in particular within the *CERS3* DMR and in *VWF* and *FOXO3* genes.

Furthermore, we compared WS-related epigenetic changes with those occurring in DKC, a premature ageing disease associated to impaired telomere maintenance. We exploited a publicly available Infinium450k dataset (GEO accession ID: GSE75310) performed on the whole blood from four DKC subjects [23]. Of the 581 WS-DMPs included in the Infinium450k design, 14 were differentially methylated in DKC with a comparable threshold (non-adjusted *P* value < 0.001), but only three of them showed a concordant DNA methylation change in the two diseases (Additional file 2). Despite this, we noted that the list of WS-DMPs included three CpG sites (cg27111250, cg10129063 and cg27639662) mapping in the S_Shore of the chr4:81109887-81110460 CpG island in the PR domain containing eight (*PRDM8*) genes and hypermethylated in WS patients compared to CTRs. This is of particular interest, as *PRDM8* is hypermethylated in DKC, aplastic anaemia (AA) [23] and Down syndrome [29], although in a different region (spanning from the CpG island chr4:81118137-81118603 to the CpG island chr4:81119095-81119391).

**Fig. 3:**
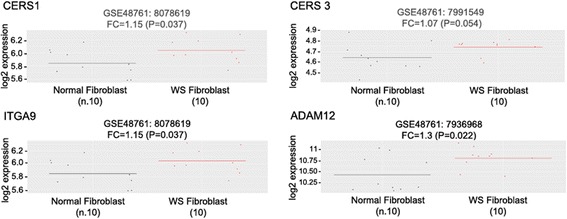
Differential expression of *CERS1*, *CERS3*, *ITGA9* and *ADAM12* genes based on the publicly available dataset on WS fibroblast analyses

Unfortunately, RNA was not available from the samples included in our cohort and it was not possible to assess the expression of the genes identified as differentially methylated. To overcome this limitation, we exploited the GEO database in order to get more insight into the relationship between DNA methylation changes and WS features. We selected the genes emerged from the pathway enrichment analysis, and we checked their expression in a dataset including ten WS and ten CTR skin fibroblasts (GEO accession ID: GSE48761) [30]. Enrichment analysis checks whether an input set of genes significantly overlaps with annotated gene sets. We found 47 genes overlapping with the annotated gene set from KEGG pathways (see the overlap column in Table 1), and 22 out of 47 of these genes were differentially expressed between WR patients and CTRs (*P* value < 0.05), as reported in Fig. 3 and Additional file 6: Figure S2. The relationship between DNA methylation and expression changes is reported in Additional file 7. Focusing on the DMRs, *HS6ST1* was hypermethylated in the whole blood from WS patients and downregulated in WS fibroblasts, while both *CERS1* and *CERS3* were hypermethylated in the whole blood from WS patients and upregulated in WS fibroblasts. Interestingly, we also noted that *PRDM8* gene expression is downregulated in WS fibroblasts (data not shown), like it was previously reported in the whole blood from DKC patients [30]. Although further investigation is needed, possibly on the same biological specimen from the same subject, these data underline an association between deregulation of DNA methylation and gene expression in healthy donors and patients.

## Discussion

In this study, we analysed genome-wide DNA methylation in the peripheral blood from three WS subjects and three age- and sex-matched controls, identifying 1125 DMPs and 27 DMRs. Pathway enrichment analysis on the list of hypermethylated and hypomethylated DMPs and on the list of DMR provided interesting hints on the epigenetic alterations in WS patients, possibly related with the disease phenotype. Bioactive sphingolipids have been suggested to play a role in ageing and cellular senescence, and in WS, lipid profile is often abnormal [31]. Moreover, glycosphingolipids are elevated during ageing in the mouse brain and liver as well as in human fibroblasts obtained from elderly individuals [32]. On the other hand, longevity is associated with genetic variation in insulin-FOXO pathways [33] and the signalling pathway connecting insulin and FoxO transcription factors provides the most compelling example for a conserved genetic pathway at the interface between ageing and cancer. FoxO proteins are a subgroup of the forkhead family of transcription factors, which regulate the expression of genes involved in several physiological events including apoptosis, cell cycle control, glucose metabolism, oxidative stress resistance and longevity [34, 35]. In response to insulin or growth factors, the protein is phosphorylated by Akt, downstream of PI3K. This constitutes a signal to export FoxO from the nucleus to the cytoplasm, thereby decreasing the expression of FoxO target genes [34, 36]. Dysregulation of the insulin/PI3K/Akt pathway is implicated in several human diseases including cancer, diabetes, cardiovascular diseases and neurological diseases [37, 38]. Insulin signalling pathway regulates ageing in many organisms, ranging from simple invertebrates to mammals, including human [36]. Notably, the enrichment of hypermethylated probes in insulin and FoxO signalling pathways is also consistent with the clinical and metabolic evidences that relate WS to disorders of lipid and liver function [39].

The hypomethylated probes were enriched in PI3K-Akt signalling (HSA-04151) and focal adhesion (HSA-04510) pathways. Even these results underline the importance of deregulated methylation positions we detected. In fact, the role of the PI3K/Akt/FoxO signalling in longevity appears to be well conserved across species [40] and senescence-related morphological alteration is one of the main features of the senescent phenotype and it is deeply dependent to changes of cellular structural determinants in terms of their levels and activities. These determinants included integrins, focal adhesion complexes and small Rho GTPases [41].

Importantly, the alteration of the above-mentioned pathways in WS was fully supported when we correlated gene sets detected as differentially methylated and a publicly available gene expression dataset on WS fibroblasts including ten WS and ten CTR [30]. Indeed, several of the differentially methylated genes belonging to the enriched pathways showed altered RNA expression in fibroblasts from WS patients compared to healthy controls (Fig. 3, Additional file 6: Figure S2).

The small overlap between WS and DKC DMPs suggests that the two progeroid diseases show distinct patterns of epigenetic alterations. However, the *PRDM8* gene resulted hypermethylated in both WS and DKC, although in different CpG sites, suggesting that epigenetic remodelling in different premature ageing syndromes can converge on the same gene.

Interestingly, the gene *ITGA9*, which encodes for an alpha integrin, was previously reported to be hypomethylated and overexpressed in fibroblasts from patients with diffuse and limited SSc compared to fibroblasts from healthy controls [18]. Although in our dataset WS affected the methylation of a distinct CpG site in the body of *ITGA9* (cg22345769, which is not assessed in the study by Altorok et al. [18], as included in the Infinium MethylationEPIC and not in the Infinium450k microarray), it is noteworthy that the methylation and mRNA level of the same gene is altered in both the diseases. In fact, in our bioinformatics analysis, we detected a fold change equal to 1.15 for the differential expression of *ITGA9* (*P* value = 0.037). Furthermore, we found that another gene is implicated in cell-cell and cell-matrix interactions, *ADAM12*, that resulted hypomethylated both in WS and in diffuse and limited SSc [18] and overexpressed (fold change equal to 1.3, *P* value = 0.022) in WS with respect to CTR when we analysed the GSE48761 dataset [30] (Fig. 3). *ADAM12* is involved in the process of fibrosis through enhancing TGF-β signalling pathway [42, 43]. Of note, the peripheral blood from WS patients shared the same hypomethylated CpG site in the body of *ADAM12* with SSc fibroblasts.

Finally, *CERS3* was shown to exhibit differentially methylated status between WS patients and CTRs. A distinct expression profile was also found between normal and WS fibroblasts in the GSE48761 dataset (Fig. 3) [30]. This gene regulates sphingolipid synthesis and is involved in the synthesis of ceramides with ultra-long-chain acyl moieties (ULC-Cers), playing an important role in creating a barrier for the epidermis from the environment [44]. A mutation in CERS3 is responsible of autosomal recessive congenital ichthyosis [45], and interestingly, scleroderma-like changes have been described in different clinical variants of ichthyosis [46]. All these data are of particular interest as WS has some features that are typical SSc signs like skin sclerosis and calcification and ankle ulcerations [47, 48]. Indeed, skin sclerosis is a hallmark of SSc and skin thickening represents the definitive diagnostic criterion of SSc in the vast majority of cases [19–21, 49]. Common to SSc subjects, WS patients show SSc-like skin involvement, calcinosis and skin ulcers; in particular, ulcers are present in 50% of WS patients and are generally the main cutaneous symptom [50, 51]. Anyway, the possibility for WS patients to be diagnosed as SSc was avoided as, based on 2013 ACR/EULAR classification criteria for SSc disease [52], serological analyses performed on subjects enrolled in the present study for anti-ANA and anti-ENA (anti-SS-A, anti-SS-B, anti-SM, anti-RNP, anti-SCL-70 and anti-JO-1) antibodies resulted negative. The presence of serum autoantibodies directed to multiple intracellular antigens is considered the serological hallmark of SSc [53].

The emerging connection in WS and SSc epigenetic alterations that we observed in this study could be related to the fact that the WS patients that we analysed for genome-wide DNA methylation were recruited in the framework of a study on SSc. Although we cannot exclude a priori this hypothesis, it is worth to note that (1) the CpG sites cg17287034 in the *ADAM12* gene and cg06679270 in the *CERS3* gene showed the same DNA methylation trend in the GSE42865 dataset and in ours (we cannot check *ITGA9* because the probe is missing in the Infinium450k design) (Additional file 5) and (2) *ITGA9*, *ADAM12* and *CERS3* showed gene expression changes between WS and normal fibroblasts according to the GSE48761 dataset. As in both the publicly available studies WS patients were not selected on the basis of a SSc-like phenotype, we can suggest that SSc-related epigenetic changes are an intrinsic characteristic of WS. Although further studies should confirm our findings, they could be particularly interesting since recent studies highlight that another progeroid disease, the Hutchinson-Gilford progeria syndrome, is associated with SSc-like skin changes [54, 55] and that SSc-associated fibrosis is considered as an accelerated ageing phenotype [56].

## Conclusions

In summary, we identified for the first time genome-wide DNA methylation changes in the peripheral blood from WS patients, providing important new insight in the pathogenesis of the diseases and emphasizing the potential role of DNA methylation changes in Werner disorder.

## Additional files

Additional file 1: Figure S1. Principal Component Analysis for the DNA methylation levels of the probes included in the InfiniumEPIC beadchip in WS and CTR. (TIFF 1825 kb)

Additional file 2: List of DMPs between WS and CTR. (XLS 531 kb)

Additional file 3: DNA methylation profiles of the 27 DMRs between WS and CTR. For each DMR, the title of the plot reports the name of the gene/genes in which the DMR maps, the not-adjusted *P* value of the ANOVA comparison between WS and CTR, and the length of the DMR in base pairs. Below the plot, the name of the CpG island and the position of the DMR respect to the CpG island are reported. (PDF 58 kb)

Additional file 4: List of DMRs between WS and CTR. (XLS 60 kb)

Additional file 5: Boxplots of DNA methylation values of the CpG probes in the genes belonging to the enriched pathways in naïve B cells, CTR LCL, WS LCL from the GSE42865 dataset and in CTR and WS whole blood samples assessed in this study. The brown dots correspond to the individual samples. For each CpG probe, the title of the plot reports the name of the CpG probe, the name of the gene/genes in which the probe maps and, if present, the name of the CpG island and the position of the probe respect to the CpG island. (PDF 41 kb)

Additional file 6: Figure S2. Differential expression of several genes belonging to the enriched pathways resulted based on publicly available dataset on WS fibroblasts analyses. (TIFF 2607 kb)

Additional file 7: Relationship between DNA methylation of DMPs and DMRs in the present dataset and RNA expression in the GSE48761 dataset. (XLS 68 kb)

## Abbreviations

DKC: Dyskeratosis congenita
DMPs: Differentially methylated positions
DMRs: Differentially methylated regions
LCLs: Lymphoblastoid cell lines
NVC: Nailfold video capillaroscopy
SSc: Systemic sclerosis
WRN: Werner syndrome gene
WS: Werner syndrome
