# Supplementary figures and images for: Genome-wide DNA methylation analysis in blood cells from patients with Werner syndrome
Source: Clin Epigenetics. 2017 Aug 30;9:92. doi: 10.1186/s13148-017-0389-4 (PMC5577832; doi:10.1186/s13148-017-0389-4)

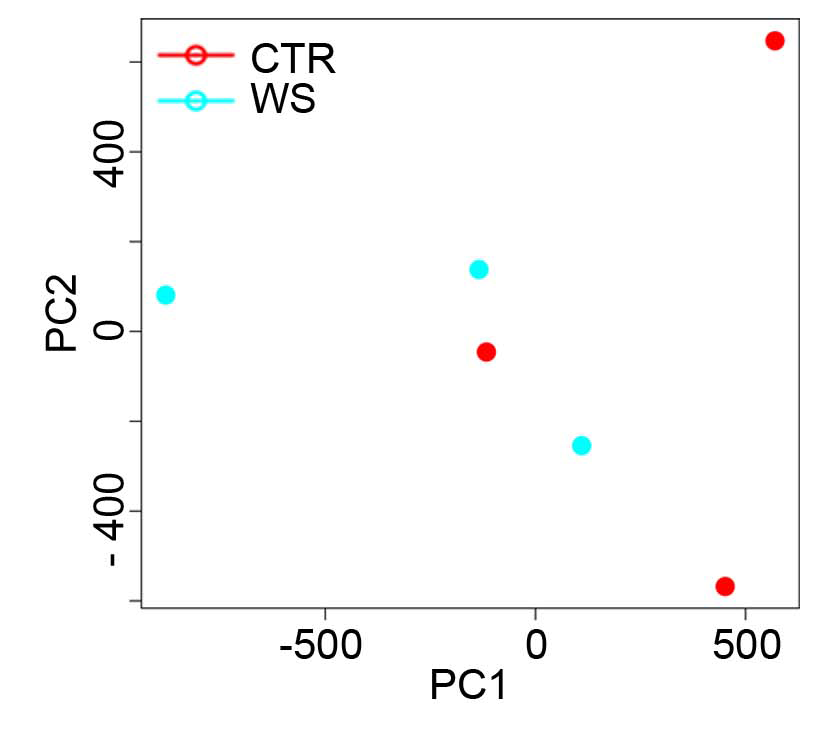

Supplement: Supplementary file 1 — Principal Component Analysis for the DNA methylation levels of the probes included in the InfiniumEPIC beadchip in WS and CTR. (TIFF 1825 kb) [file 13148_2017_389_MOESM1_ESM.tif]

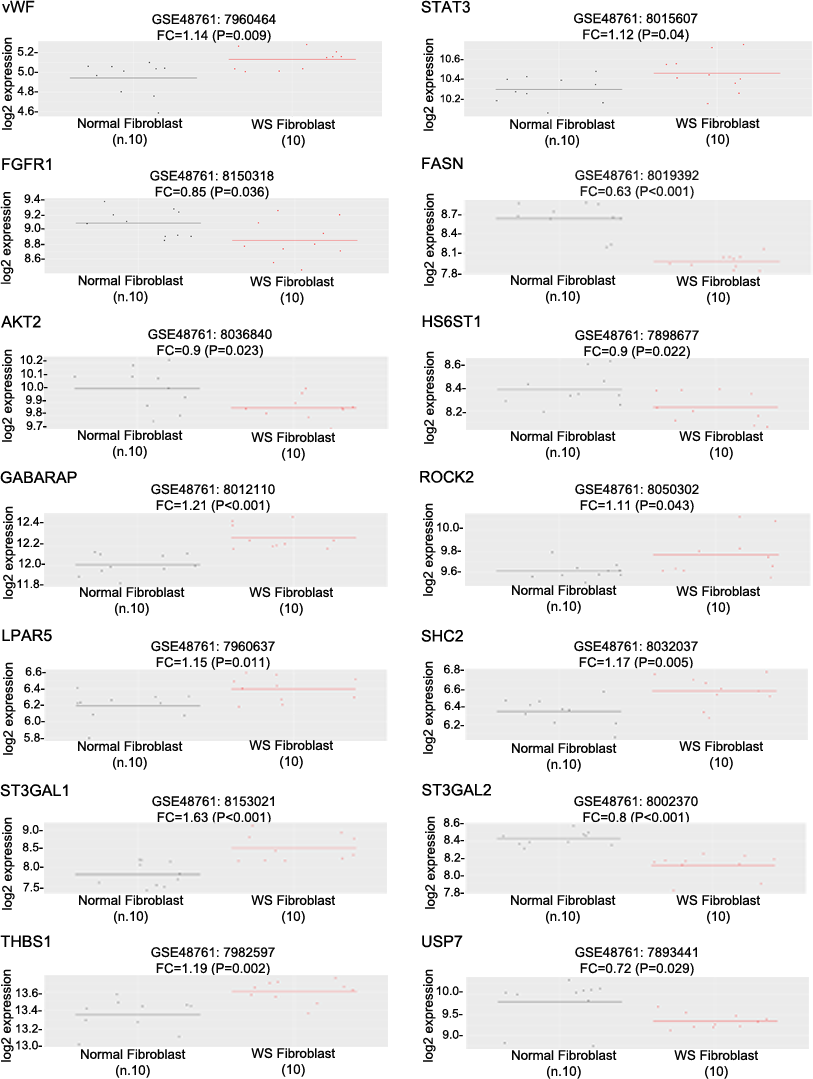

Supplement: Supplementary file 6 — Differential expression of several genes belonging to the enriched pathways resulted based on publicly available dataset on WS fibroblasts analyses. (TIFF 2607 kb) [file 13148_2017_389_MOESM6_ESM.tif]
