# Supplementary material for: Genome-wide DNA methylation analysis in blood cells from patients with Werner syndrome
Source: Clin Epigenetics. 2017 Aug 30;9:92. doi: 10.1186/s13148-017-0389-4 (PMC5577832; doi:10.1186/s13148-017-0389-4)
Supplement: Supplementary file 3 — DNA methylation profiles of the 27 DMRs between WS and CTR. For each DMR, the title of the plot reports the name of the gene/genes in which the DMR maps, the not-adjusted P value of the ANOVA comparison between WS and CTR, and the length of the DMR in base pairs. Below the plot, the name of the CpG island and the position of the DMR respect to the CpG island are reported. (PDF 58 kb) [file 13148_2017_389_MOESM3_ESM.pdf]

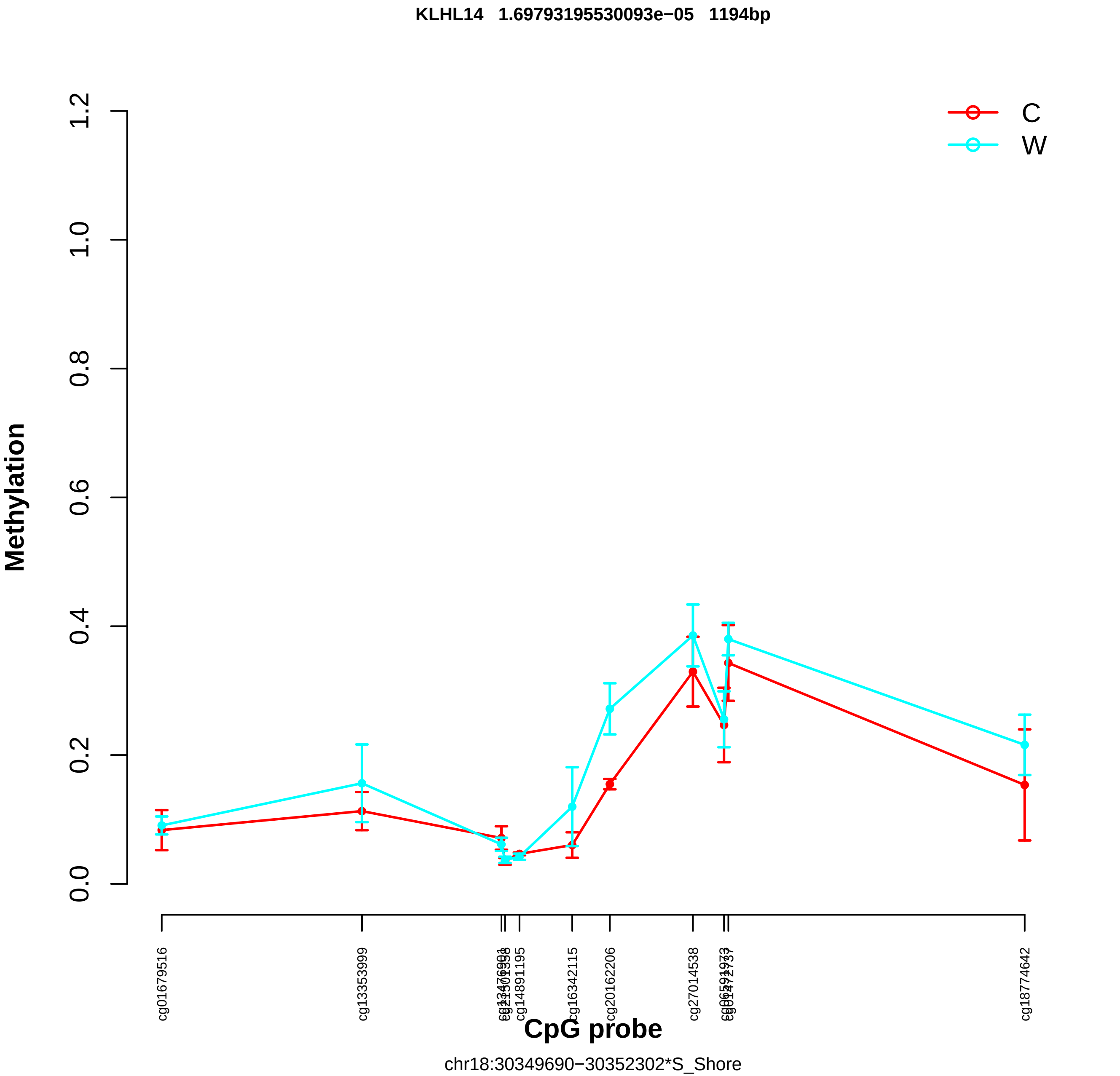
KLHL14 1.69793195530093e−05 1194bp
Methylation
0.0
0.2
0.4
0.6
0.8
1.0
1.2
C
W
cg01679516
cg13353999
cg14891195
cg16342115
cg20162206
cg27014538
cg18774642
CpG probe
chr18:30349690−30352302*S_Shore

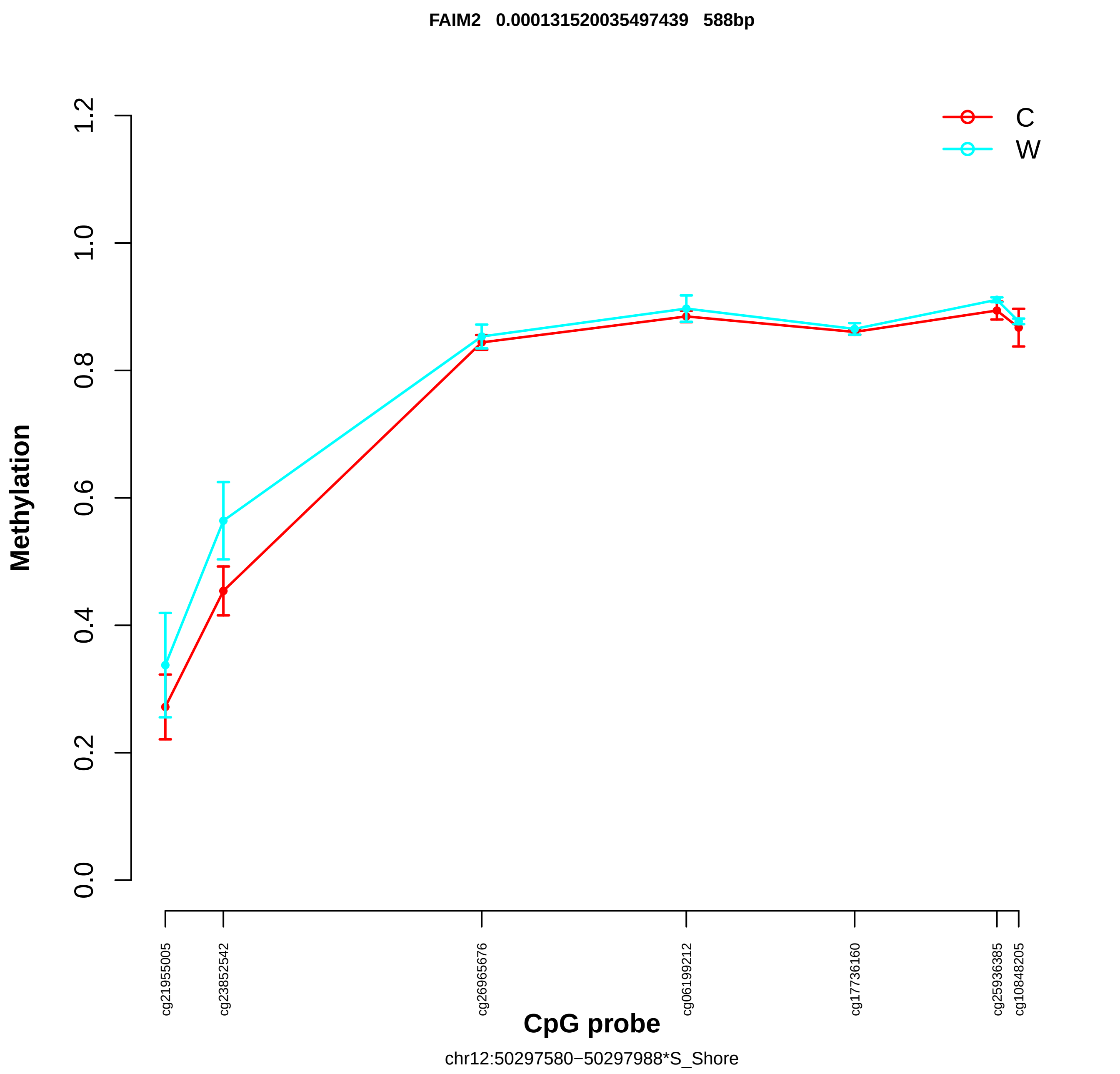

FAIM2 0.000131520035497439 588bp
Methylation
1.2
1.0
0.8
0.6
0.4
0.2
0.0
C
W
cg21955005
cg23852542
cg26965676
cg06199212
cg17736160
cg25936385
cg10848205
CpG probe
chr12:50297580−50297988*S_Shore

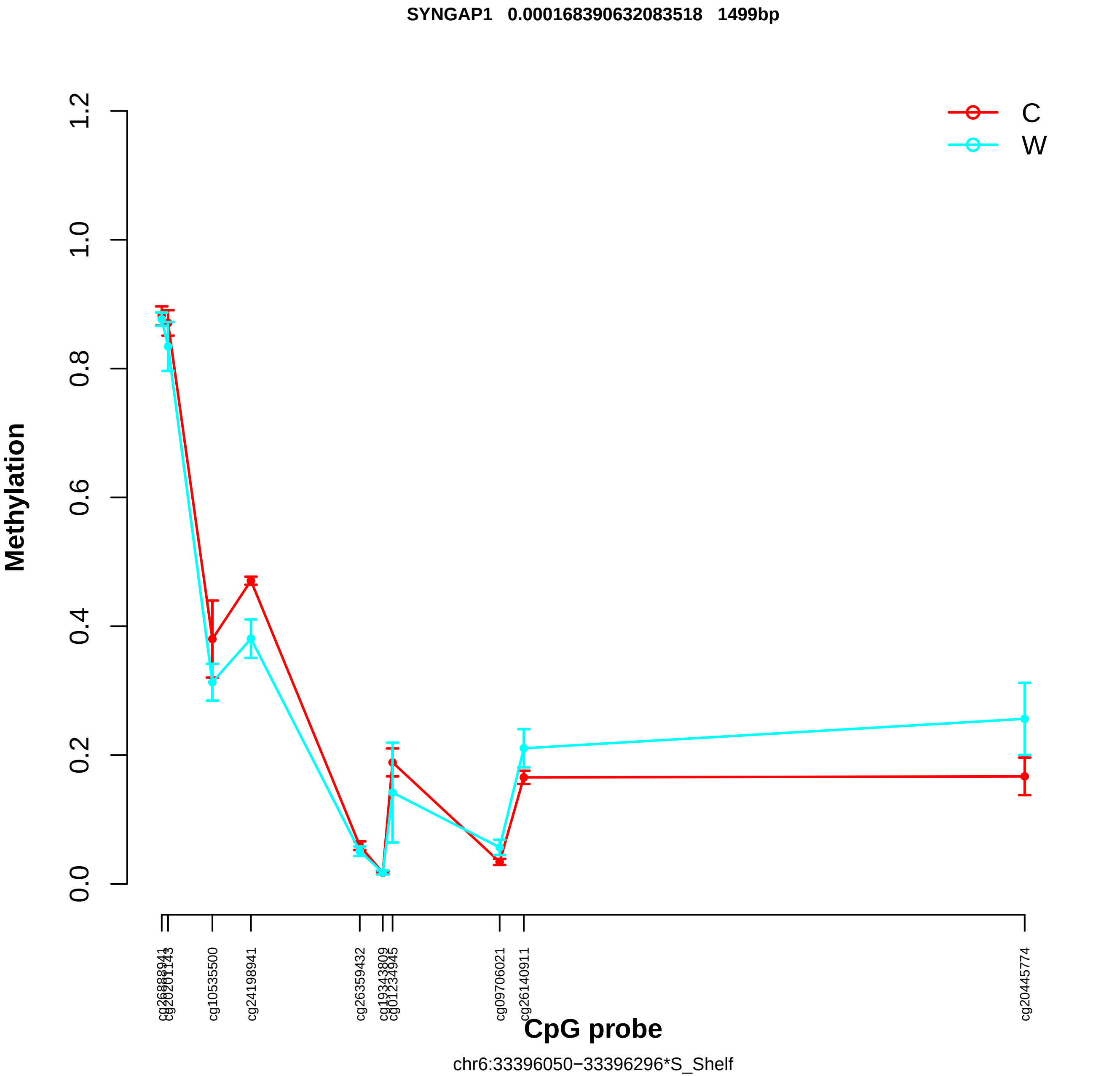
SYNGAP1 0.000168390632083518 1499bp
Methylation
0.0
0.2
0.4
0.6
0.8
1.0
1.2
C
W
cg20201143
cg10535500
cg24198941
cg26359432
cg19343809
cg01234945
cg09706021
cg26140911
cg20445774
CpG probe
chr6:33396050−33396296*S_Shelf

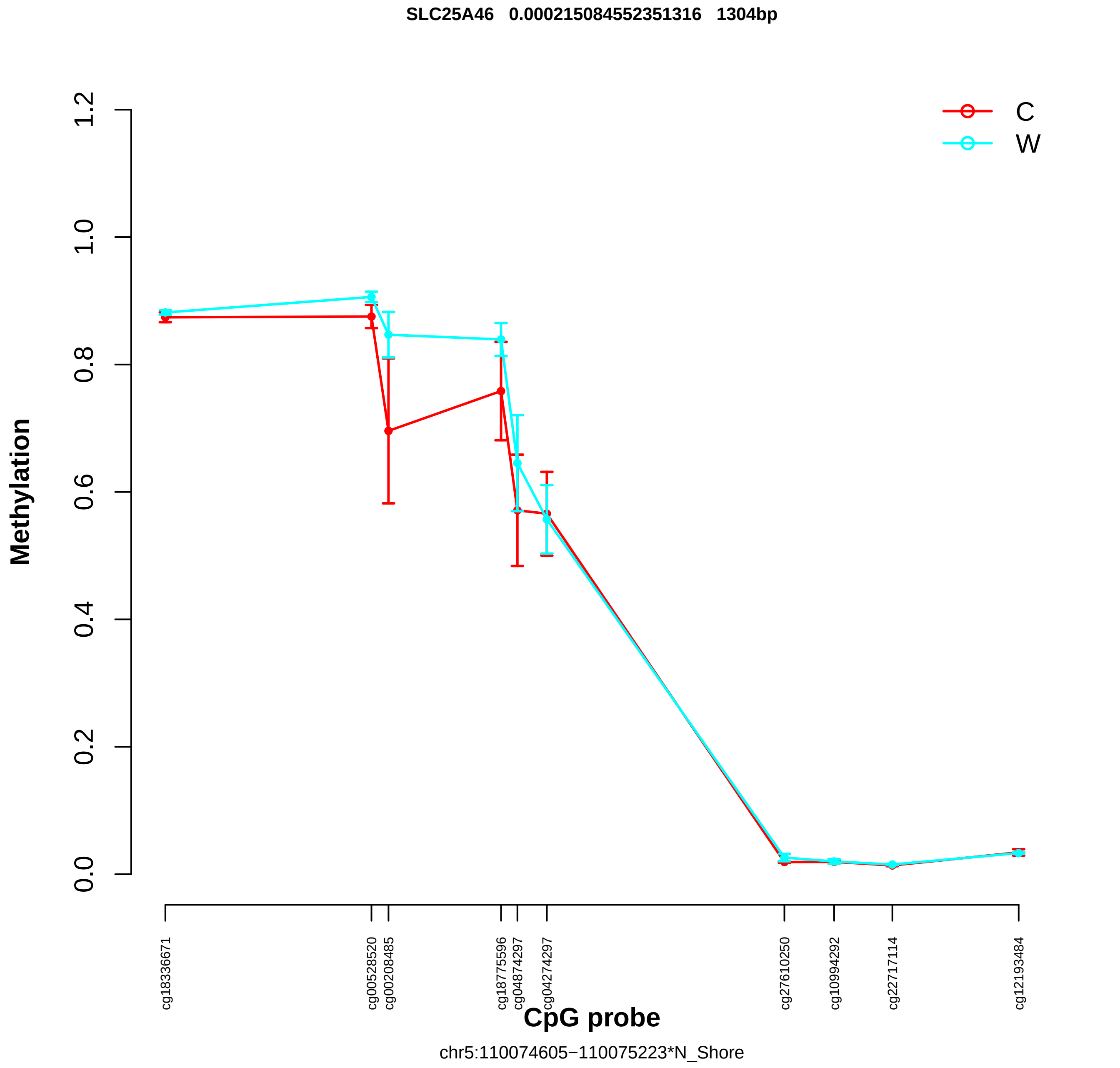
SLC25A46 0.000215084552351316 1304bp
Methylation
1.2
1.0
0.8
0.6
0.4
0.2
0.0
C
W
cg18336671
cg00528520
cg00208485
cg18775596
cg04874297
cg04274297
cg27610250
cg10994292
cg22717114
cg12193484
CpG probe
chr5:110074605−110075223*N_Shore

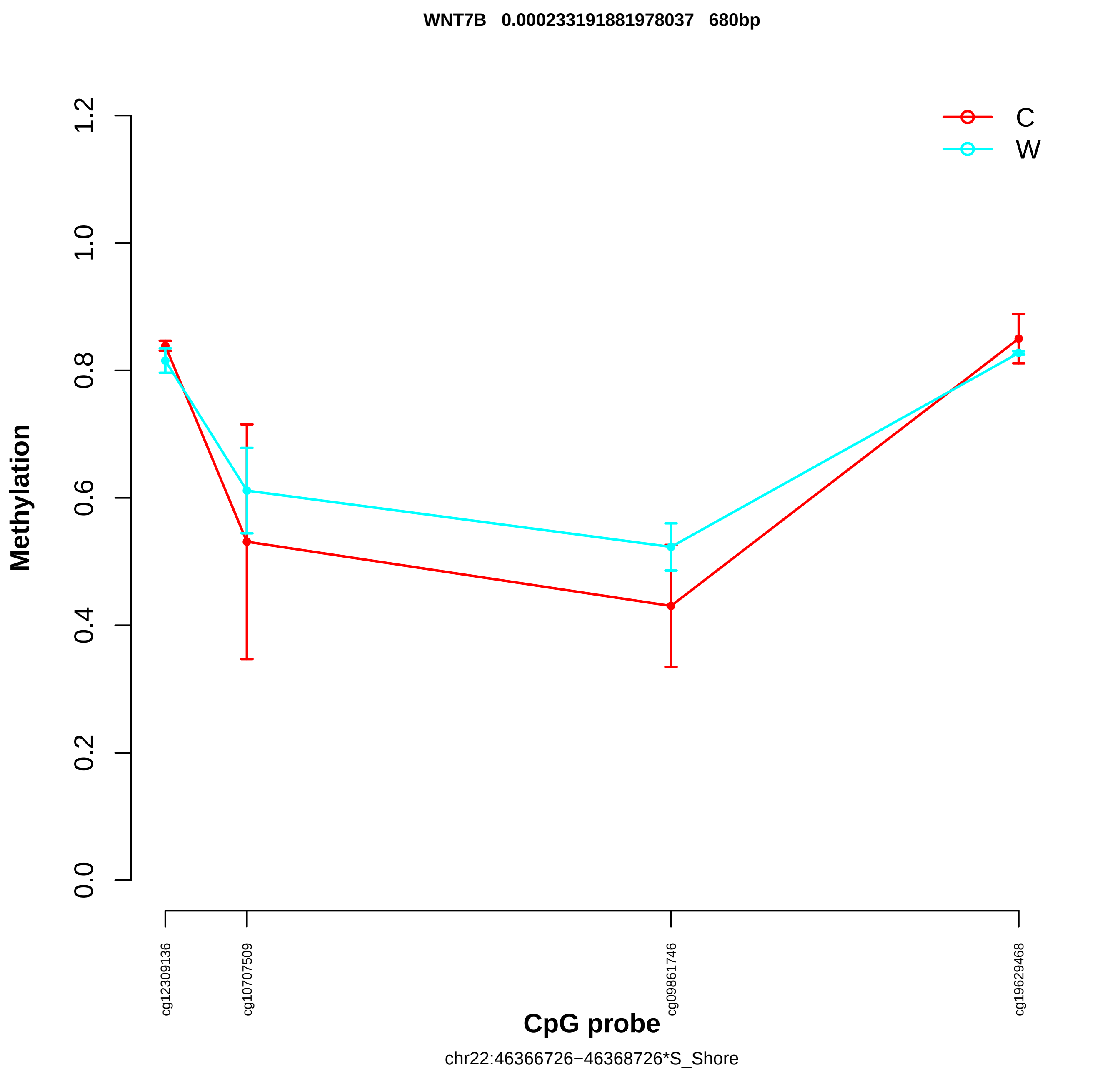
WNT7B 0.000233191881978037 680bp
1.2
1.0
0.8
0.6
0.4
0.2
0.0
Methylation
C
W
cg12309136
cg10707509
cg09861746
cg19629468
CpG probe
chr22:46366726−46368726*S_Shore

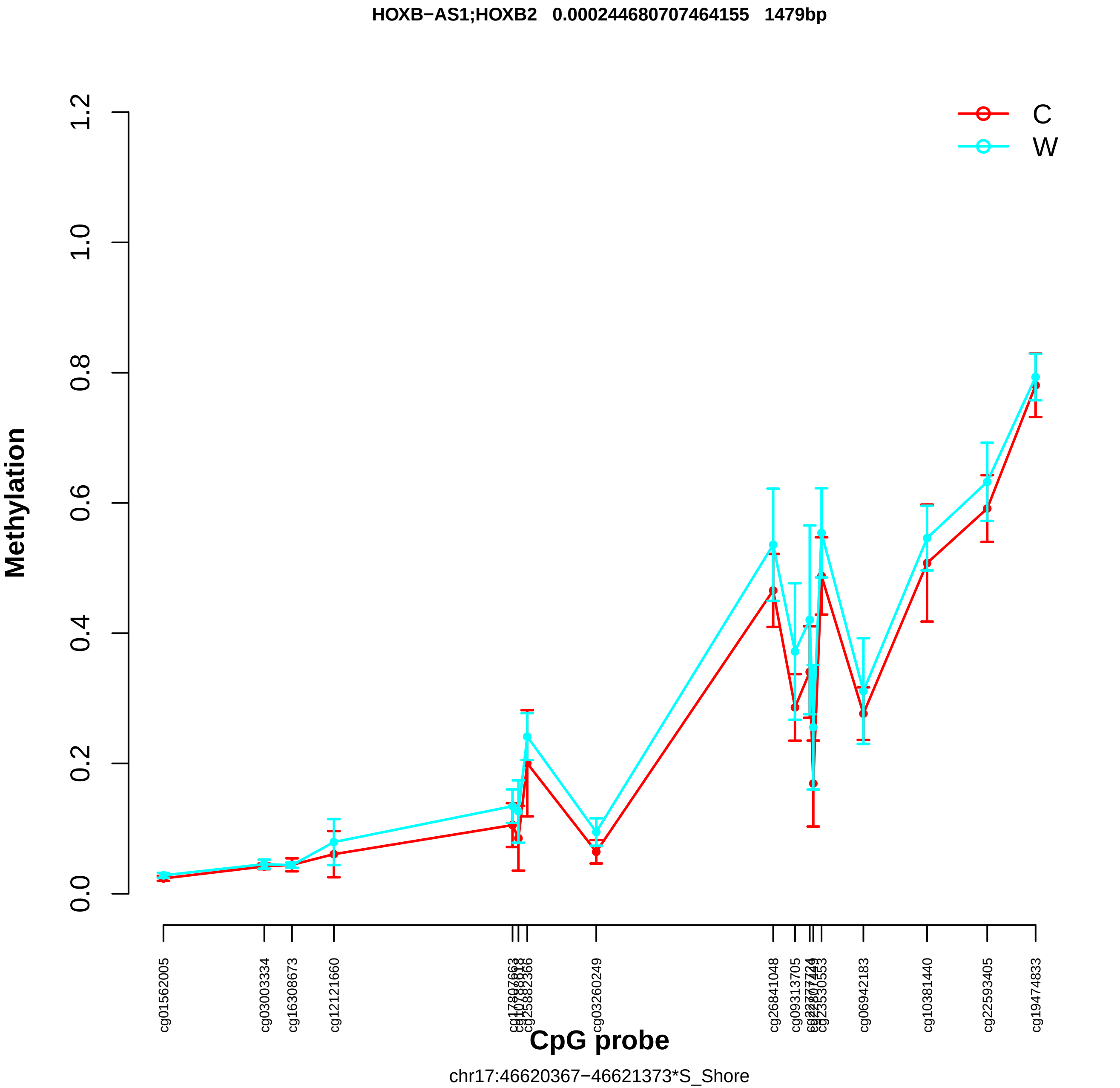

HOXB−AS1;HOXB2 0.000244680707464155 1479bp
Methylation
0.0
0.2
0.4
0.6
0.8
1.0
1.2
C
W
cg01562005
cg03003334
cg16308673
cg12121660
cg25882366
cg03260249
cg26841048
cg09313705
cg25530553
cg06942183
cg10381440
cg22593405
cg19474833
CpG probe
chr17:46620367−46621373*S_Shore

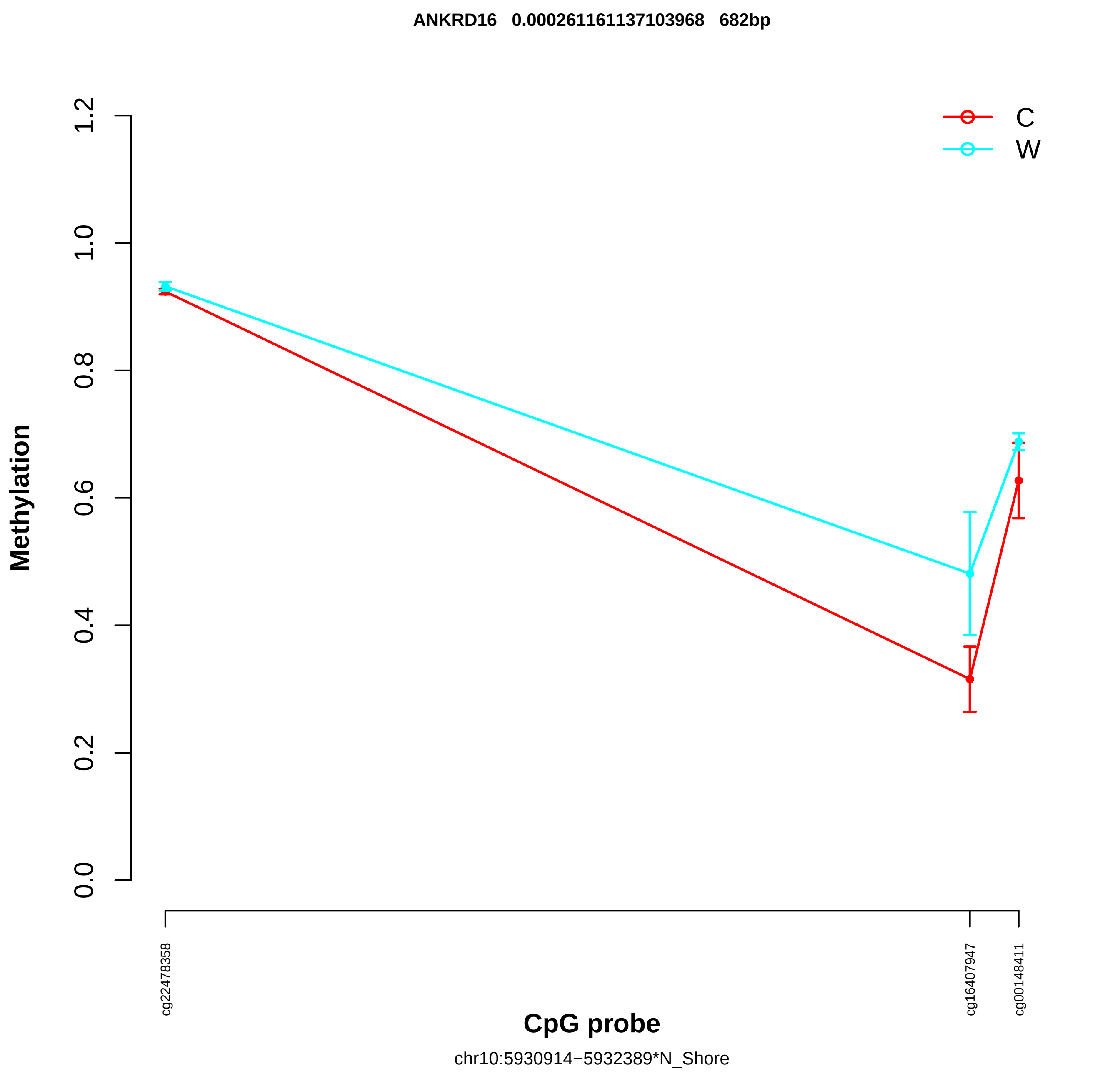

ANKRD16 0.000261161137103968 682bp
Methylation
1.2
1.0
0.8
0.6
0.4
0.2
0.0
C
W
cg22478358
cg16407947
cg00148411
CpG probe
chr10:5930914−5932389*N_Shore

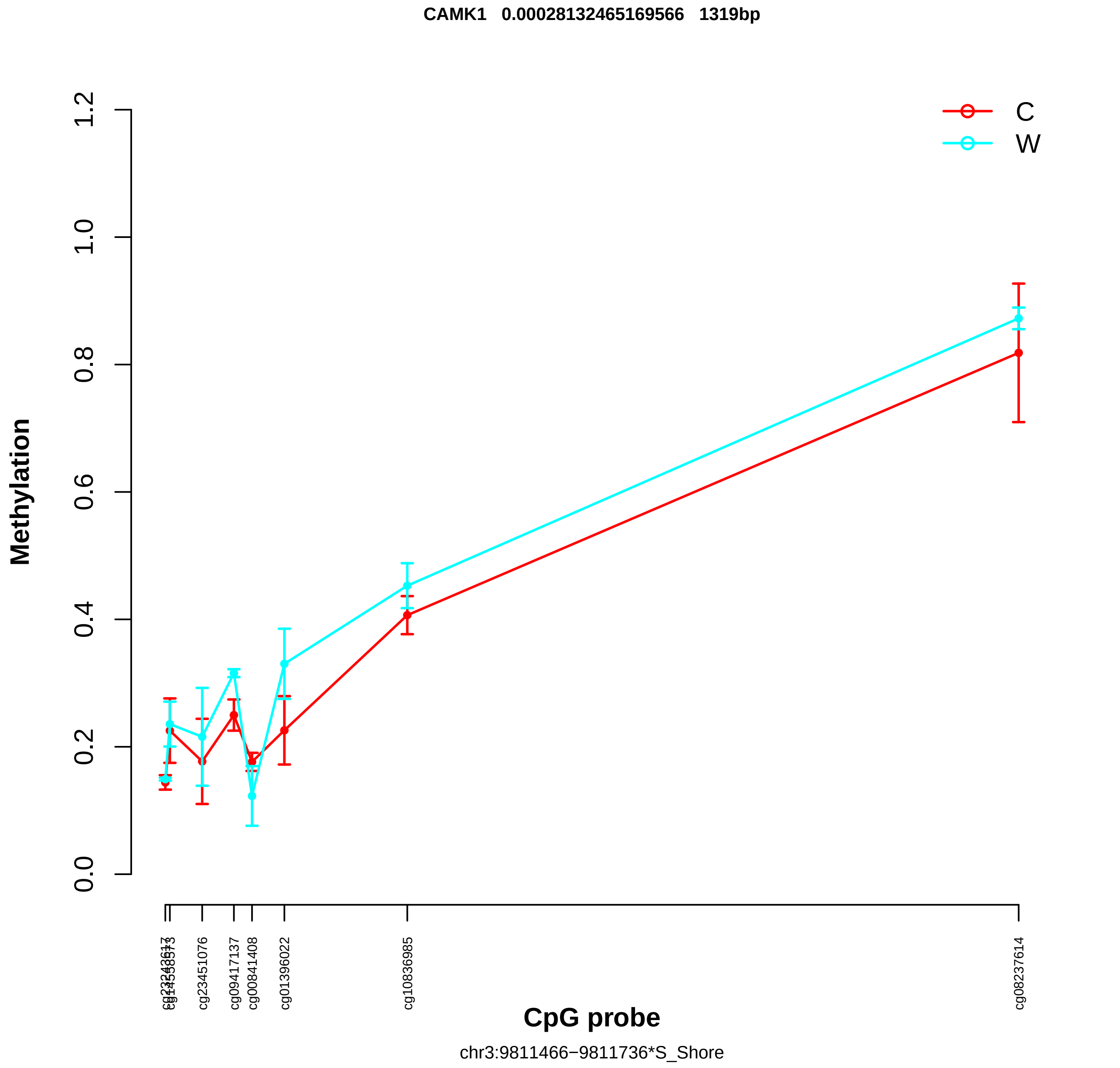
CAMK1 0.00028132465169566 1319bp
Methylation
0.0
0.2
0.4
0.6
0.8
1.0
1.2
C
W
cg23451076
cg09417137
cg00841408
cg01396022
cg10836985
cg08237614
CpG probe
chr3:9811466−9811736*S_Shore

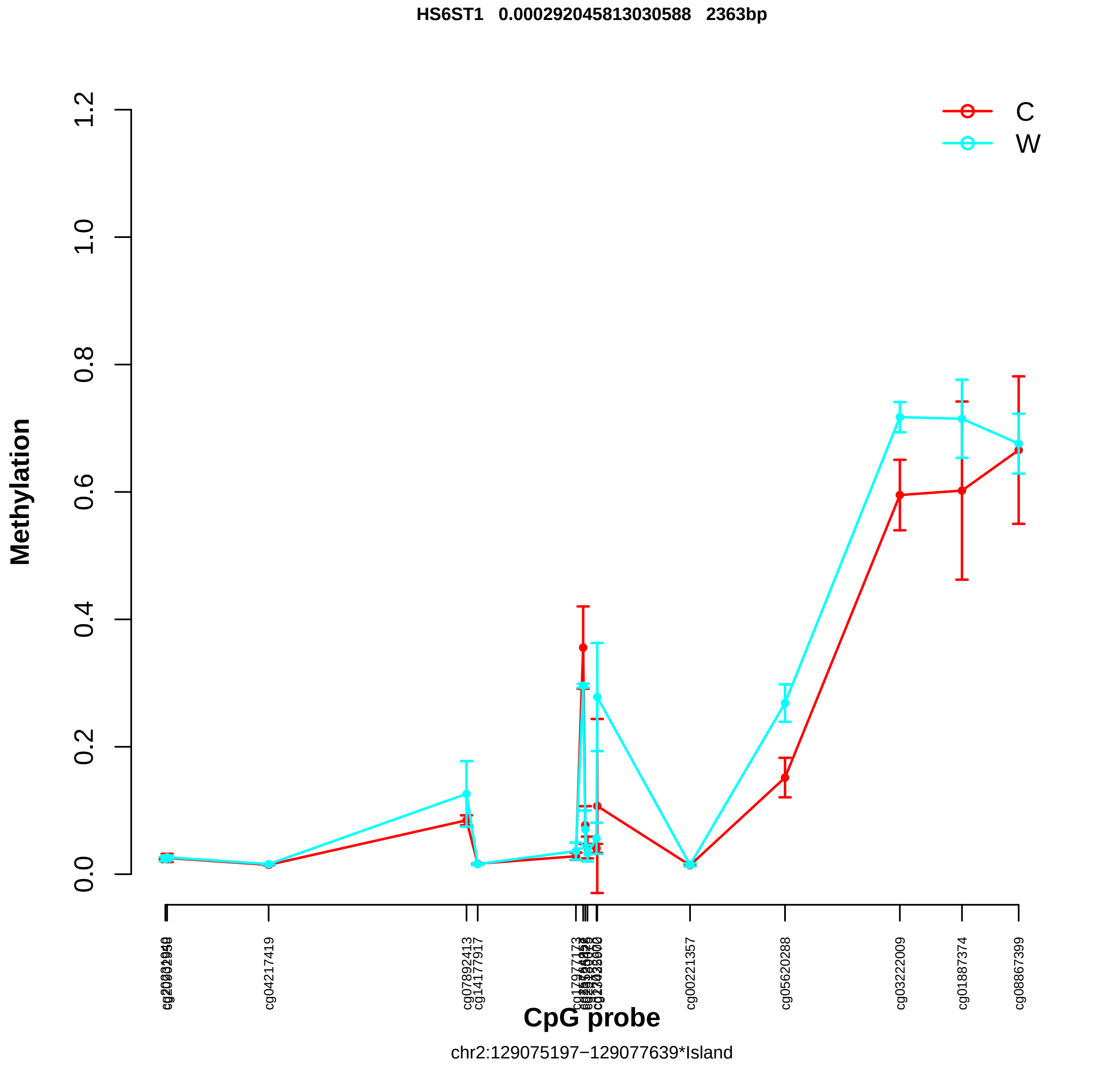
HS6ST1 0.000292045813030588 2363bp
Methylation
0.0
0.2
0.4
0.6
0.8
1.0
1.2
C
W
cg04217419
cg07892413
cg14177917
cg00221357
cg05620288
cg03222009
cg01887374
cg08867399
CpG probe
chr2:129075197−129077639*Island

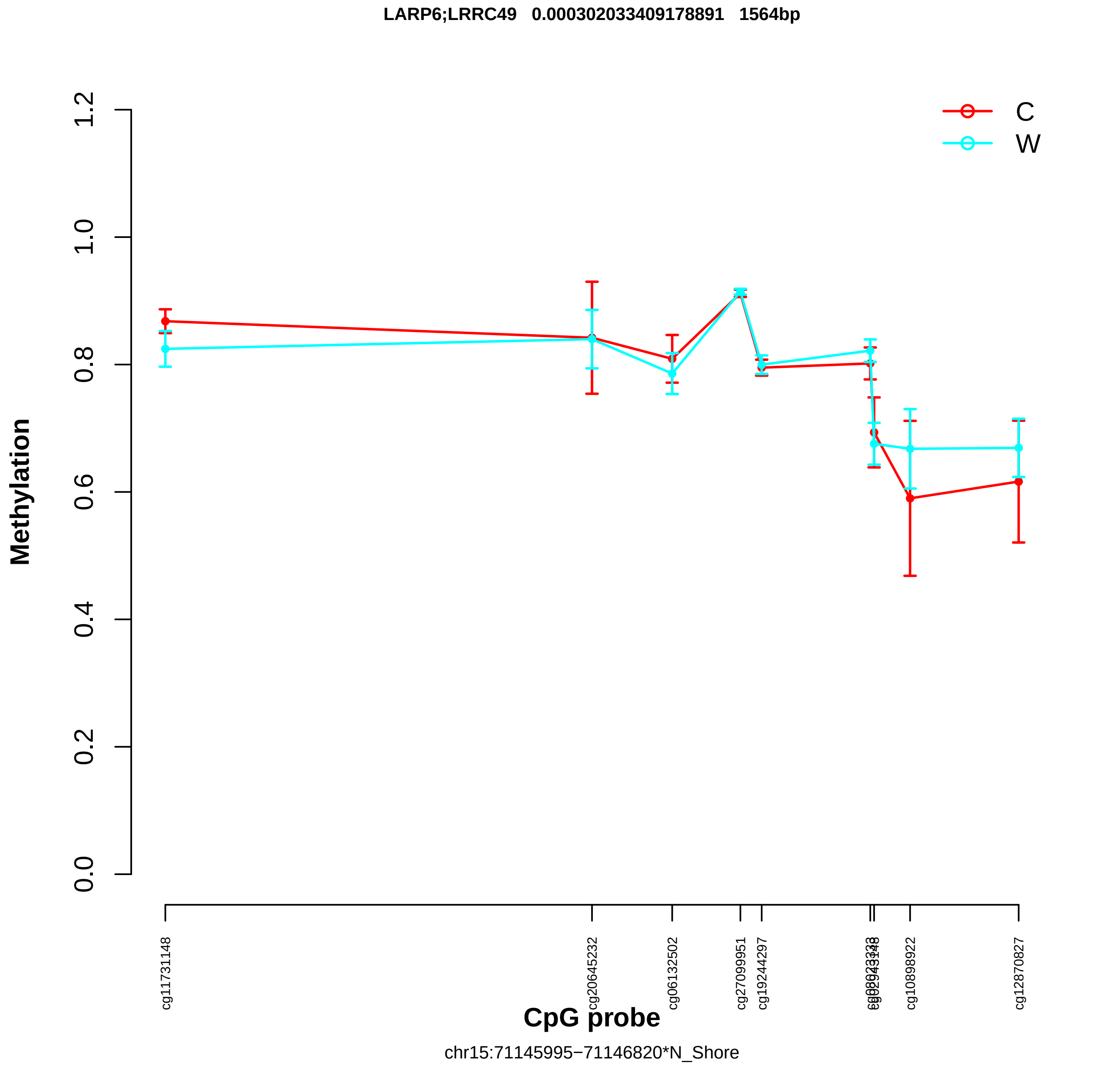
LARP6;LRRC49 0.000302033409178891 1564bp
C
W
Methylation
0.0
0.2
0.4
0.6
0.8
1.0
1.2
cg11731148
cg20645232
cg06132502
cg27099951
cg19244297
cg10898922
cg12870827
CpG probe
chr15:71145995−71146820*N_Shore

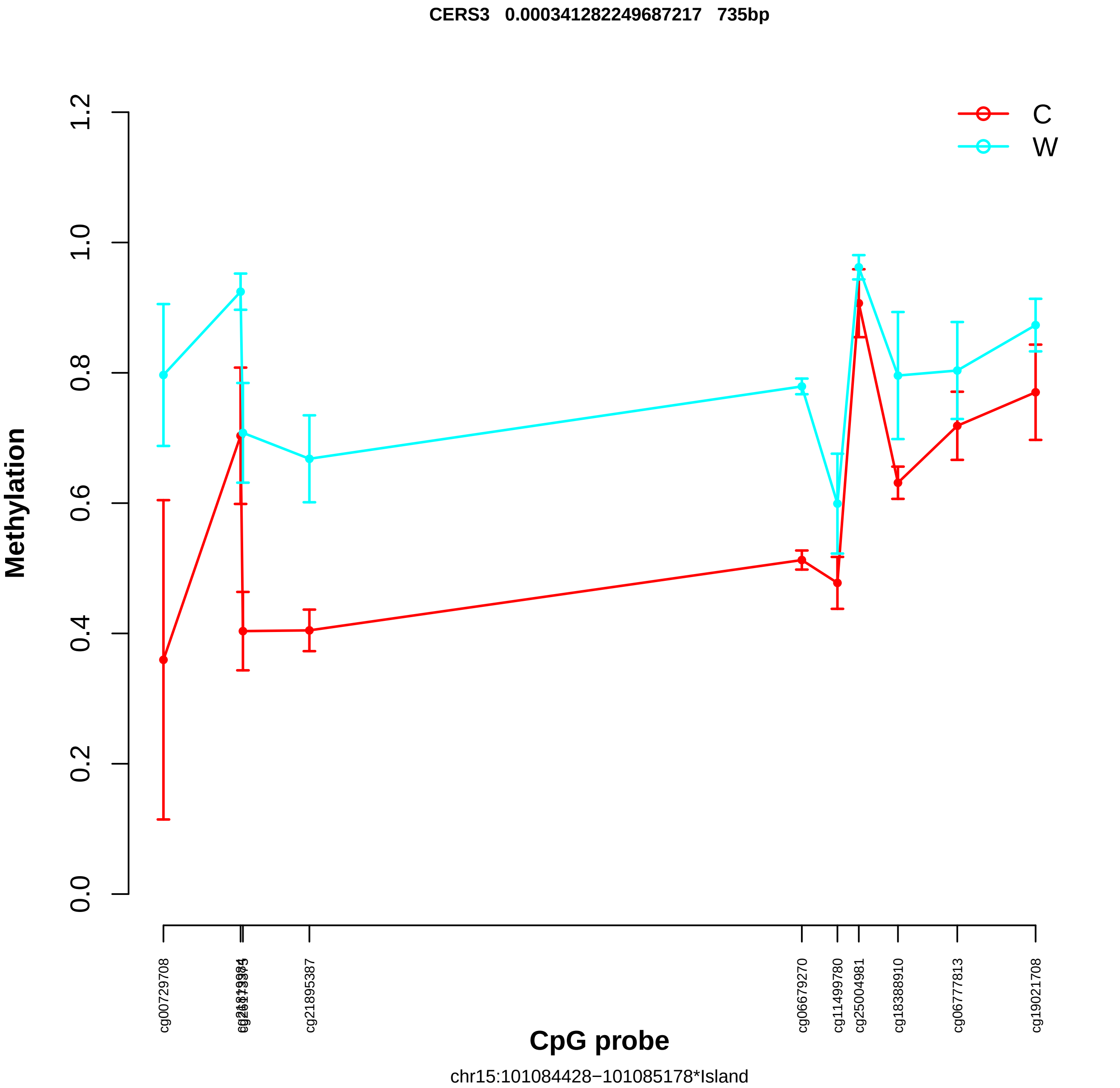
CERS3 0.000341282249687217 735bp
C
W
Methylation
1.2
1.0
0.8
0.6
0.4
0.2
0.0
cg00729708
cg21895387
cg06679270
cg11499780
cg25004981
cg18388910
cg06777813
cg19021708
CpG probe
chr15:101084428−101085178*Island

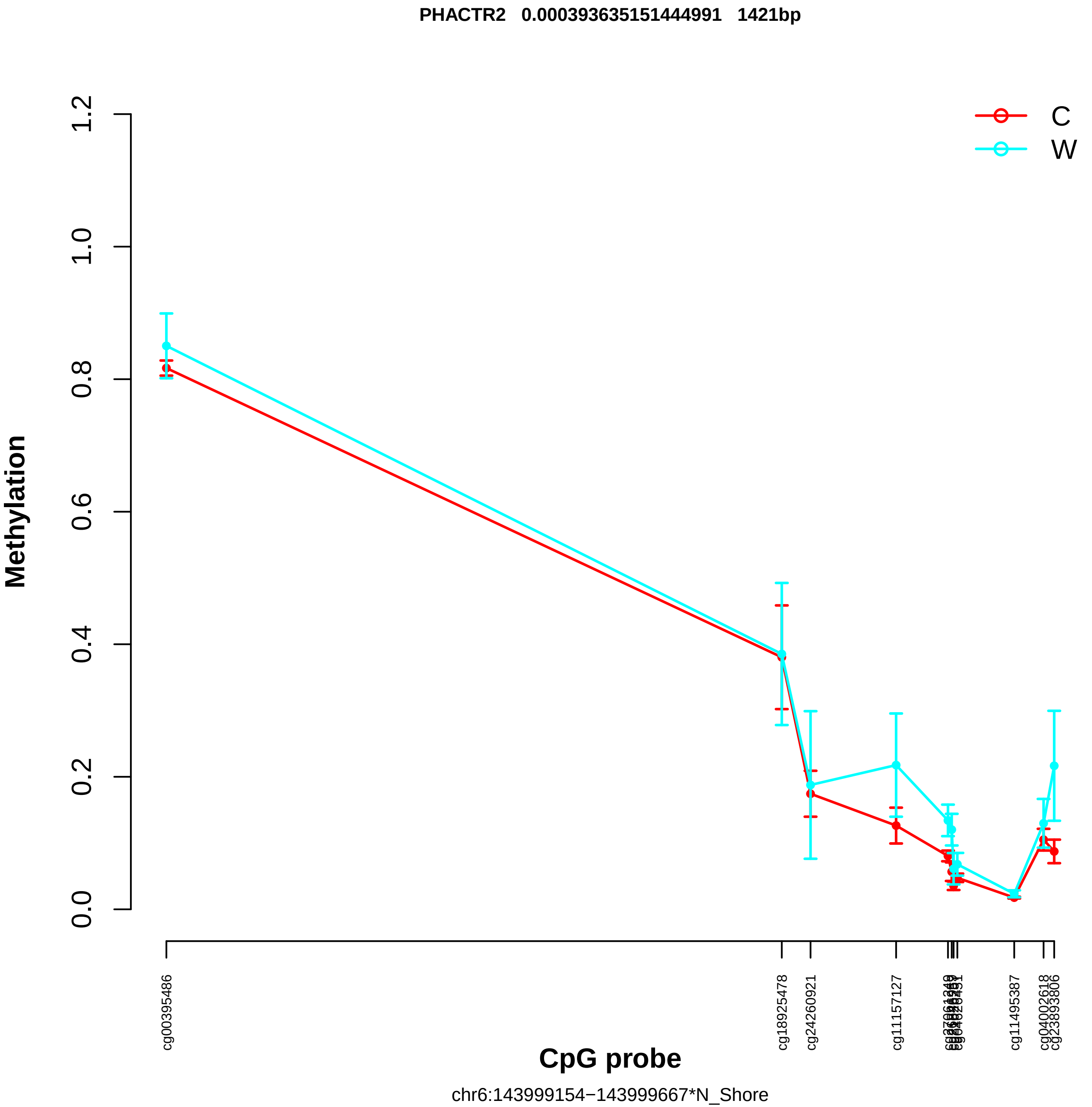

PHACTR2 0.000393635151444991 1421bp
C
W
Methylation
1.2
1.0
0.8
0.6
0.4
0.2
0.0
cg00395486
cg18925478
cg24260921
cg11157127
cg11495387
cg04002618
cg23893806
CpG probe
chr6:143999154−143999667*N_Shore

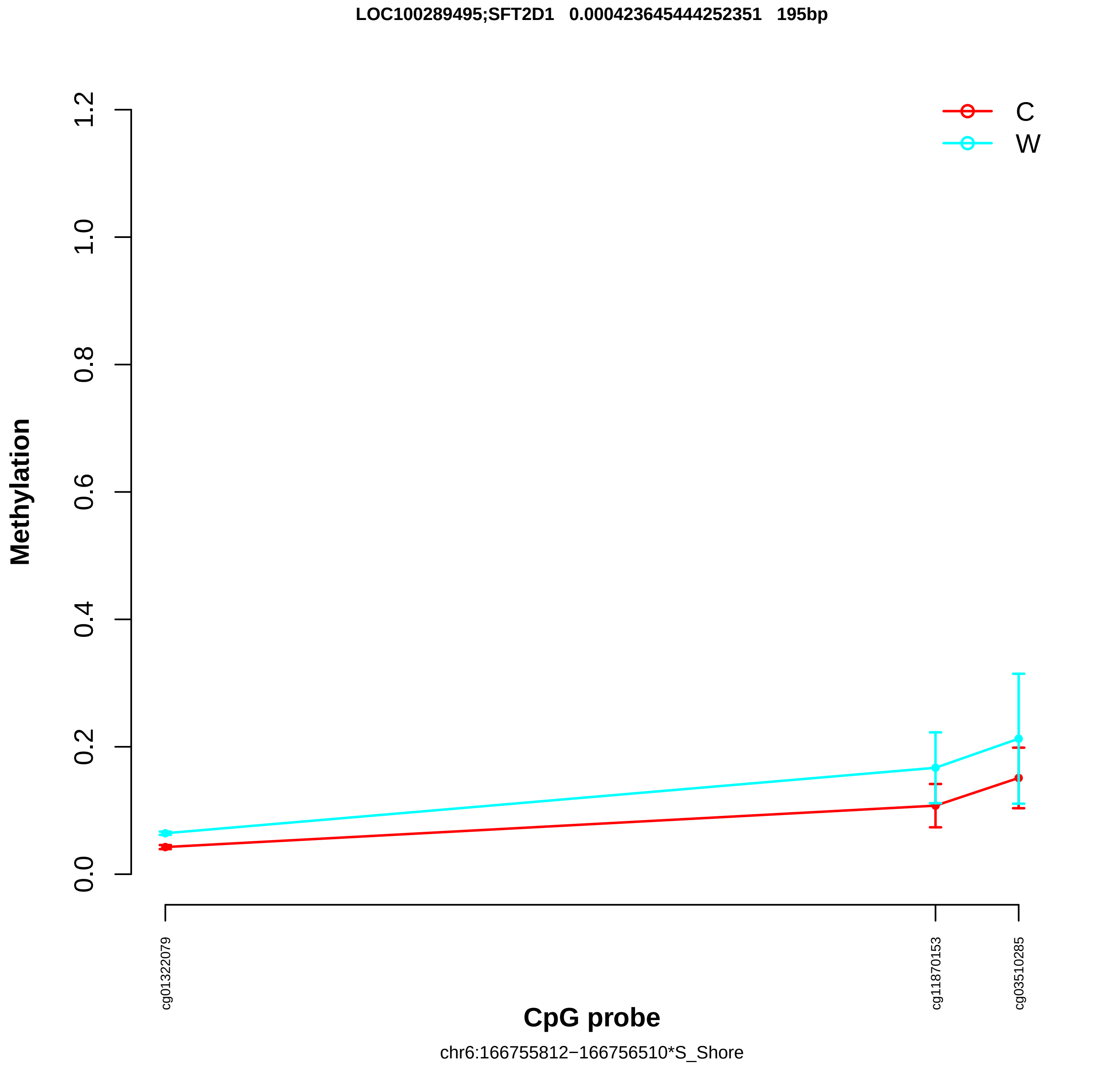
LOC100289495;SFT2D1 0.000423645444252351 195bp
Methylation
0.0
0.2
0.4
0.6
0.8
1.0
1.2
C
W
cg01322079
cg11870153
cg03510285
CpG probe
chr6:166755812−166756510*S_Shore

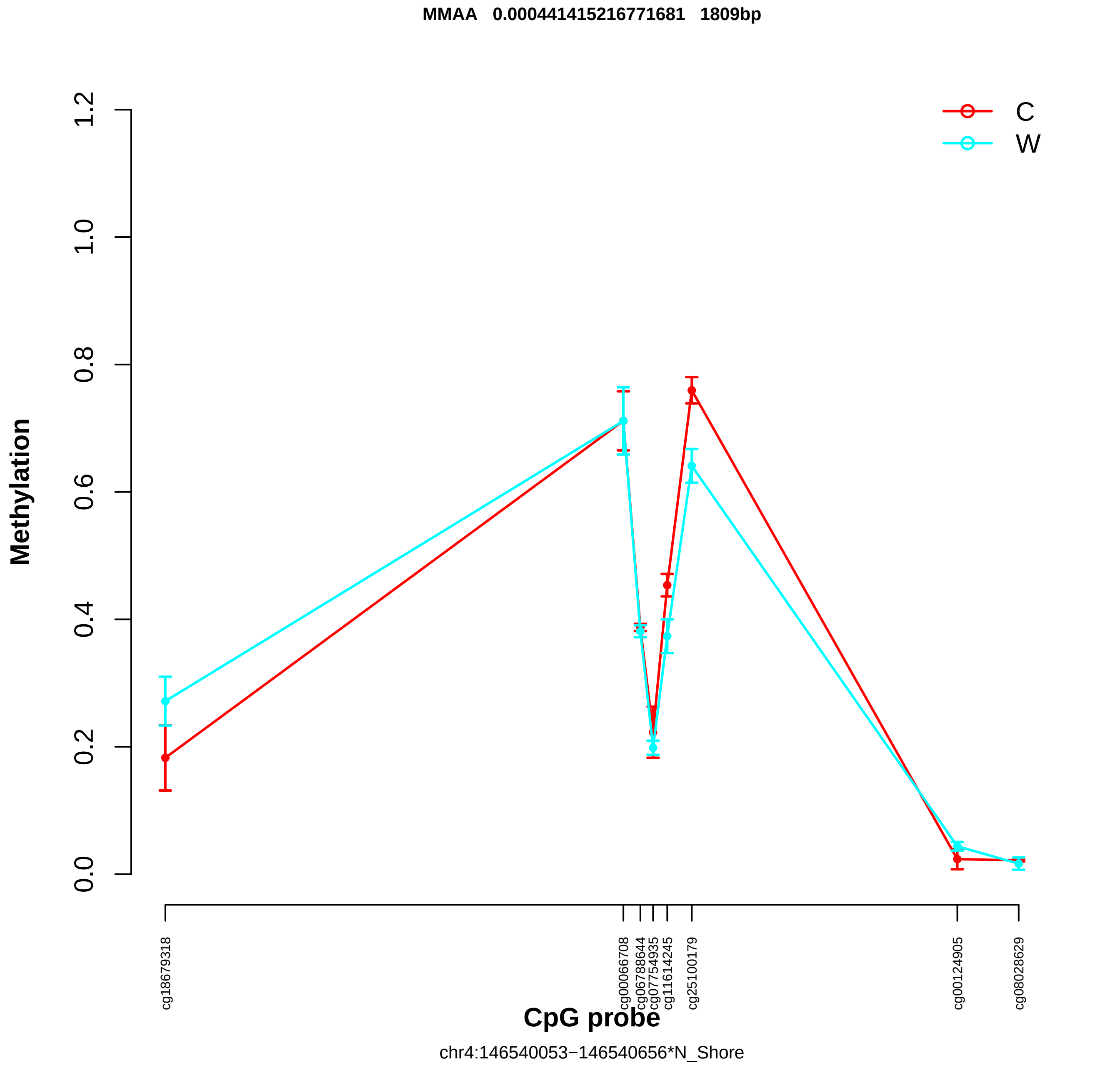
MMAA 0.000441415216771681 1809bp
C
W
Methylation
0.0
0.2
0.4
0.6
0.8
1.0
1.2
cg18679318
cg00066708
cg06788644
cg07754935
cg11614245
cg25100179
cg00124905
cg08028629
CpG probe
chr4:146540053−146540656*N_Shore

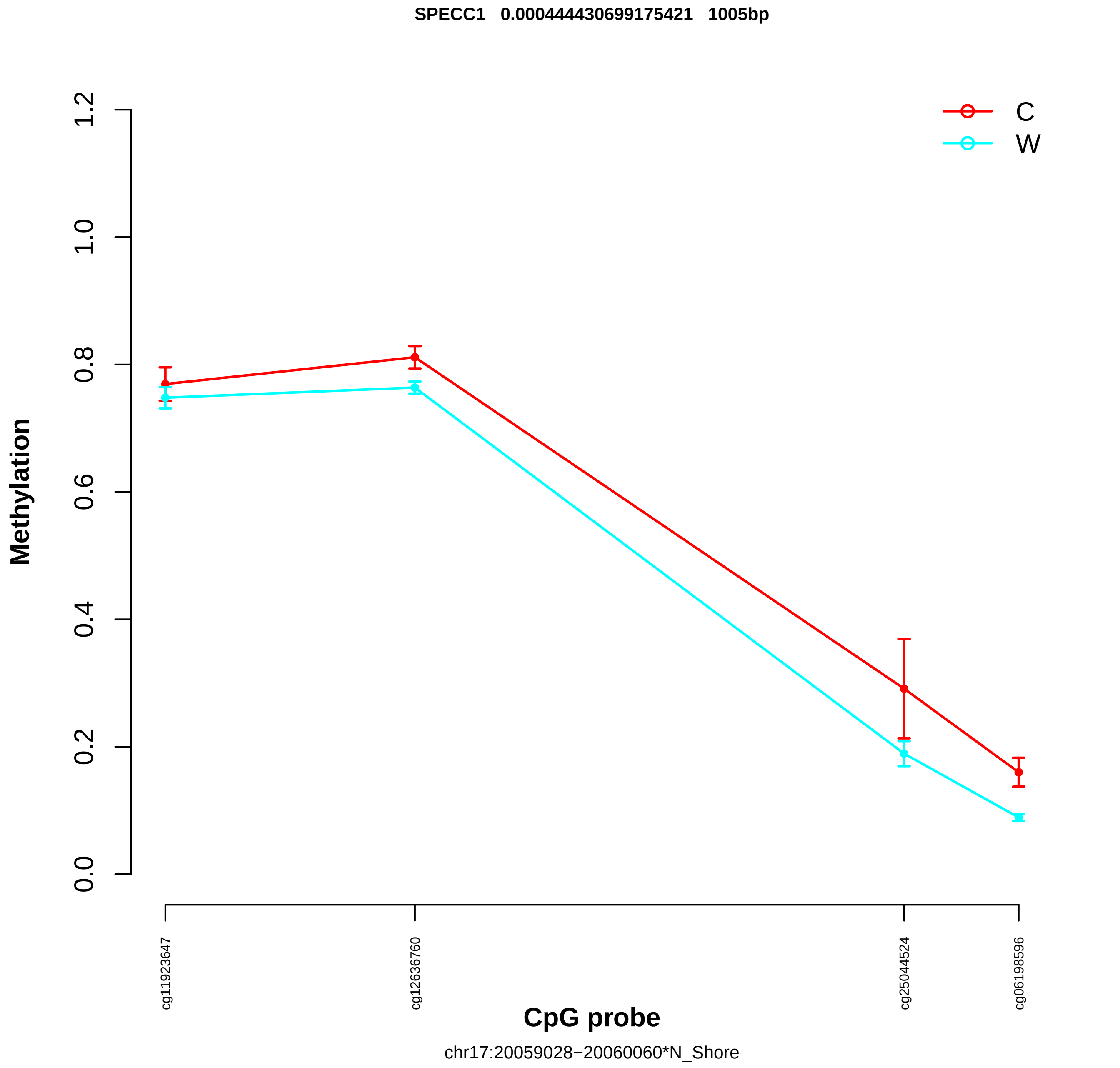
SPECC1 0.000444430699175421 1005bp
Methylation
CpG probe
chr17:20059028−20060060*N_Shore
C
W
0.0
0.2
0.4
0.6
0.8
1.0
1.2
cg11923647
cg12636760
cg25044524
cg06198596

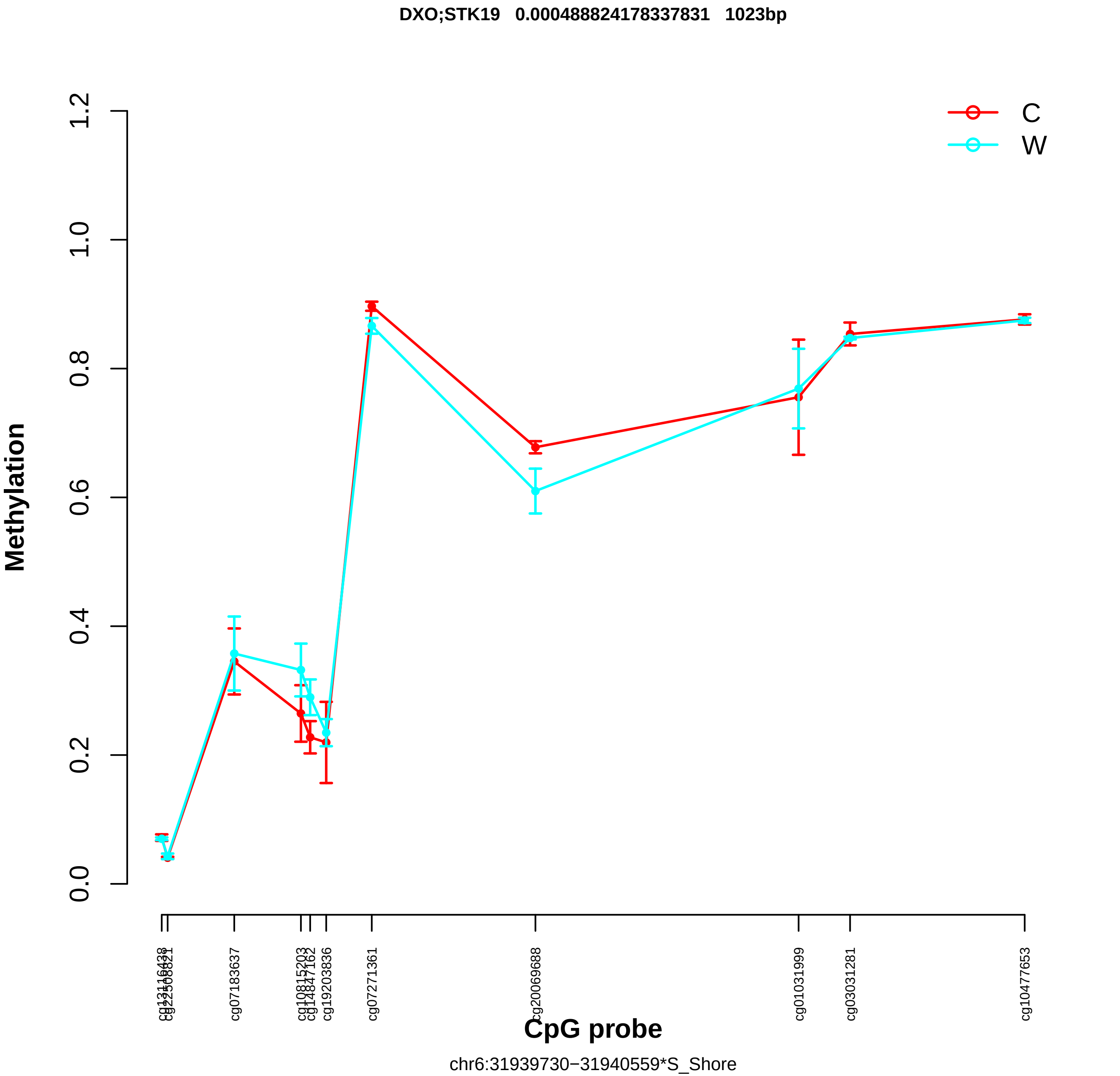
DXO;STK19 0.000488824178337831 1023bp
Methylation
0.0
0.2
0.4
0.6
0.8
1.0
1.2
C
W
cg07183637
cg10815203
cg14847162
cg19203836
cg07271361
cg20069688
cg01031999
cg03031281
cg10477653
CpG probe
chr6:31939730−31940559*S_Shore

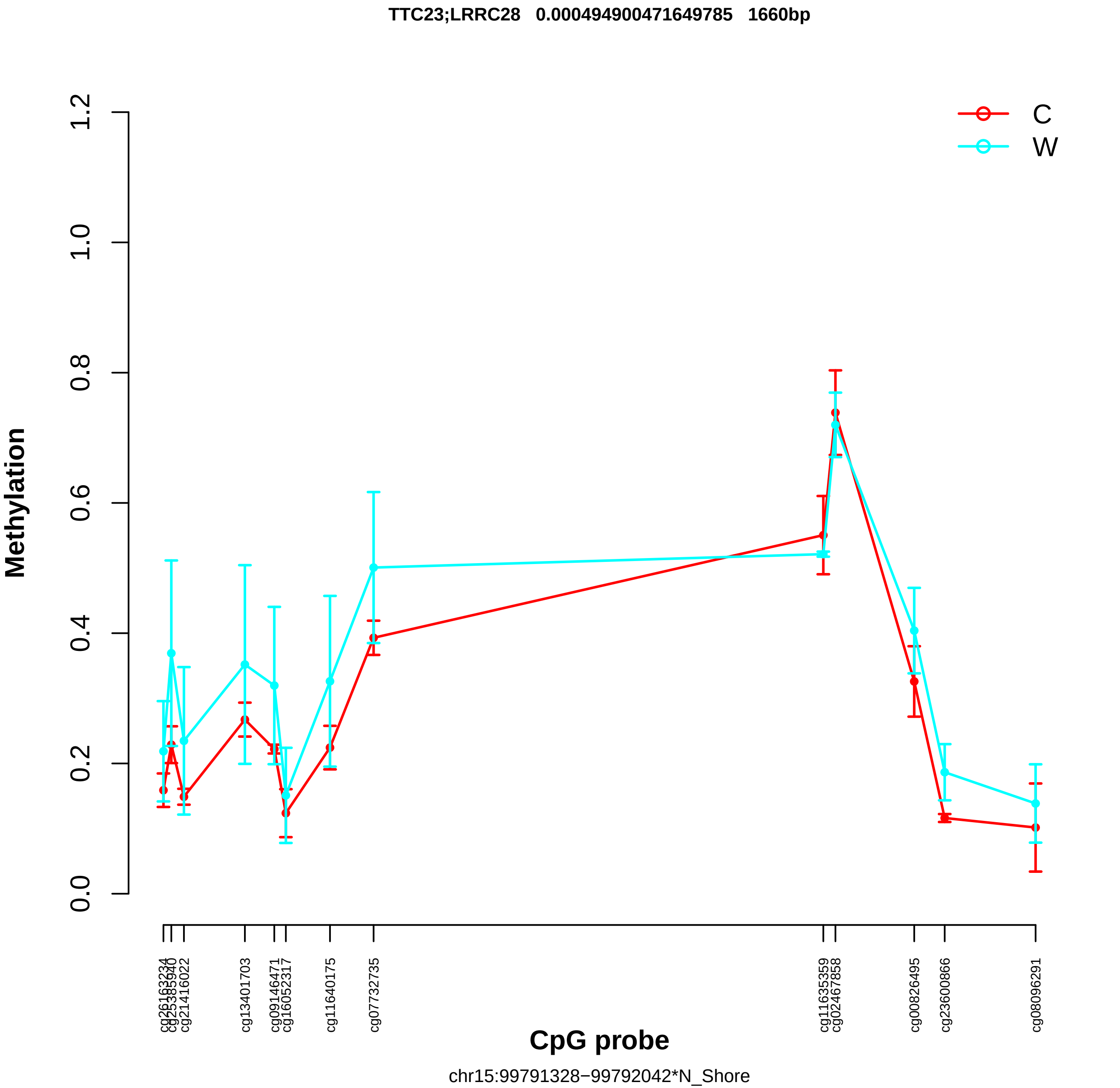
TTC23;LRRC28 0.000494900471649785 1660bp
Methylation
1.2
1.0
0.8
0.6
0.4
0.2
0.0
C
W
cg26163234
cg25385940
cg21416022
cg13401703
cg09146471
cg16052317
cg11640175
cg07732735
cg11635359
cg02467858
cg00826495
cg23600866
cg08096291
CpG probe
chr15:99791328−99792042*N_Shore

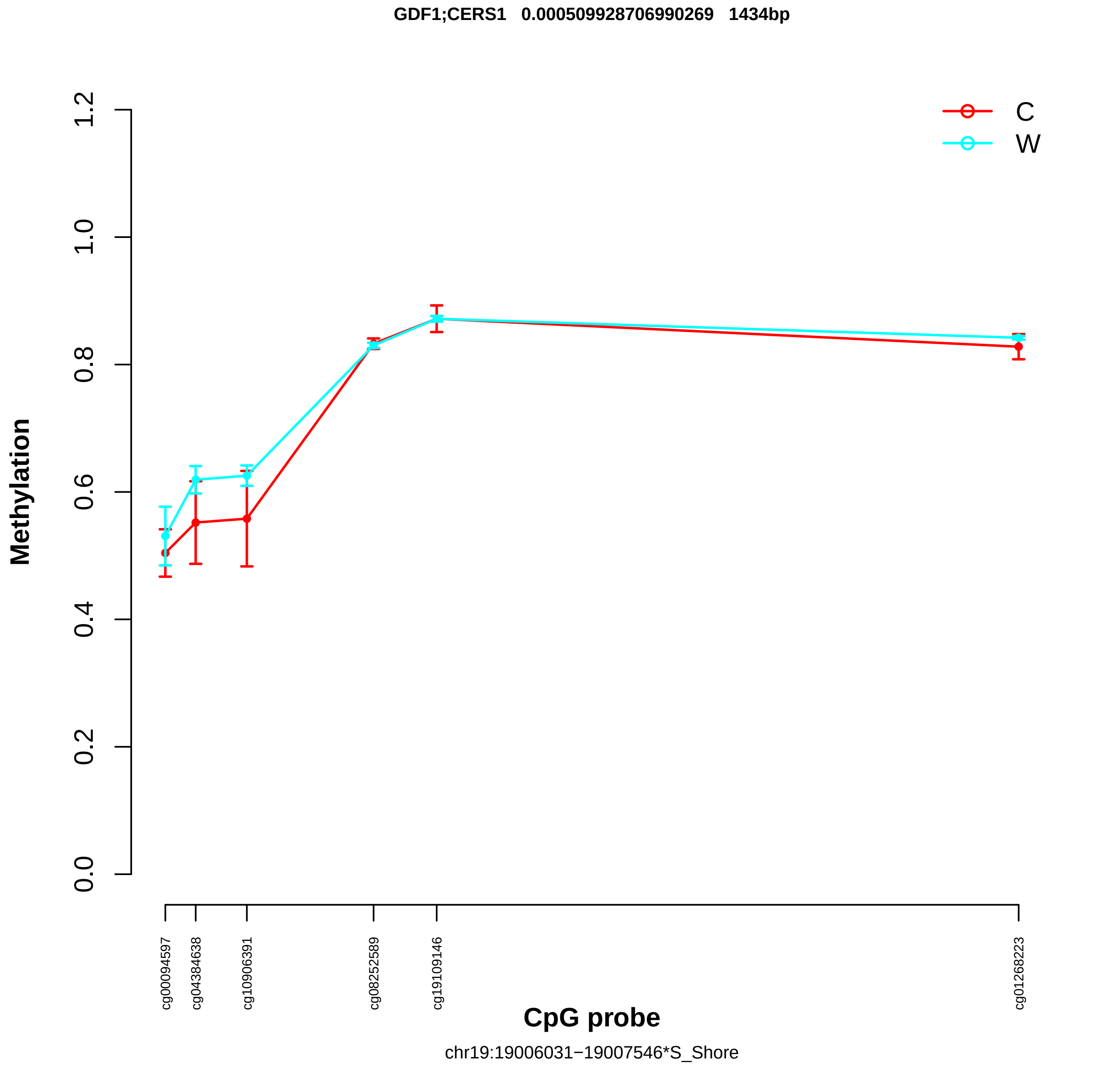

GDF1;CERS1 0.000509928706990269 1434bp
Methylation
1.2
1.0
0.8
0.6
0.4
0.2
0.0
C
W
cg00094597
cg04384638
cg10906391
cg08252589
cg19109146
cg01268223
CpG probe
chr19:19006031−19007546*S_Shore

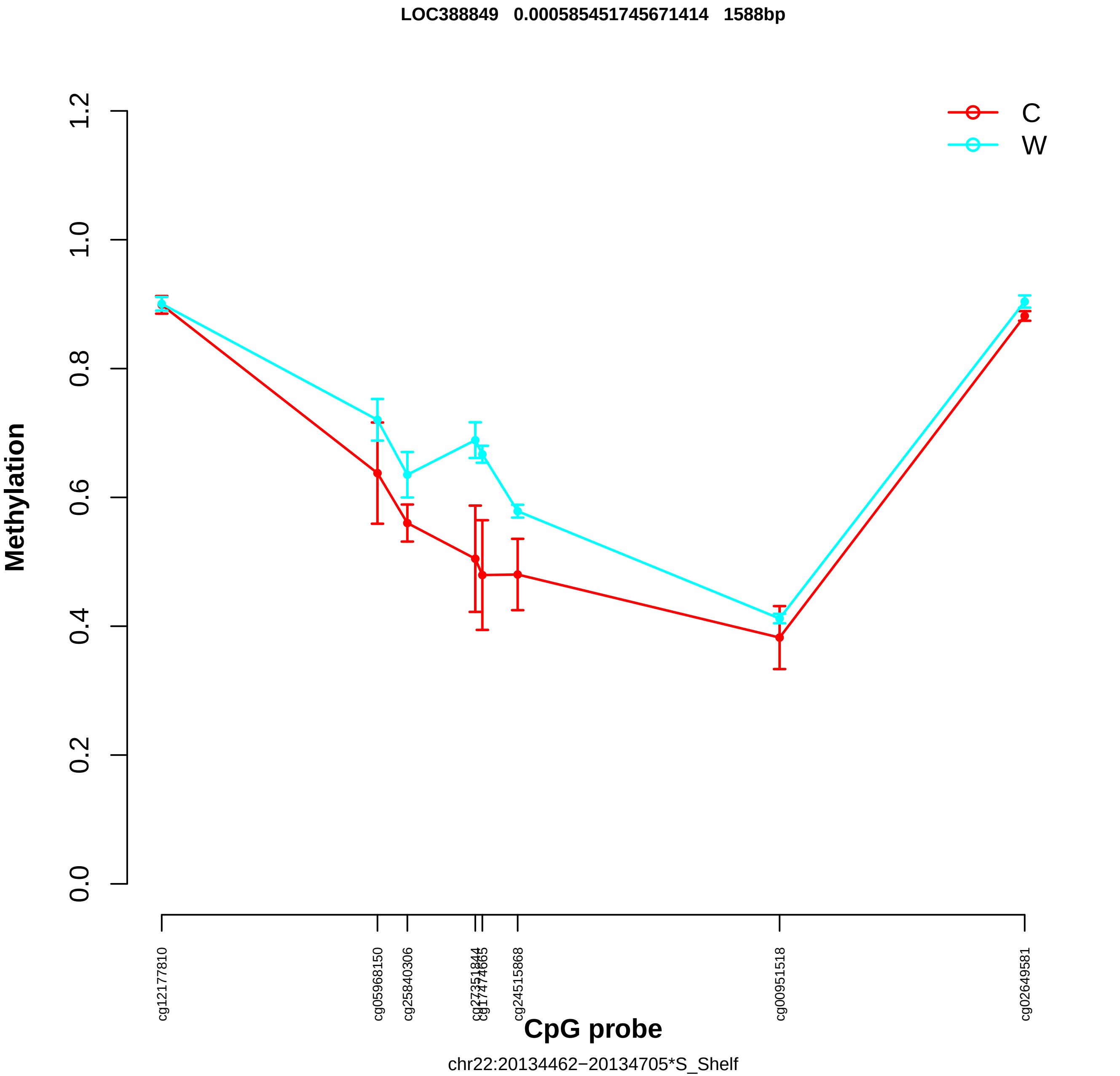

LOC388849 0.000585451745671414 1588bp
Methylation
0.0
0.2
0.4
0.6
0.8
1.0
1.2
C
W
cg12177810
cg05968150
cg25840306
cg24515868
cg00951518
cg02649581
CpG probe
chr22:20134462−20134705*S_Shelf

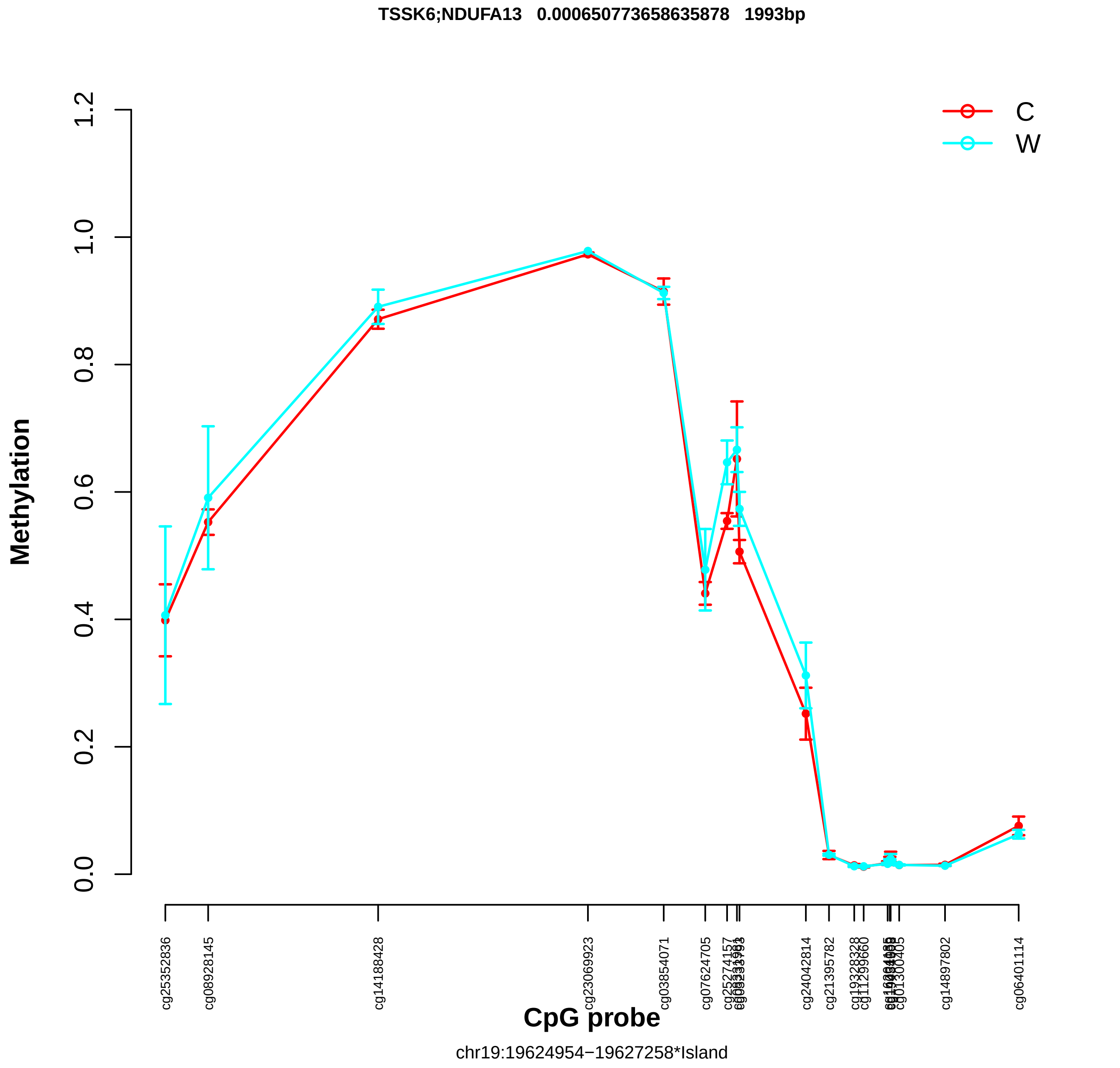

TSSK6;NDUFA13 0.000650773658635878 1993bp
Methylation
0.0
0.2
0.4
0.6
0.8
1.0
1.2
C
W
cg25352836
cg08928145
cg14188428
cg23069923
cg03854071
cg07624705
cg21395782
cg24042814
cg14897802
cg06401114
CpG probe
chr19:19624954−19627258*Island

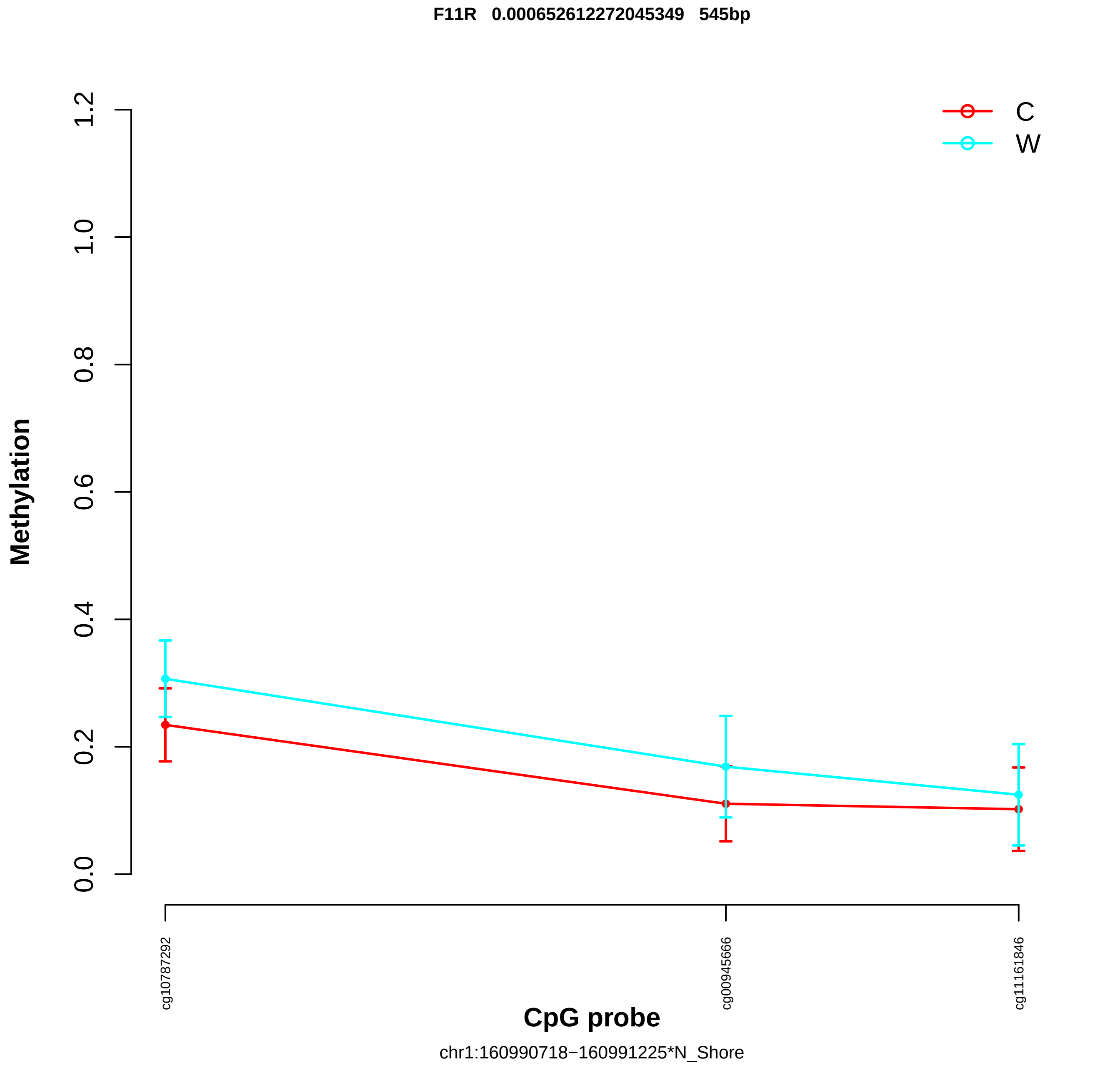
F11R 0.000652612272045349 545bp
Methylation
0.0
0.2
0.4
0.6
0.8
1.0
1.2
C
W
cg10787292
cg00945666
cg11161846
CpG probe
chr1:160990718−160991225*N_Shore

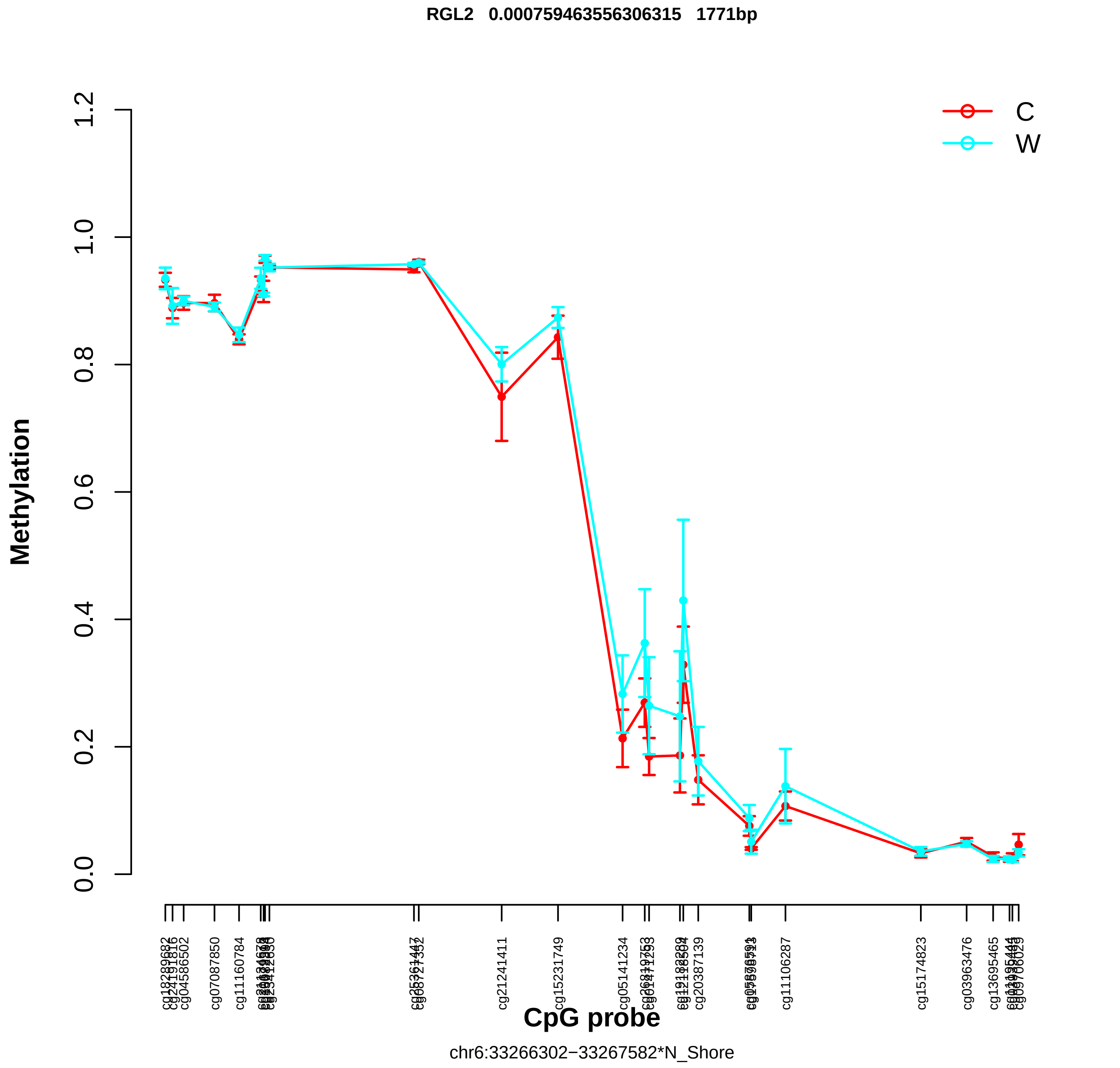
RGL2 0.000759463556306315 1771bp
Methylation
CpG probe
0.0
0.2
0.4
0.6
0.8
1.0
1.2
C
W
cg04586502
cg07087850
cg11160784
cg21241411
cg15231749
cg05141234
cg20387139
cg11106287
cg15174823
cg03963476
cg13695465
chr6:33266302−33267582*N_Shore

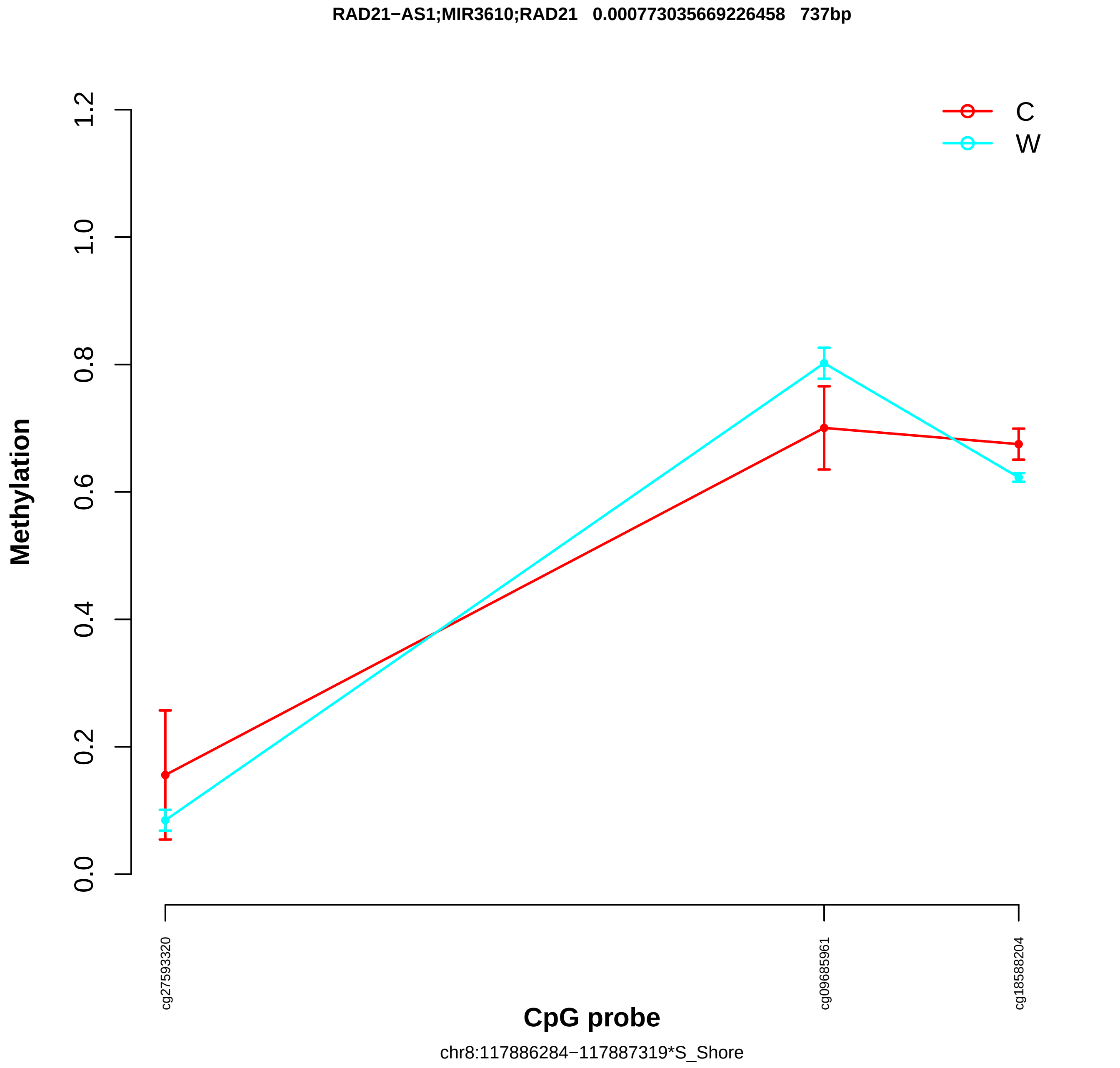

RAD21−AS1;MIR3610;RAD21 0.000773035669226458 737bp
Methylation
0.0
0.2
0.4
0.6
0.8
1.0
1.2
C
W
cg27593320
cg09685961
cg18588204
CpG probe
chr8:117886284−117887319*S_Shore

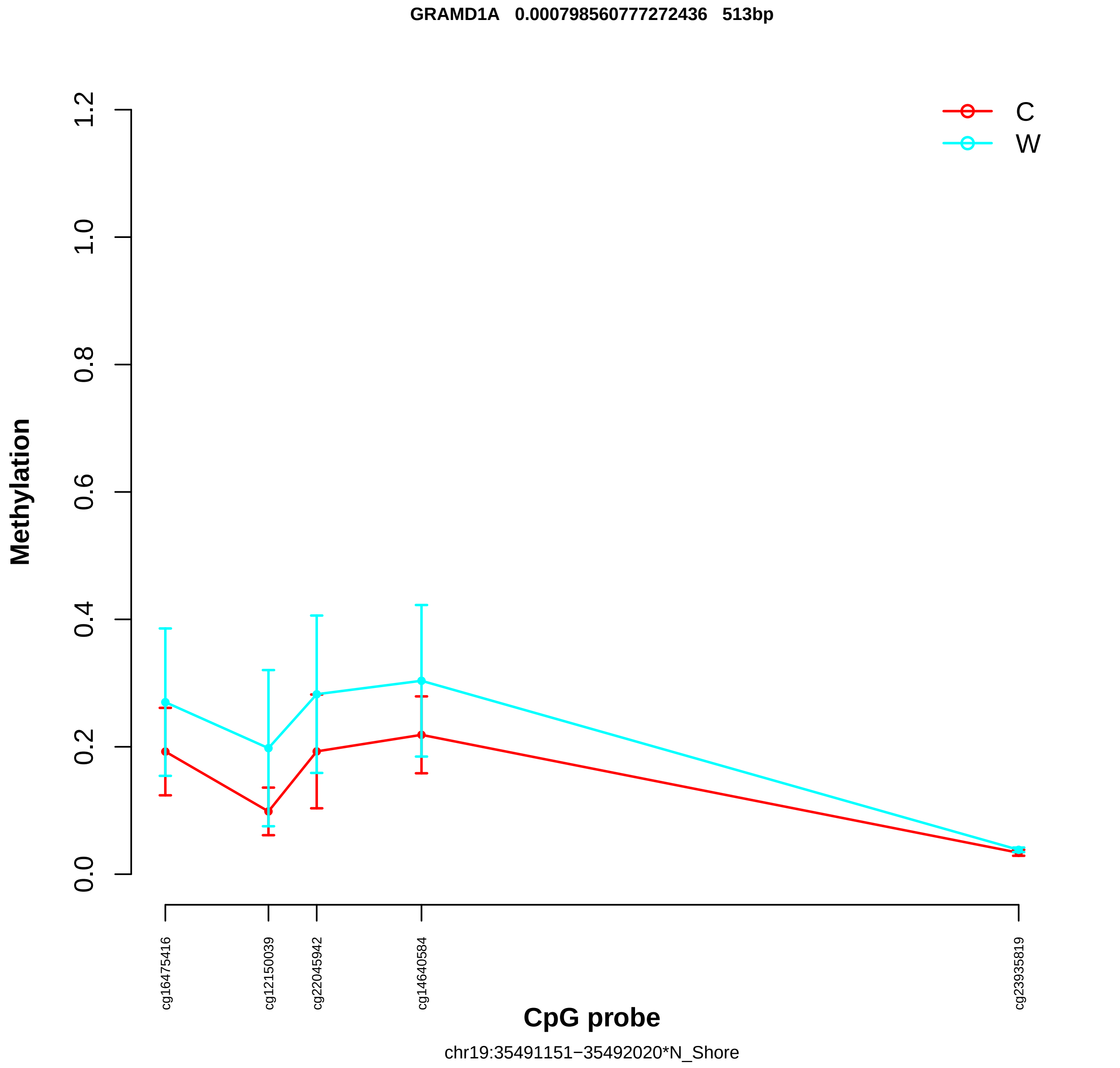
GRAMD1A 0.000798560777272436 513bp
Methylation
1.2
1.0
0.8
0.6
0.4
0.2
0.0
C
W
cg16475416
cg12150039
cg22045942
cg14640584
cg23935819
CpG probe
chr19:35491151−35492020*N_Shore

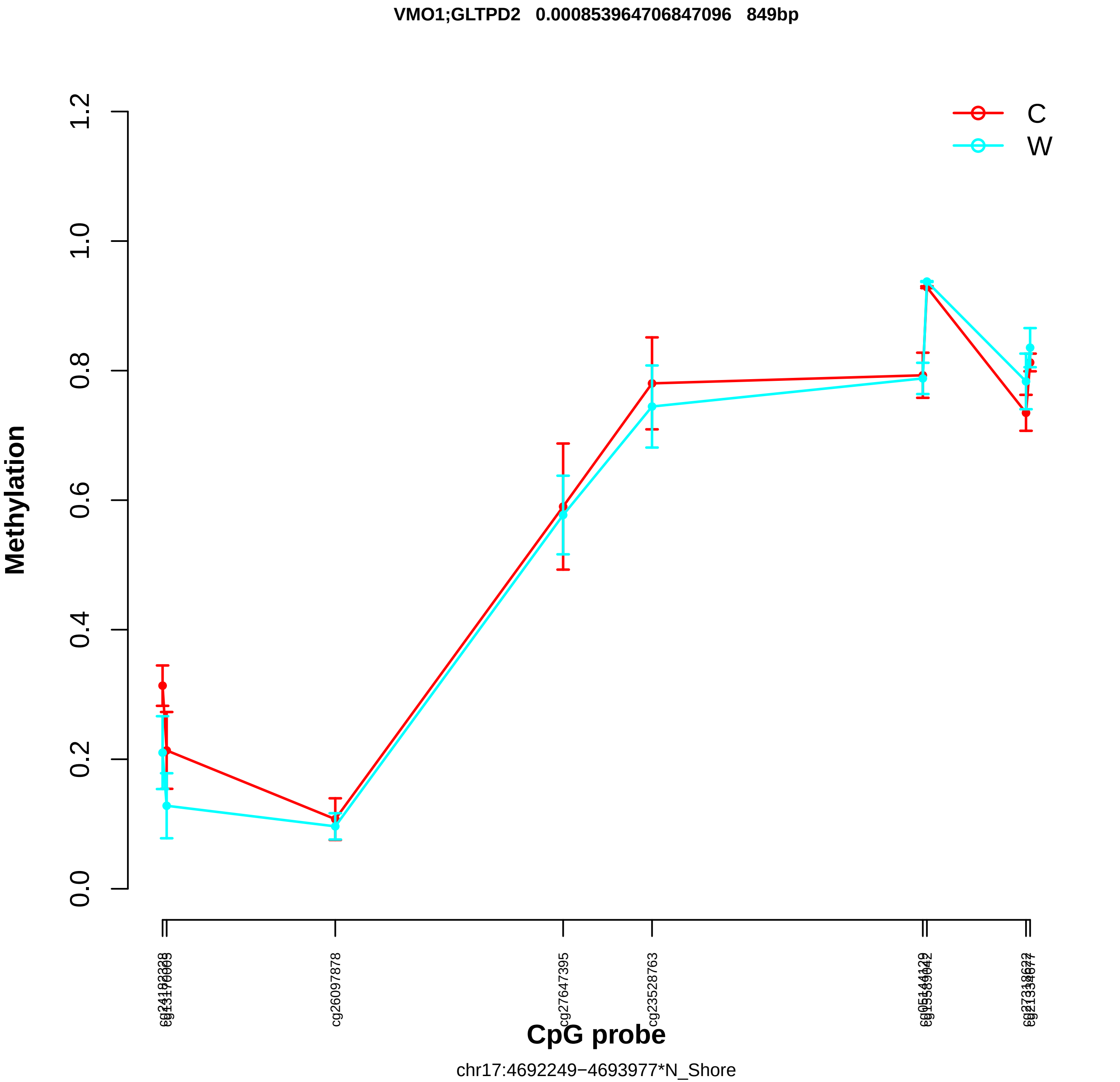
VMO1;GLTPD2 0.000853964706847096 849bp
C
W
Methylation
0.0
0.2
0.4
0.6
0.8
1.0
1.2
cg26097878
cg27647395
cg23528763
CpG probe
chr17:4692249−4693977*N_Shore

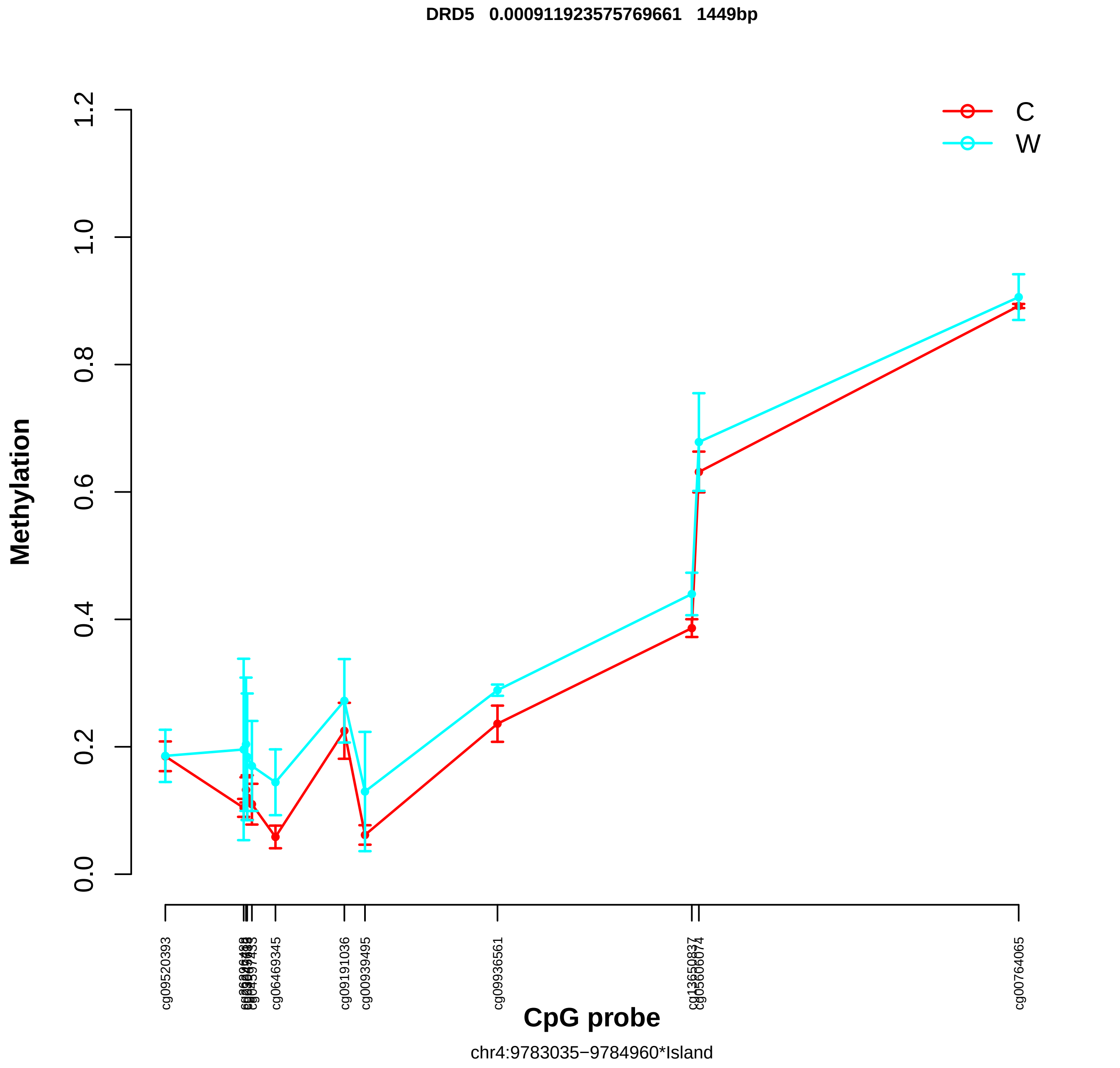
DRD5 0.000911923575769661 1449bp
Methylation
0.0
0.2
0.4
0.6
0.8
1.0
1.2
C
W
cg09520393
cg06469345
cg09191036
cg00939495
cg09936561
cg00764065
CpG probe
chr4:9783035−9784960*Island

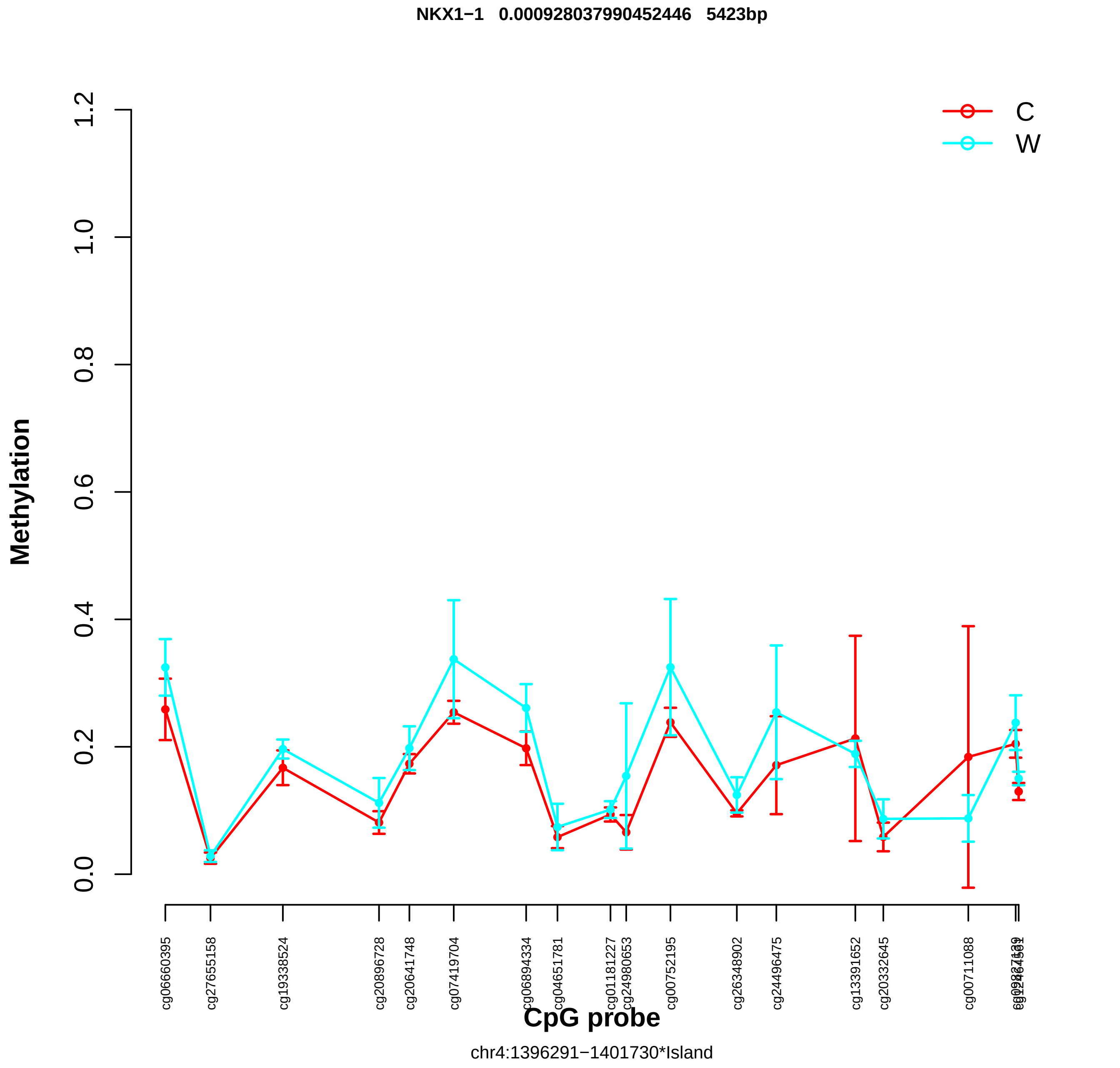
NKX1−1 0.000928037990452446 5423bp
Methylation
0.0
0.2
0.4
0.6
0.8
1.0
1.2
C
W
cg06660395
cg27655158
cg19338524
cg20896728
cg20641748
cg07419704
cg06894334
cg04651781
cg01181227
cg24980653
cg00752195
cg26348902
cg24496475
cg13391652
cg20332645
cg00711088
CpG probe
chr4:1396291−1401730*Island
