# Supplementary material for: Genome-wide DNA methylation analysis in blood cells from patients with Werner syndrome
Source: Clin Epigenetics. 2017 Aug 30;9:92. doi: 10.1186/s13148-017-0389-4 (PMC5577832; doi:10.1186/s13148-017-0389-4)
Supplement: Supplementary file 5 — Boxplots of DNA methylation values of the CpG probes in the genes belonging to the enriched pathways in naïve B cells, CTR LCL, WS LCL from the GSE42865 dataset and in CTR and WS whole blood samples assessed in this study. The brown dots correspond to the individual samples. For each CpG probe, the title of the plot reports the name of the CpG probe, the name of the gene/genes in which the probe maps and, if present, the name of the CpG island and the position of the probe respect to the CpG island. (PDF 41 kb) [file 13148_2017_389_MOESM5_ESM.pdf]

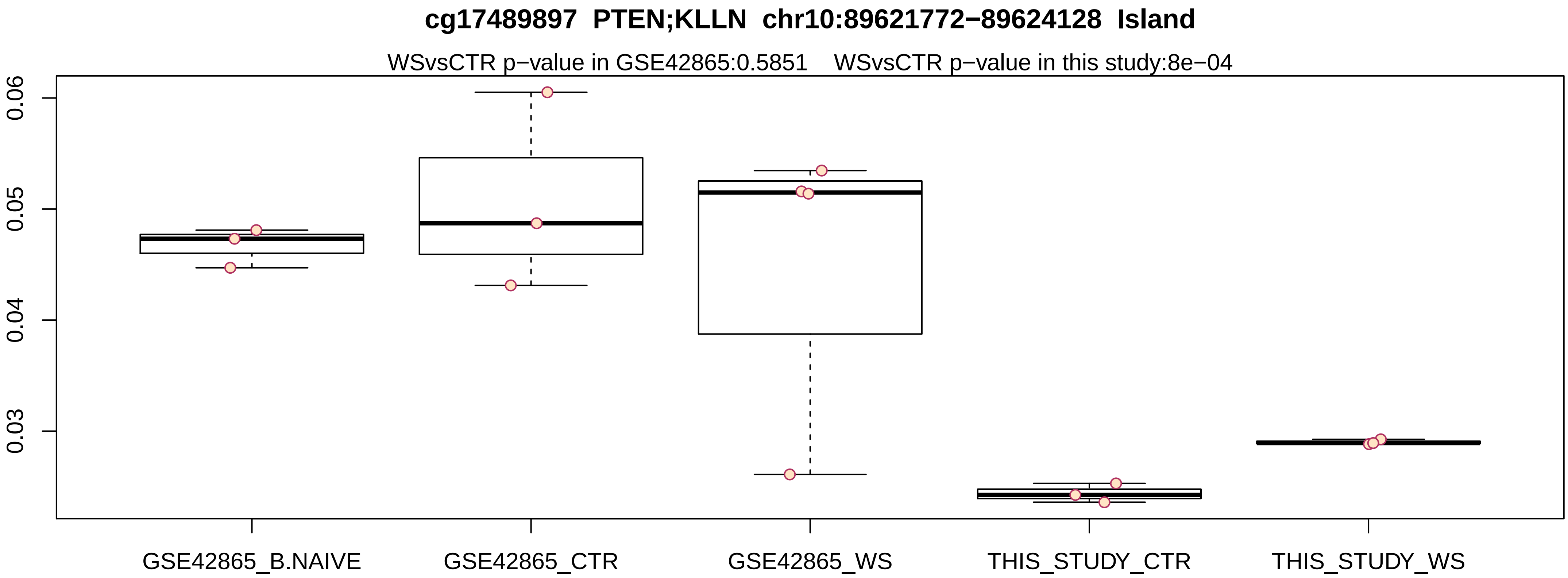
cg17489897 PTEN;KLLN chr10:89621772−89624128 Island
WSvsCTR p−value in GSE42865:0.5851 WSvsCTR p−value in this study:8e−04
0.06
0.05
0.04
0.03
GSE42865_B.NAIVE
GSE42865_CTR
GSE42865_WS
THIS_STUDY_CTR
THIS_STUDY_WS

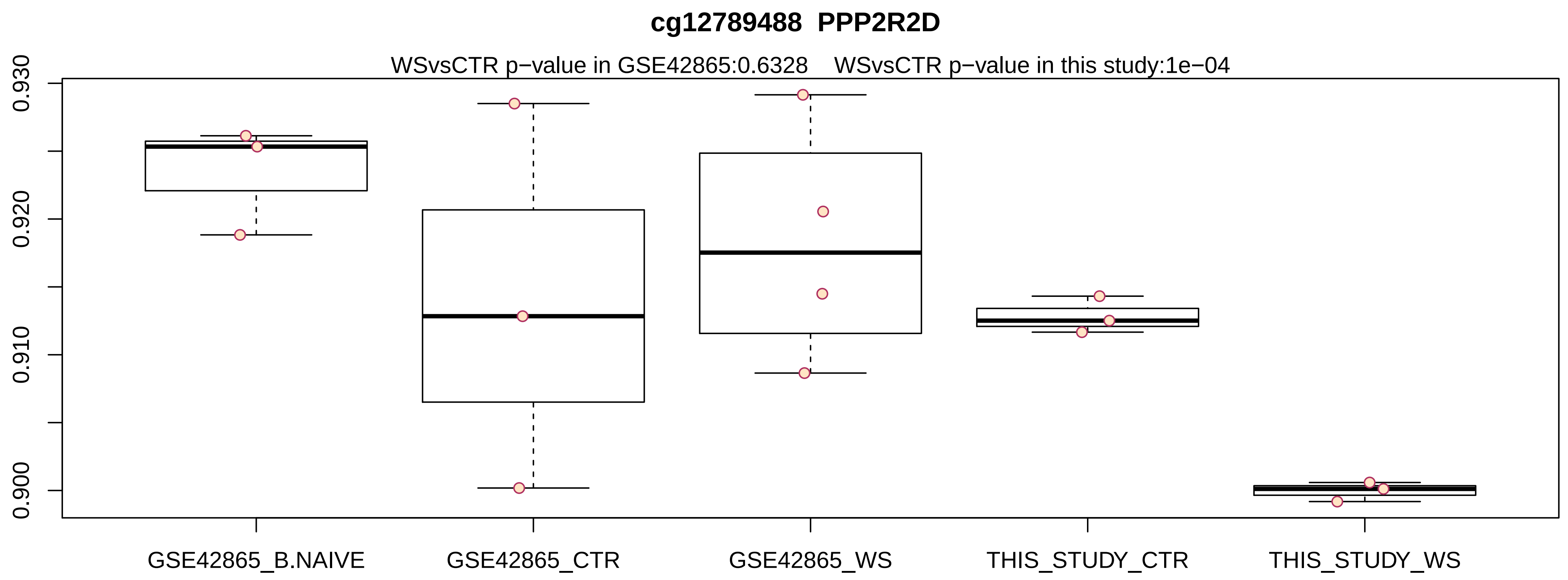
cg12789488 PPP2R2D
WSvsCTR p−value in GSE42865:0.6328 WSvsCTR p−value in this study:1e−04
0.900
0.910
0.920
0.930
GSE42865_B.NAIVE
GSE42865_CTR
GSE42865_WS
THIS_STUDY_CTR
THIS_STUDY_WS

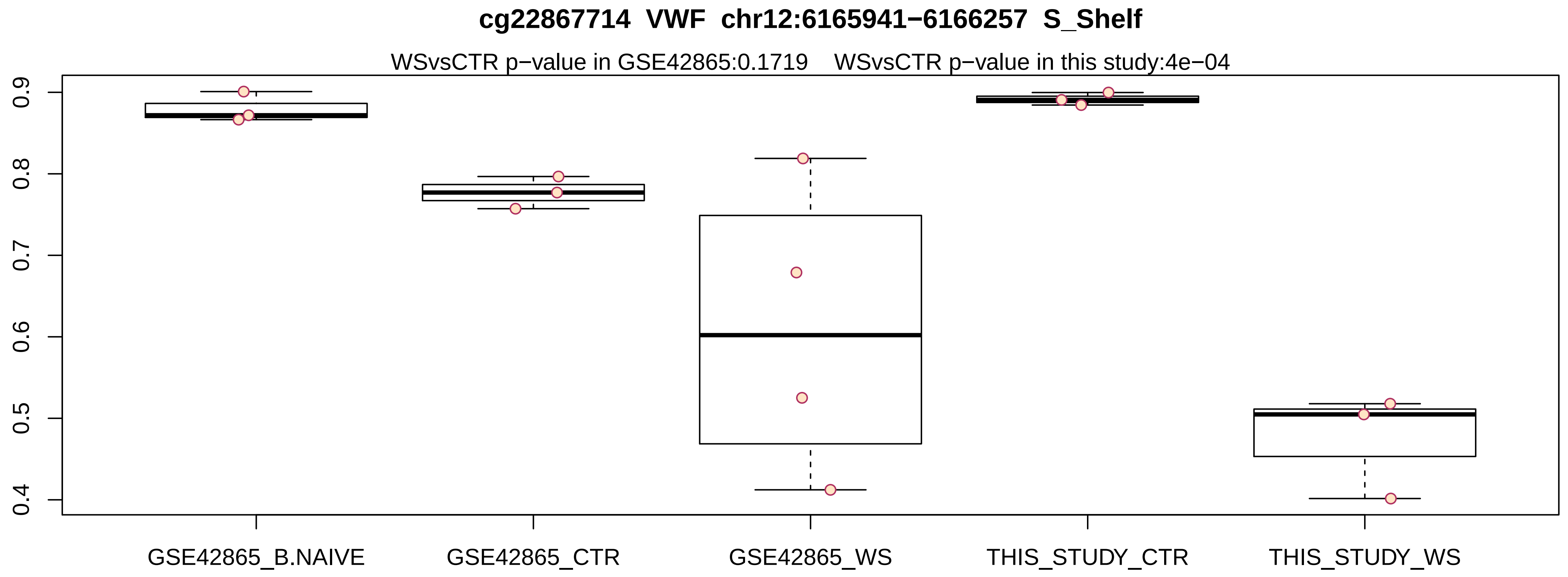
cg22867714 VWF chr12:6165941−6166257 S_Shelf
WSvsCTR p−value in GSE42865:0.1719 WSvsCTR p−value in this study:4e−04
0.4
0.5
0.6
0.7
0.8
0.9
GSE42865_B.NAIVE
GSE42865_CTR
GSE42865_WS
THIS_STUDY_CTR
THIS_STUDY_WS

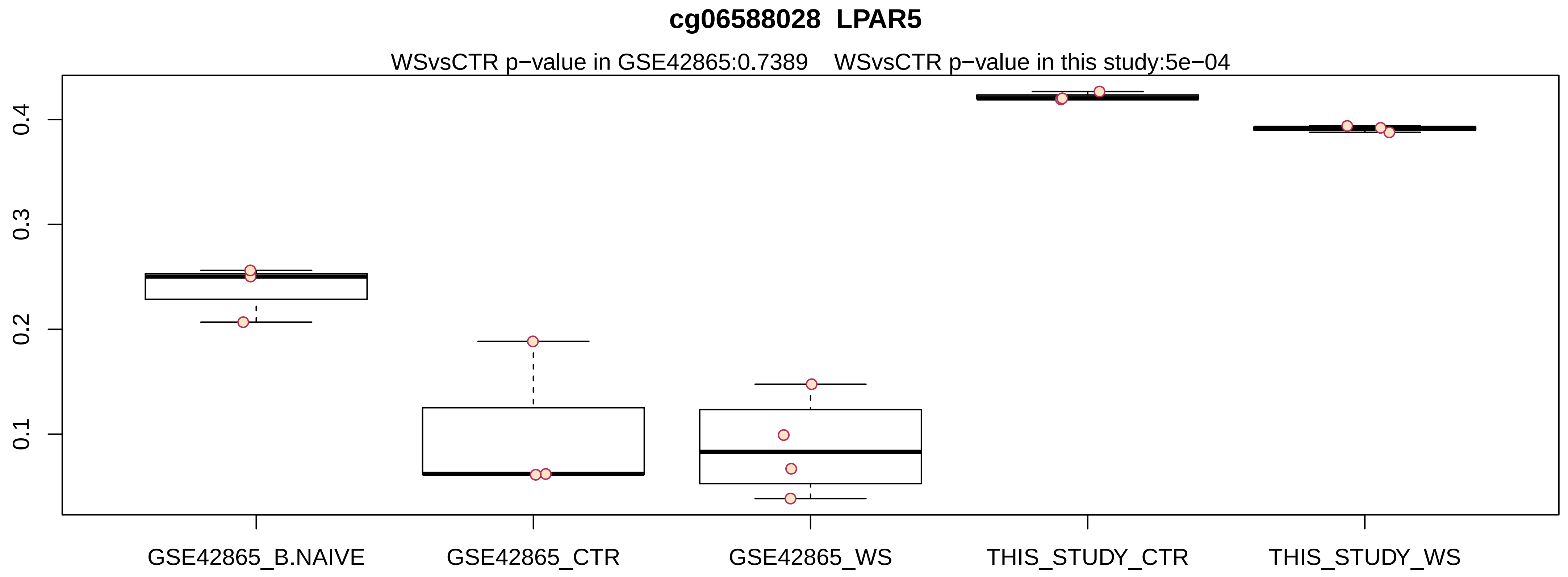
cg06588028 LPAR5
WSvsCTR p−value in GSE42865:0.7389 WSvsCTR p−value in this study:5e−04
0.1
0.2
0.3
0.4
GSE42865_B.NAIVE
GSE42865_CTR
GSE42865_WS
THIS_STUDY_CTR
THIS_STUDY_WS

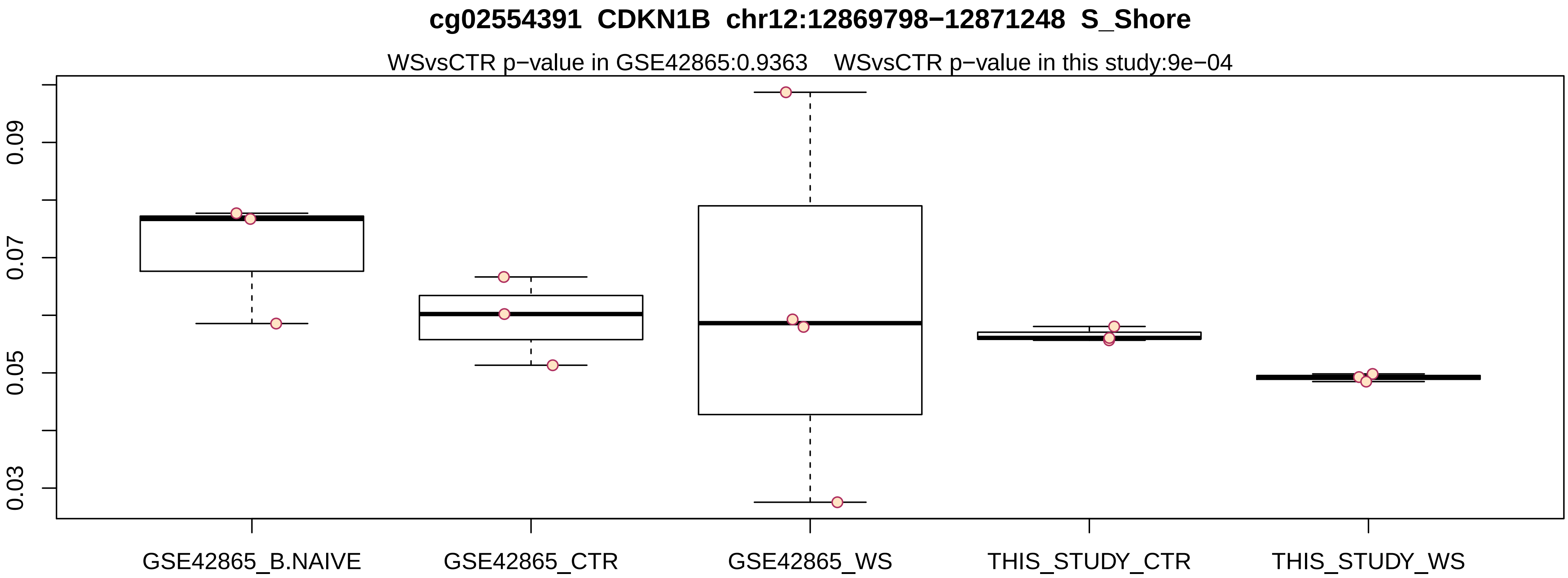
cg02554391 CDKN1B chr12:12869798−12871248 S_Shore
WSvsCTR p−value in GSE42865:0.9363 WSvsCTR p−value in this study:9e−04
0.03
0.05
0.07
0.09
GSE42865_B.NAIVE
GSE42865_CTR
GSE42865_WS
THIS_STUDY_CTR
THIS_STUDY_WS

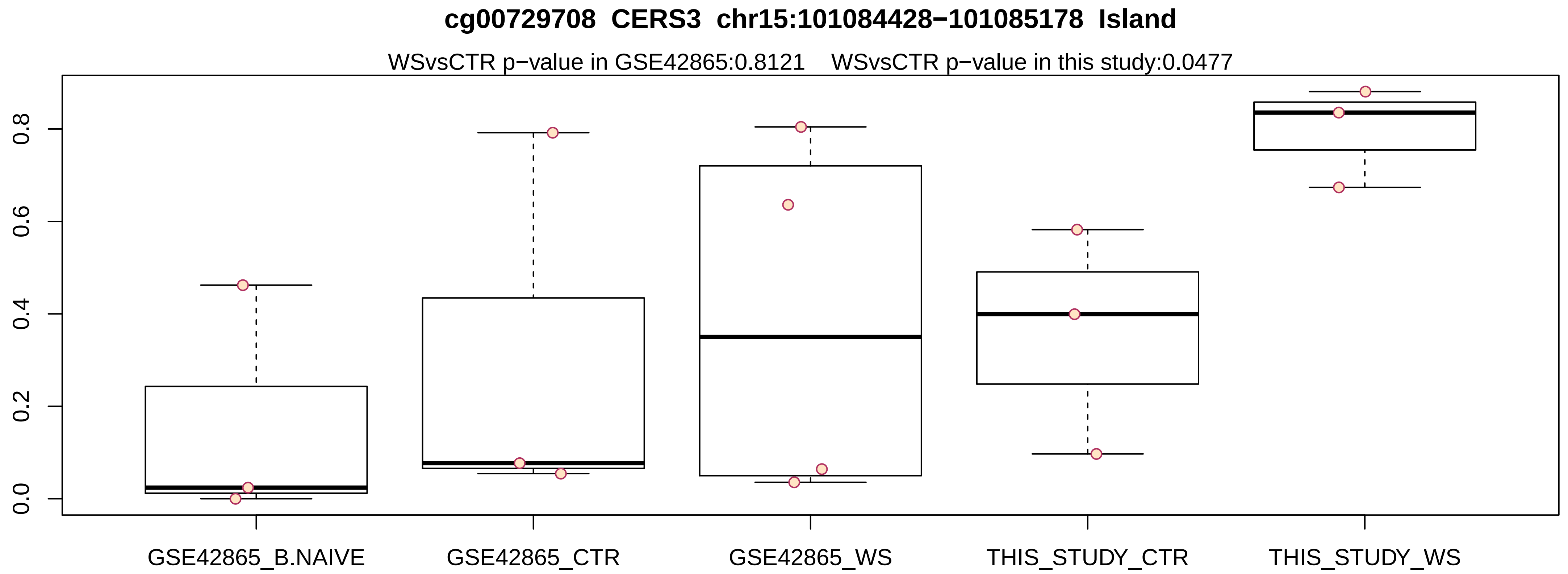
cg00729708 CERS3 chr15:101084428−101085178 Island
WSvsCTR p−value in GSE42865:0.8121 WSvsCTR p−value in this study:0.0477
0.0
0.2
0.4
0.6
0.8
GSE42865_B.NAIVE
GSE42865_CTR
GSE42865_WS
THIS_STUDY_CTR
THIS_STUDY_WS

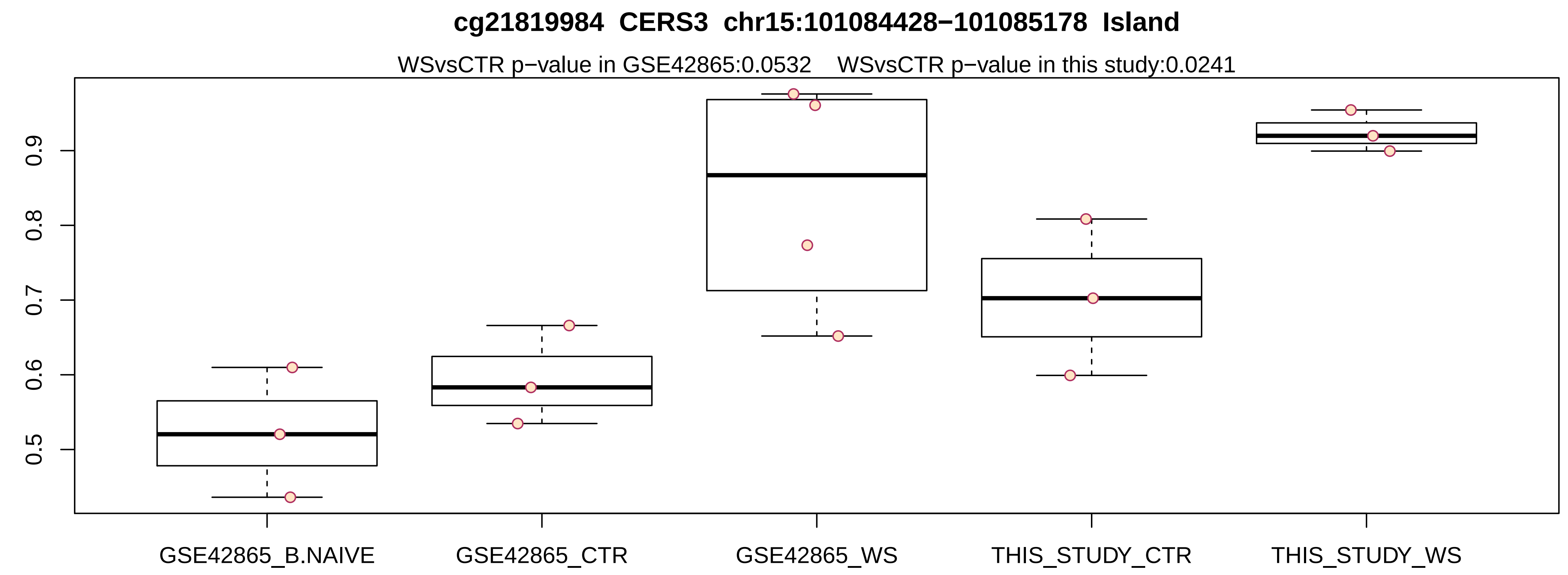
cg21819984 CERS3 chr15:101084428−101085178 Island
WSvsCTR p−value in GSE42865:0.0532 WSvsCTR p−value in this study:0.0241
0.5
0.6
0.7
0.8
0.9
GSE42865_B.NAIVE
GSE42865_CTR
GSE42865_WS
THIS_STUDY_CTR
THIS_STUDY_WS

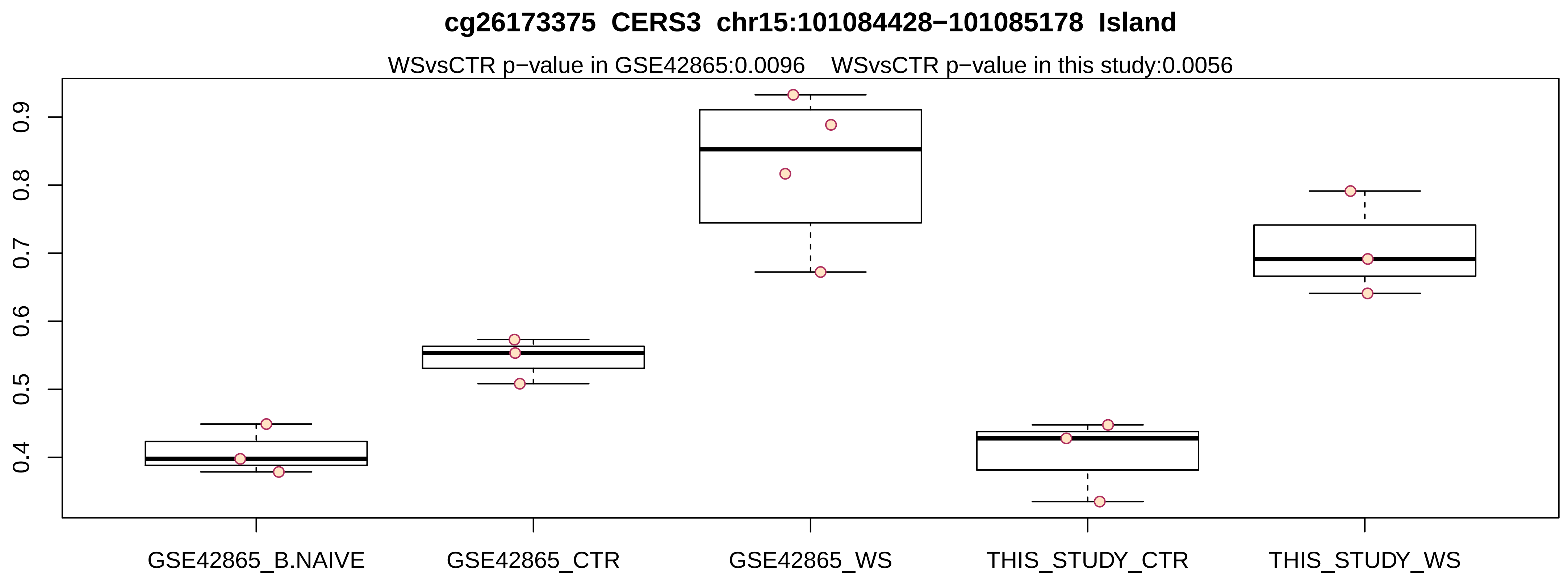
cg26173375 CERS3 chr15:101084428−101085178 Island
WSvsCTR p−value in GSE42865:0.0096 WSvsCTR p−value in this study:0.0056
0.4
0.5
0.6
0.7
0.8
0.9
GSE42865_B.NAIVE
GSE42865_CTR
GSE42865_WS
THIS_STUDY_CTR
THIS_STUDY_WS

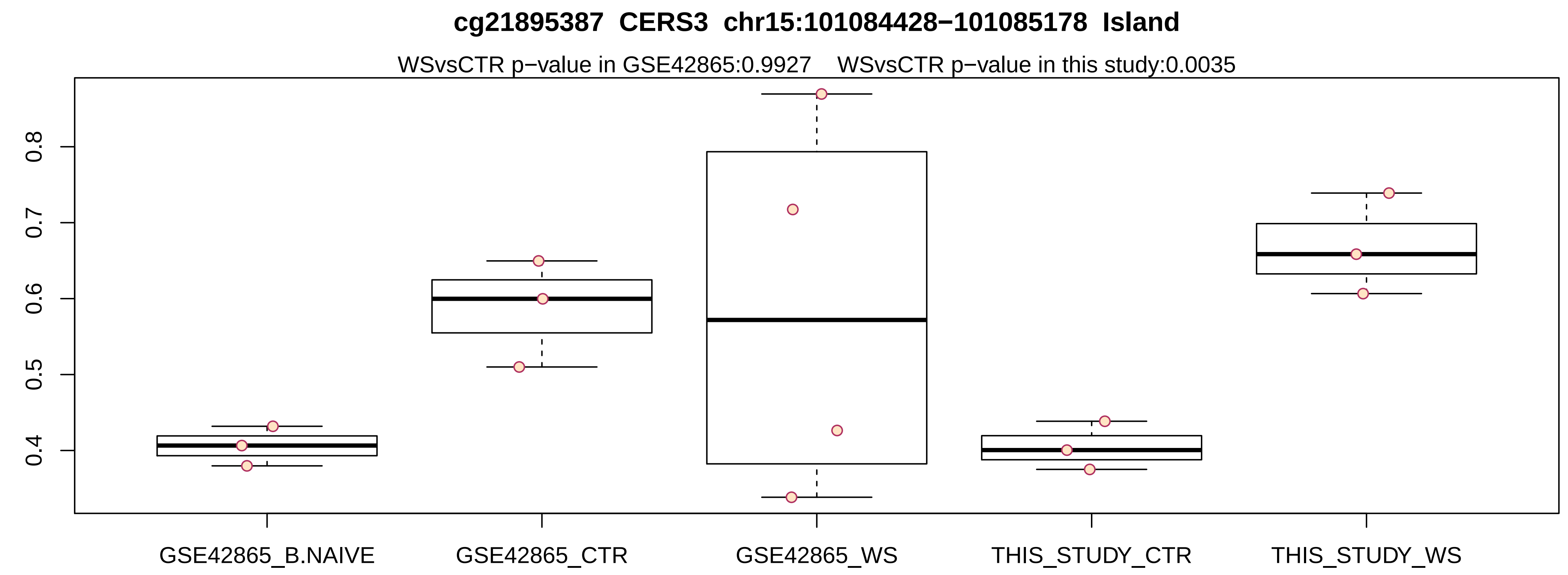
cg21895387 CERS3 chr15:101084428−101085178 Island
WSvsCTR p−value in GSE42865:0.9927 WSvsCTR p−value in this study:0.0035
0.4
0.5
0.6
0.7
0.8
GSE42865_B.NAIVE
GSE42865_CTR
GSE42865_WS
THIS_STUDY_CTR
THIS_STUDY_WS

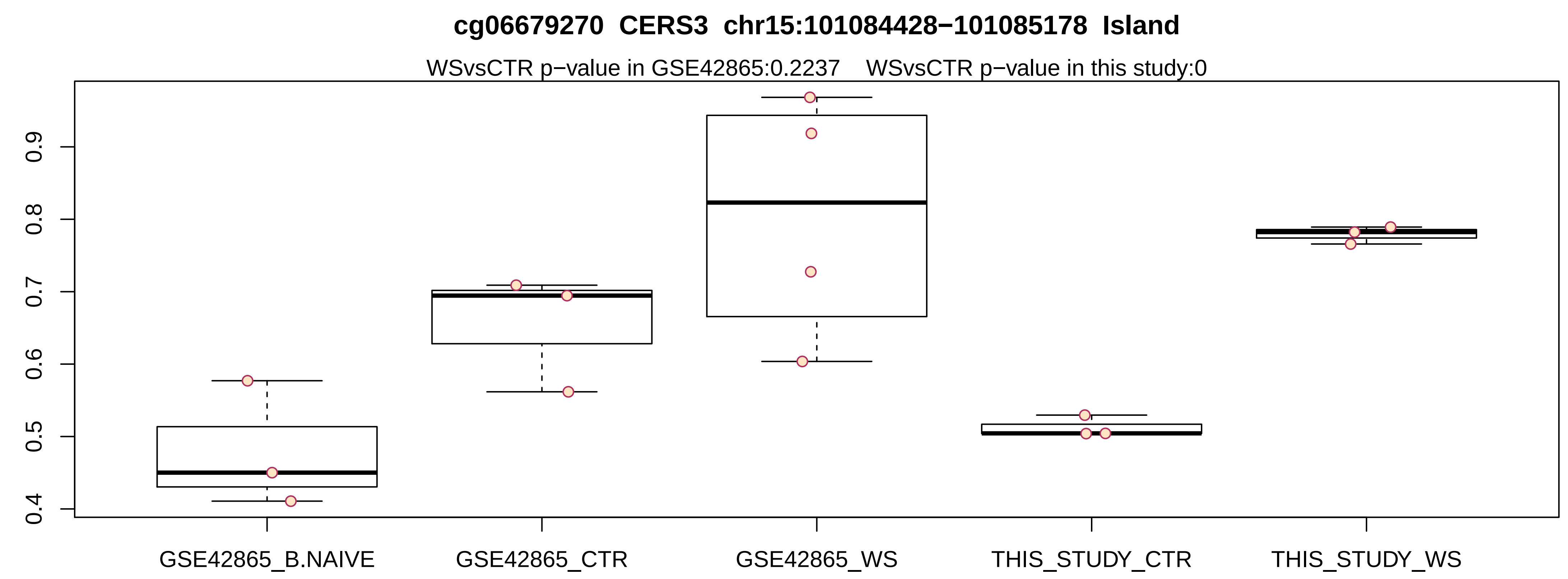
cg06679270 CERS3 chr15:101084428−101085178 Island
WSvsCTR p−value in GSE42865:0.2237 WSvsCTR p−value in this study:0
0.4
0.5
0.6
0.7
0.8
0.9
GSE42865_B.NAIVE
GSE42865_CTR
GSE42865_WS
THIS_STUDY_CTR
THIS_STUDY_WS

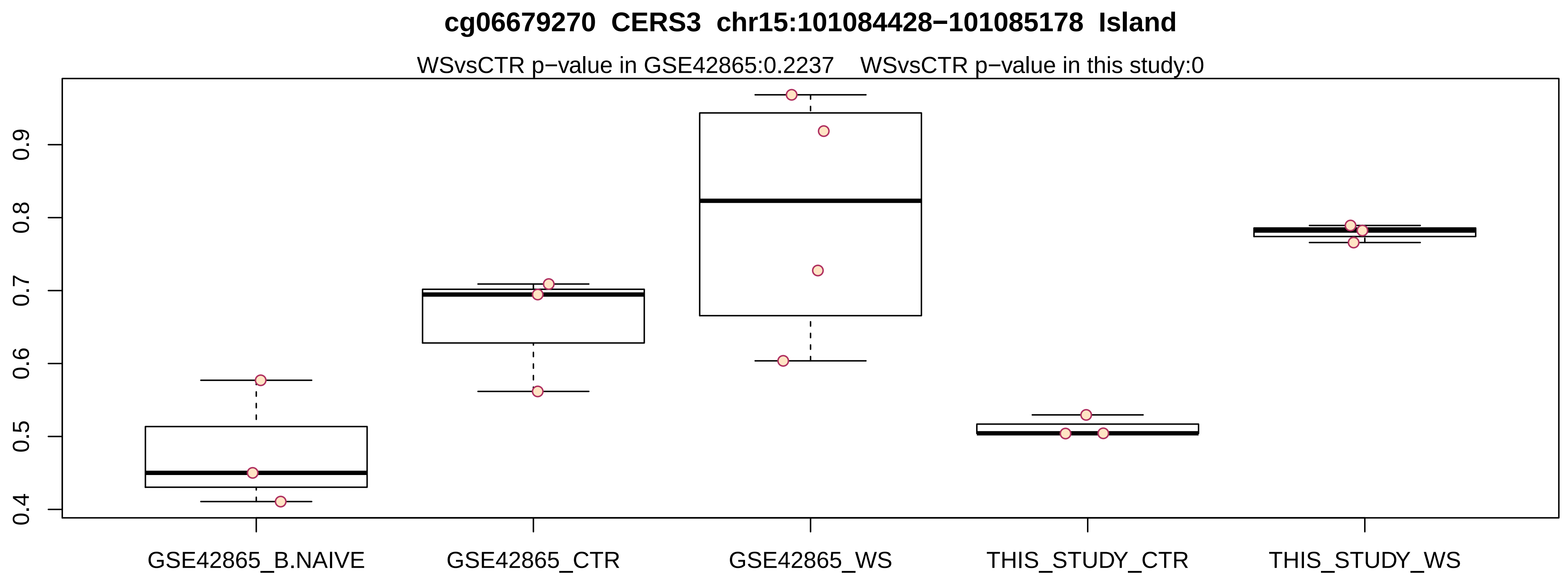

cg06679270 CERS3 chr15:101084428−101085178 Island
WSvsCTR p−value in GSE42865:0.2237 WSvsCTR p−value in this study:0
0.4
0.5
0.6
0.7
0.8
0.9
GSE42865_B.NAIVE
GSE42865_CTR
GSE42865_WS
THIS_STUDY_CTR
THIS_STUDY_WS

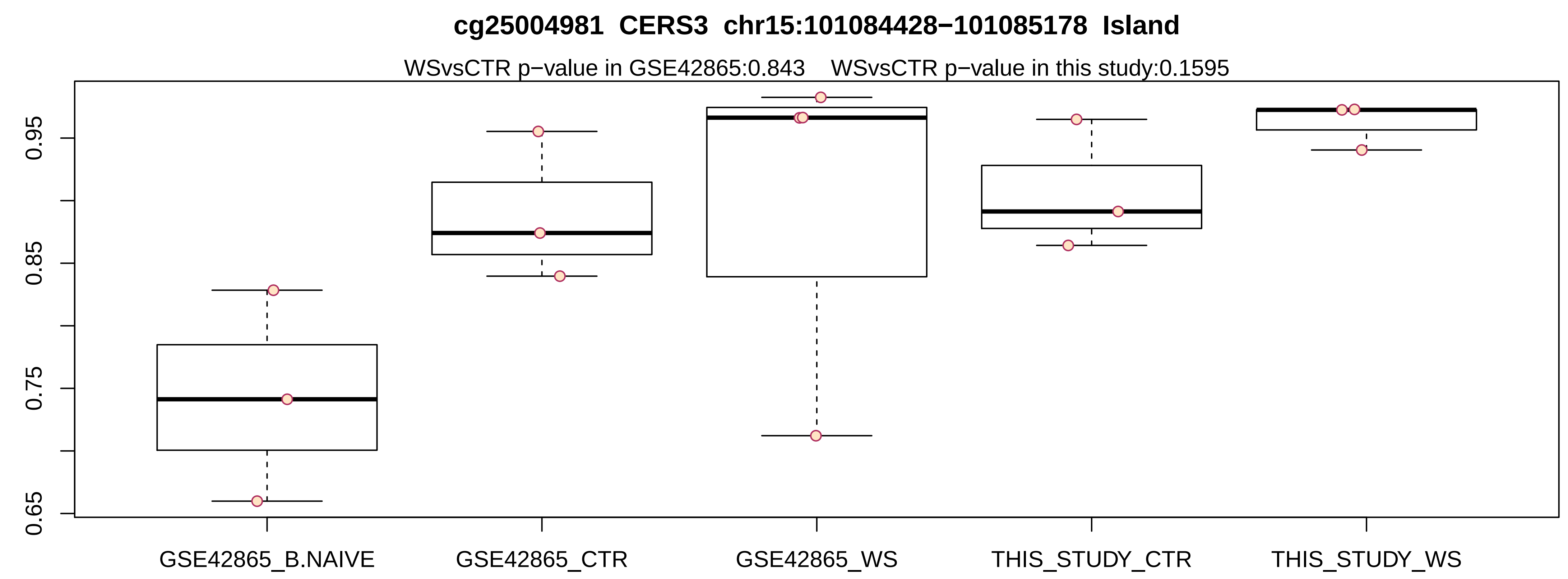

cg25004981 CERS3 chr15:101084428−101085178 Island
WSvsCTR p−value in GSE42865:0.843 WSvsCTR p−value in this study:0.1595
0.65
0.75
0.85
0.95
GSE42865_B.NAIVE
GSE42865_CTR
GSE42865_WS
THIS_STUDY_CTR
THIS_STUDY_WS

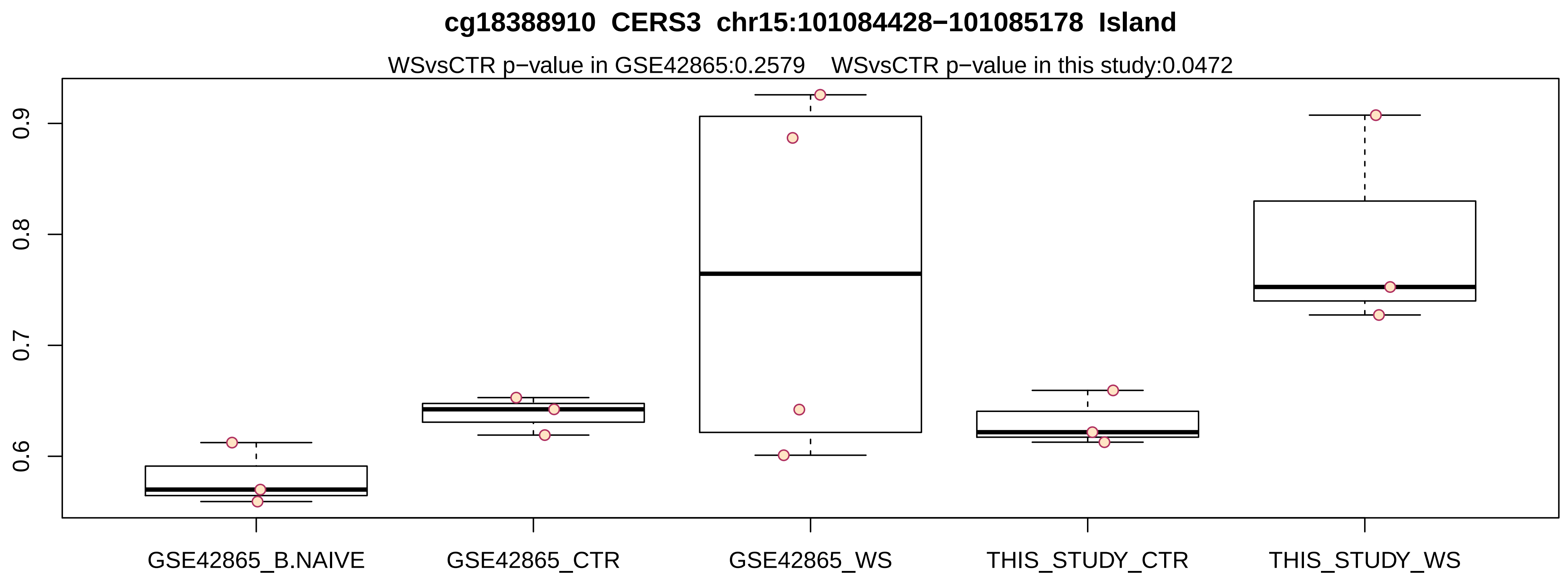
cg18388910 CERS3 chr15:101084428−101085178 Island
WSvsCTR p−value in GSE42865:0.2579 WSvsCTR p−value in this study:0.0472
0.6
0.7
0.8
0.9
GSE42865_B.NAIVE
GSE42865_CTR
GSE42865_WS
THIS_STUDY_CTR
THIS_STUDY_WS

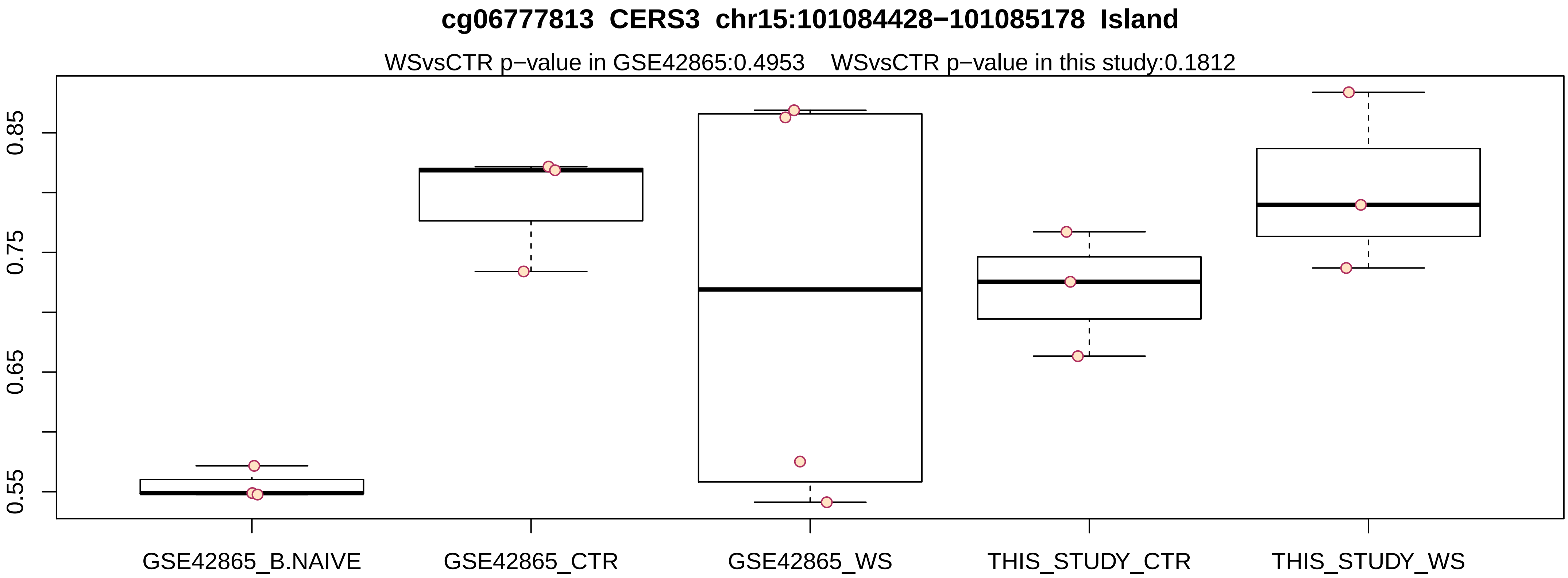

cg06777813 CERS3 chr15:101084428−101085178 Island
WSvsCTR p−value in GSE42865:0.4953 WSvsCTR p−value in this study:0.1812
0.55
0.65
0.75
0.85
GSE42865_B.NAIVE
GSE42865_CTR
GSE42865_WS
THIS_STUDY_CTR
THIS_STUDY_WS

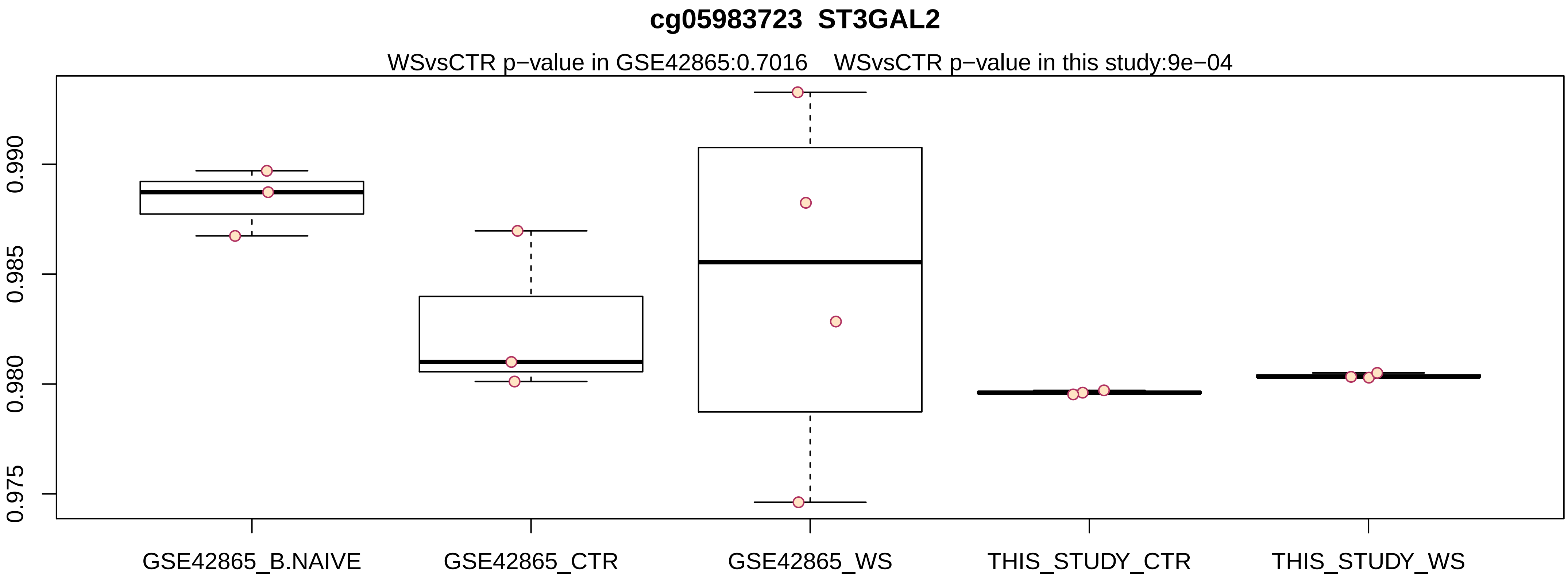
cg05983723 ST3GAL2
WSvsCTR p−value in GSE42865:0.7016 WSvsCTR p−value in this study:9e−04
0.975
0.980
0.985
0.990
GSE42865_B.NAIVE
GSE42865_CTR
GSE42865_WS
THIS_STUDY_CTR
THIS_STUDY_WS

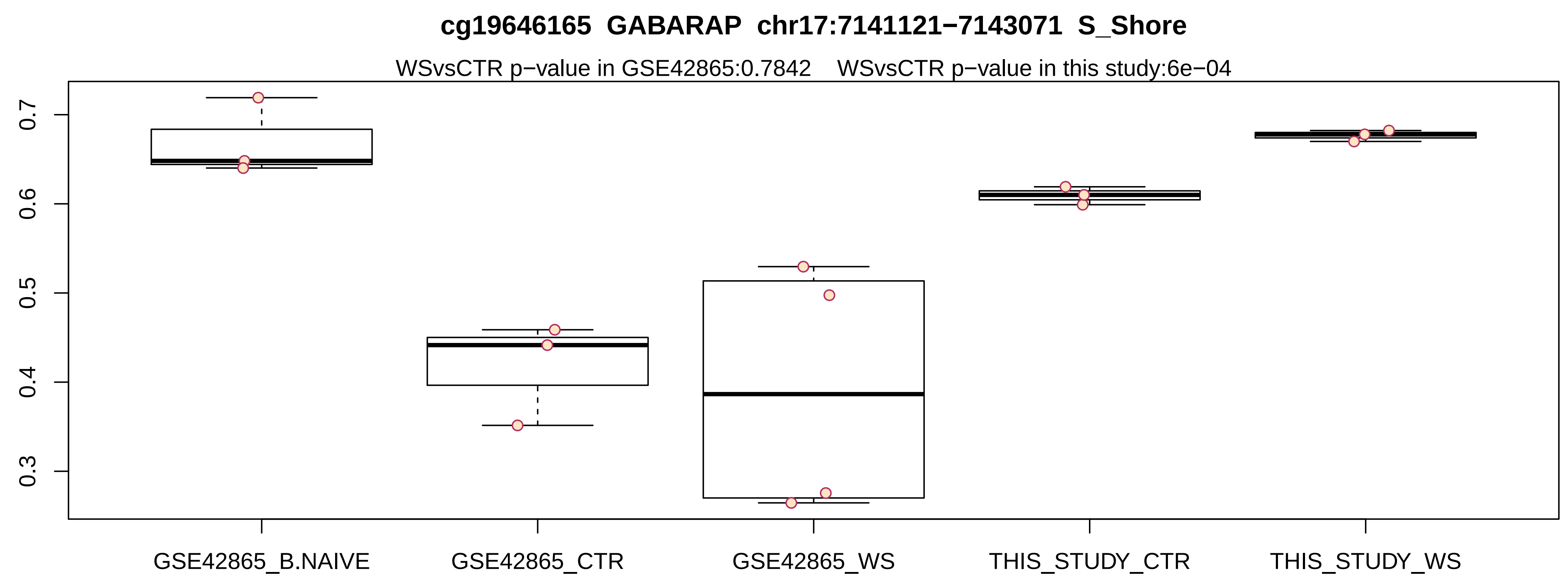
cg19646165 GABARAP chr17:7141121−7143071 S_Shore
WSvsCTR p−value in GSE42865:0.7842 WSvsCTR p−value in this study:6e−04
0.3
0.4
0.5
0.6
0.7
GSE42865_B.NAIVE
GSE42865_CTR
GSE42865_WS
THIS_STUDY_CTR
THIS_STUDY_WS

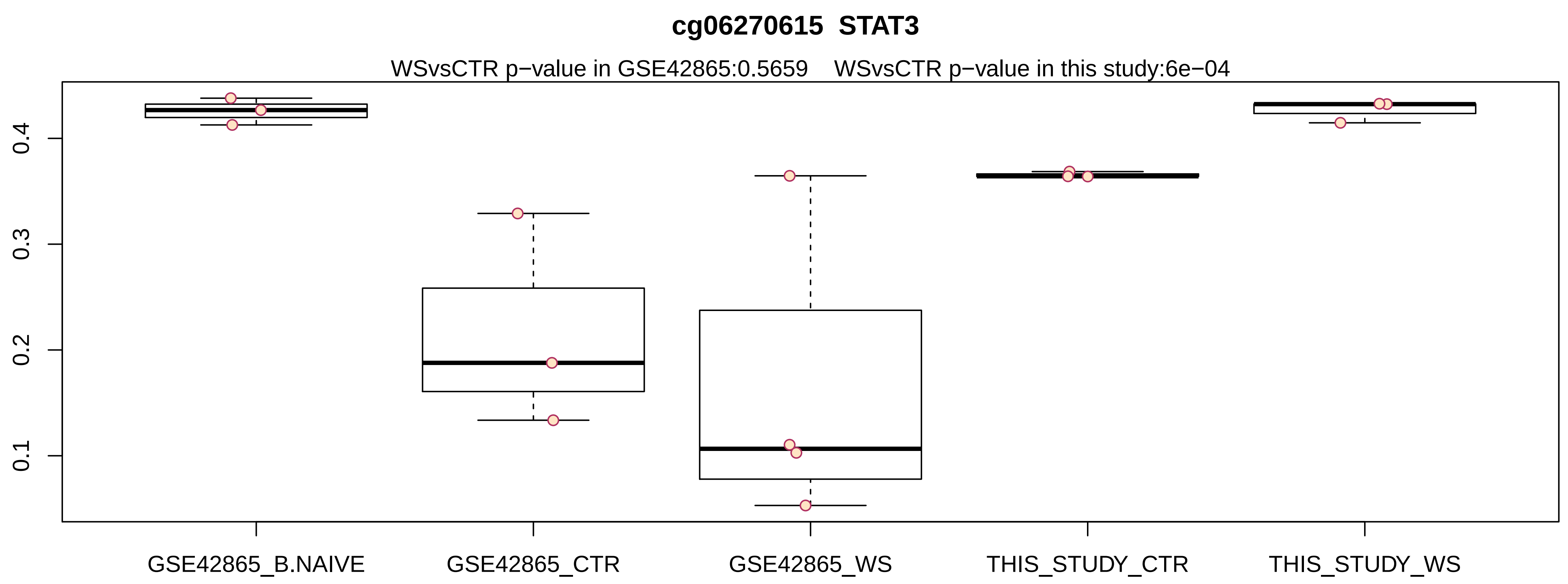
cg06270615 STAT3
WSvsCTR p−value in GSE42865:0.5659 WSvsCTR p−value in this study:6e−04
0.1
0.2
0.3
0.4
GSE42865_B.NAIVE
GSE42865_CTR
GSE42865_WS
THIS_STUDY_CTR
THIS_STUDY_WS

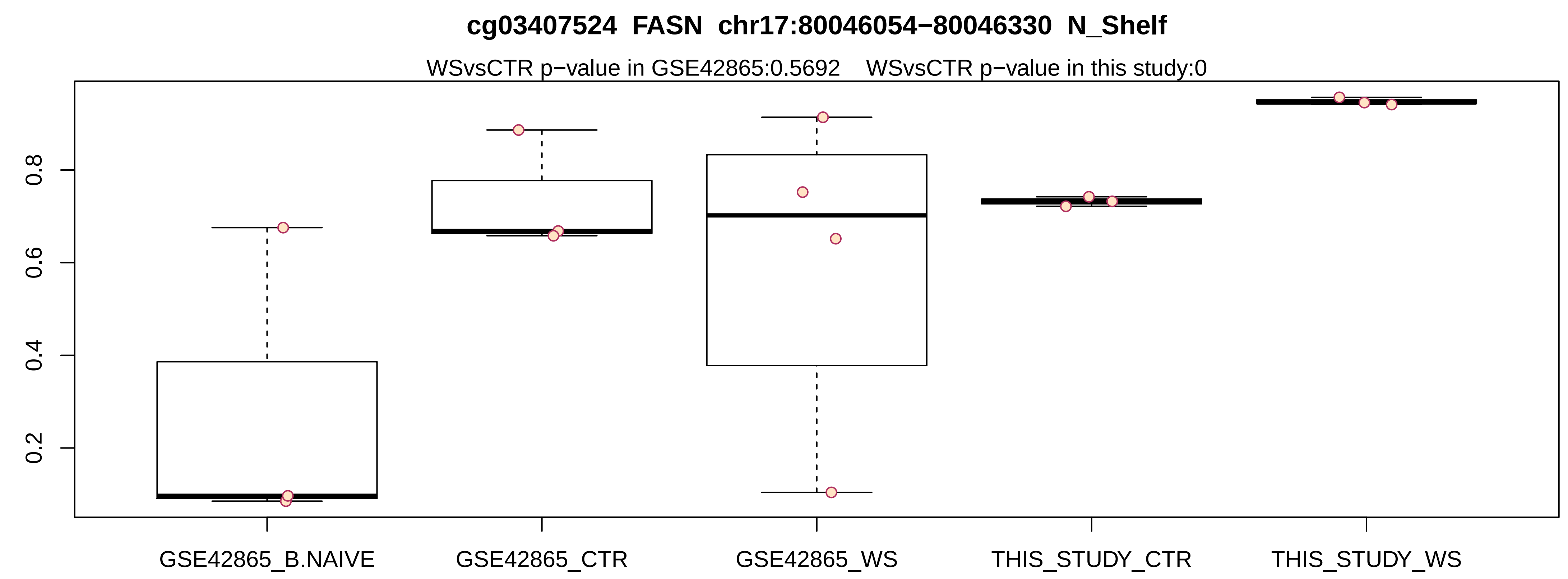
cg03407524 FASN chr17:80046054−80046330 N_Shelf
WSvsCTR p−value in GSE42865:0.5692 WSvsCTR p−value in this study:0
0.2
0.4
0.6
0.8
GSE42865_B.NAIVE
GSE42865_CTR
GSE42865_WS
THIS_STUDY_CTR
THIS_STUDY_WS

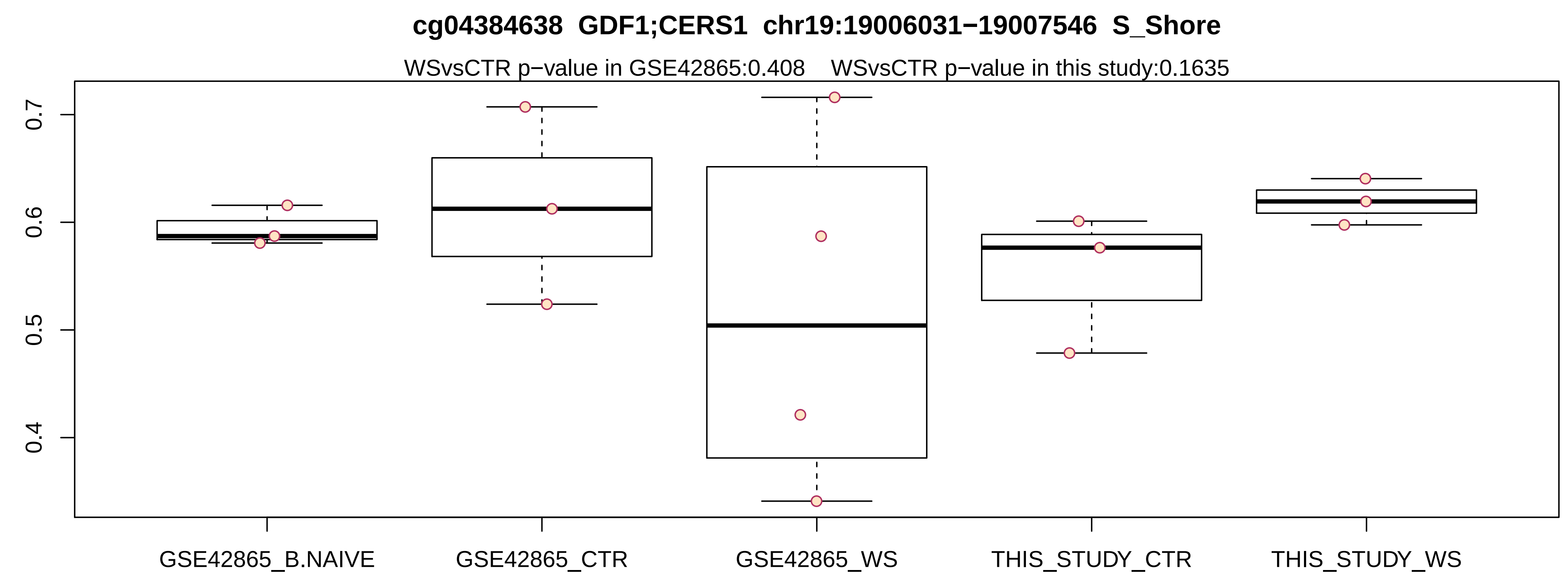
cg04384638 GDF1;CERS1 chr19:19006031−19007546 S_Shore
WSvsCTR p−value in GSE42865:0.408 WSvsCTR p−value in this study:0.1635
0.4
0.5
0.6
0.7
GSE42865_B.NAIVE
GSE42865_CTR
GSE42865_WS
THIS_STUDY_CTR
THIS_STUDY_WS

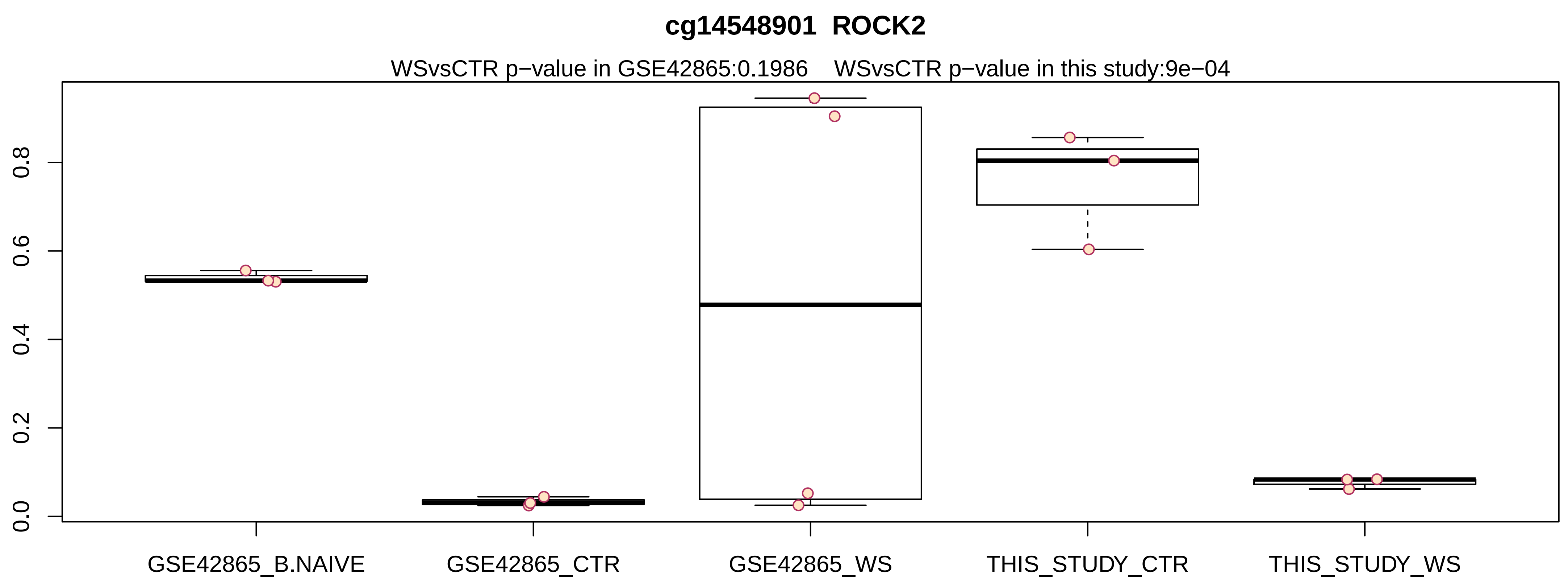
cg14548901 ROCK2
WSvsCTR p−value in GSE42865:0.1986 WSvsCTR p−value in this study:9e−04
0.0
0.2
0.4
0.6
0.8
GSE42865_B.NAIVE
GSE42865_CTR
GSE42865_WS
THIS_STUDY_CTR
THIS_STUDY_WS

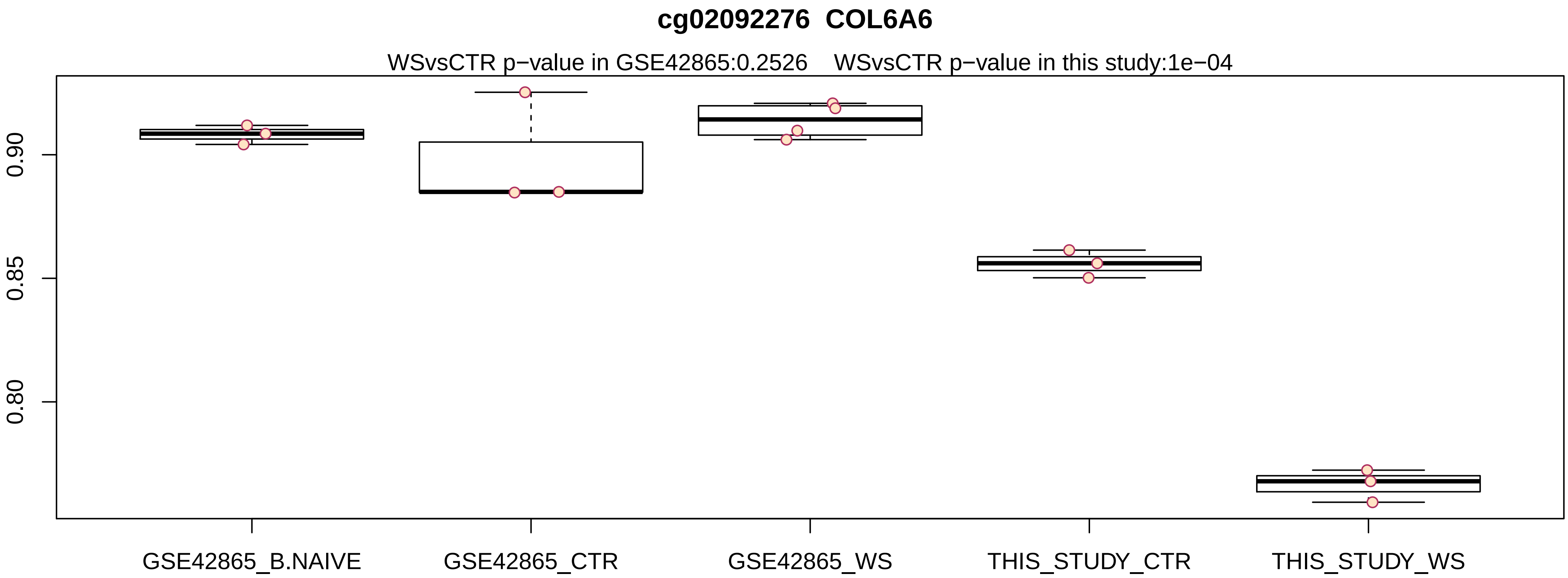

cg02092276 COL6A6
WSvsCTR p−value in GSE42865:0.2526 WSvsCTR p−value in this study:1e−04
0.80
0.85
0.90
GSE42865_B.NAIVE
GSE42865_CTR
GSE42865_WS
THIS_STUDY_CTR
THIS_STUDY_WS

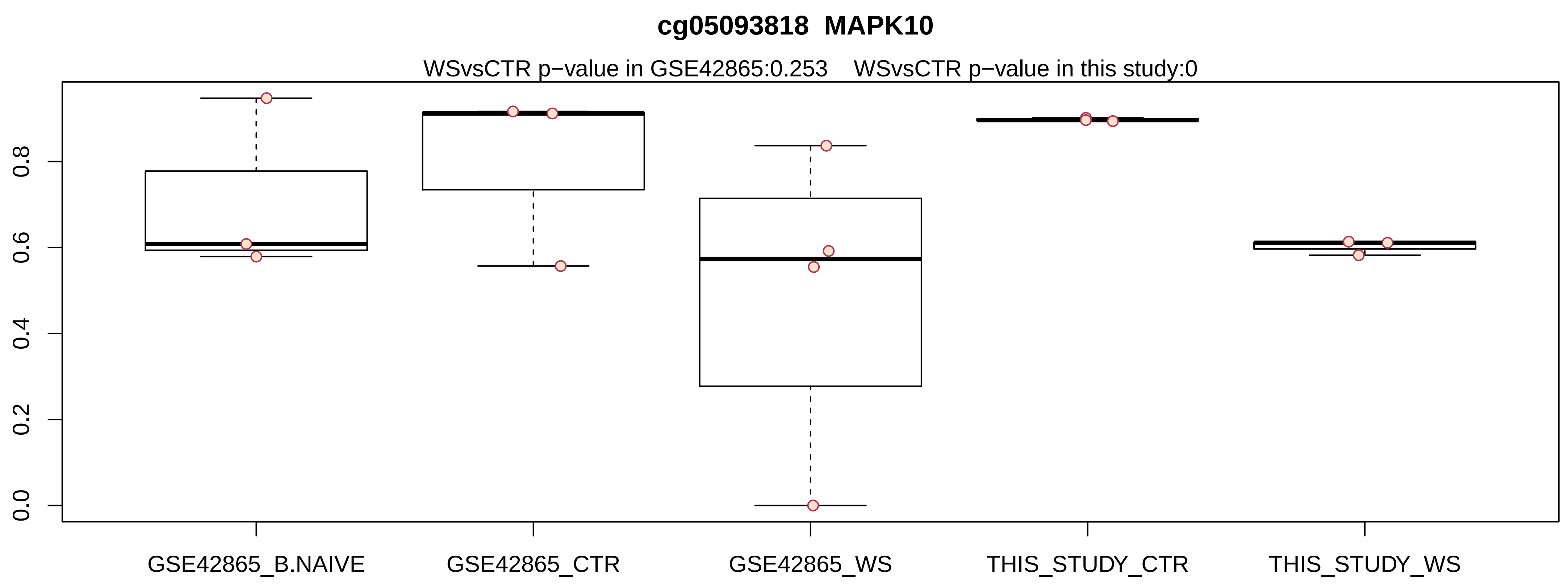
cg05093818 MAPK10
WSvsCTR p−value in GSE42865:0.253 WSvsCTR p−value in this study:0
0.0
0.2
0.4
0.6
0.8
GSE42865_B.NAIVE
GSE42865_CTR
GSE42865_WS
THIS_STUDY_CTR
THIS_STUDY_WS

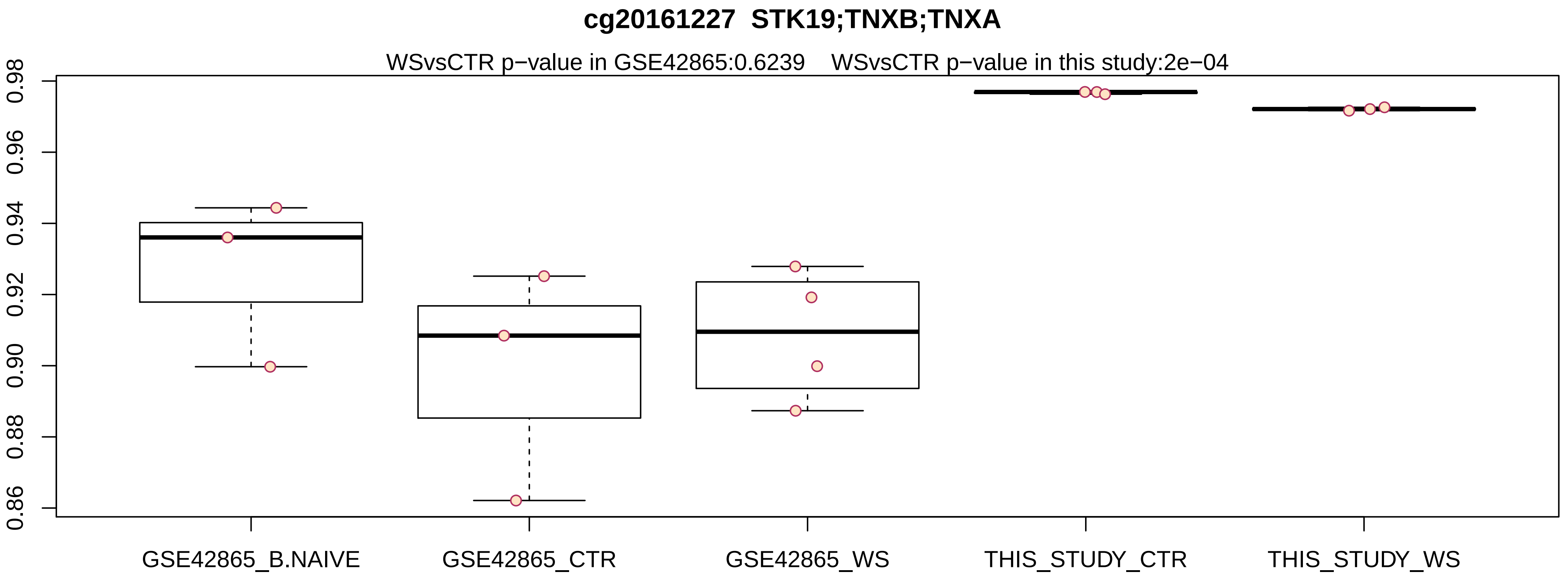
cg20161227 STK19;TNXB;TNXA
WSvsCTR p−value in GSE42865:0.6239 WSvsCTR p−value in this study:2e−04
0.86
0.88
0.90
0.92
0.94
0.96
0.98
GSE42865_B.NAIVE
GSE42865_CTR
GSE42865_WS
THIS_STUDY_CTR
THIS_STUDY_WS

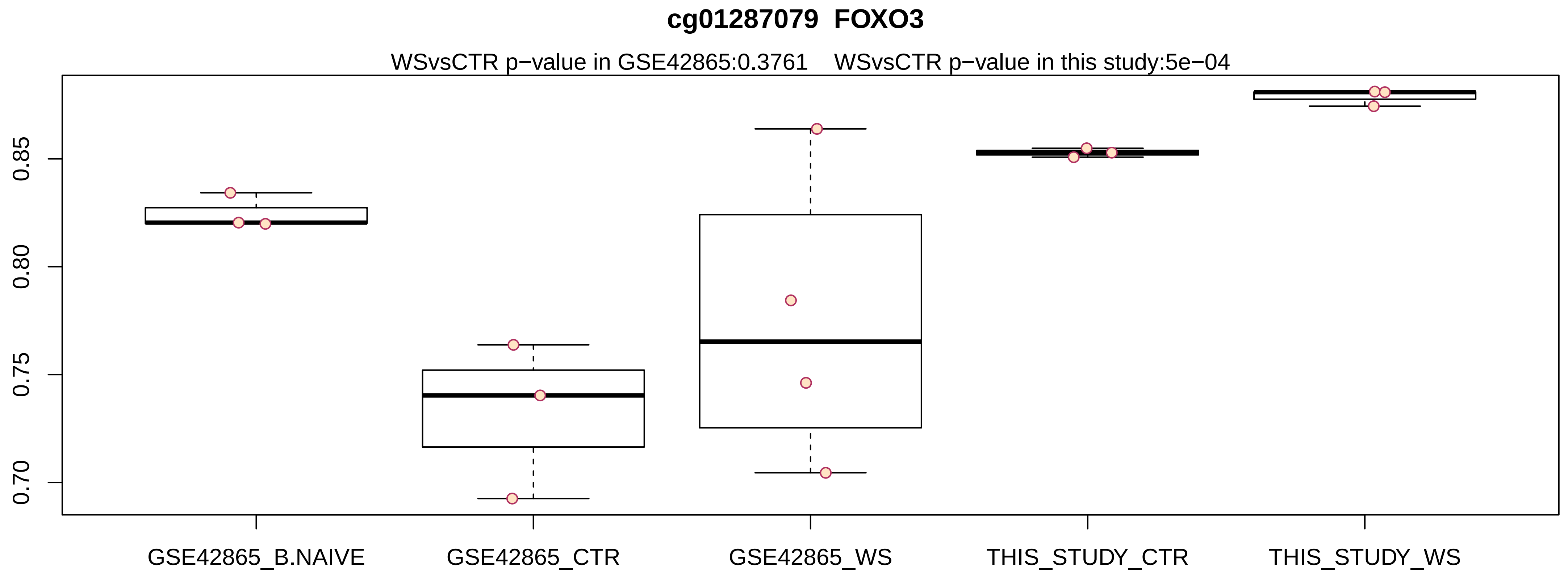

cg01287079 FOXO3
WSvsCTR p−value in GSE42865:0.3761 WSvsCTR p−value in this study:5e−04
0.70
0.75
0.80
0.85
GSE42865_B.NAIVE
GSE42865_CTR
GSE42865_WS
THIS_STUDY_CTR
THIS_STUDY_WS

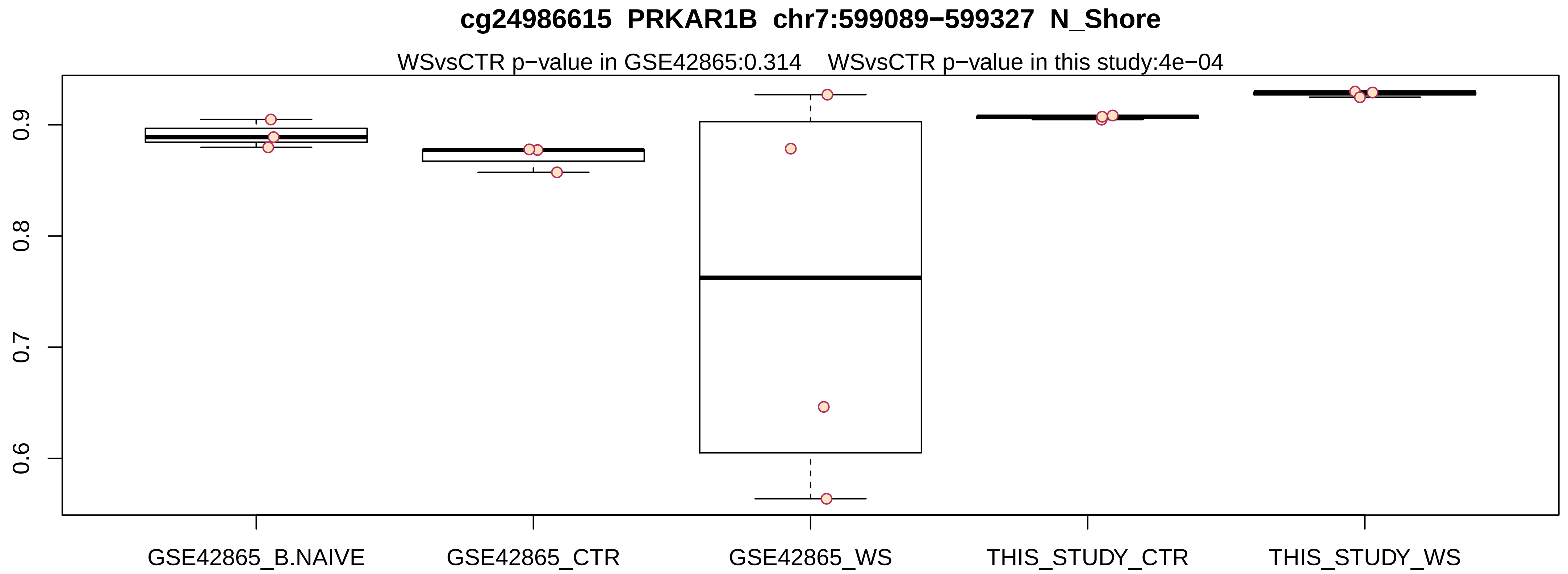
cg24986615 PRKAR1B chr7:599089−599327 N_Shore
WSvsCTR p−value in GSE42865:0.314 WSvsCTR p−value in this study:4e−04
0.6
0.7
0.8
0.9
GSE42865_B.NAIVE
GSE42865_CTR
GSE42865_WS
THIS_STUDY_CTR
THIS_STUDY_WS

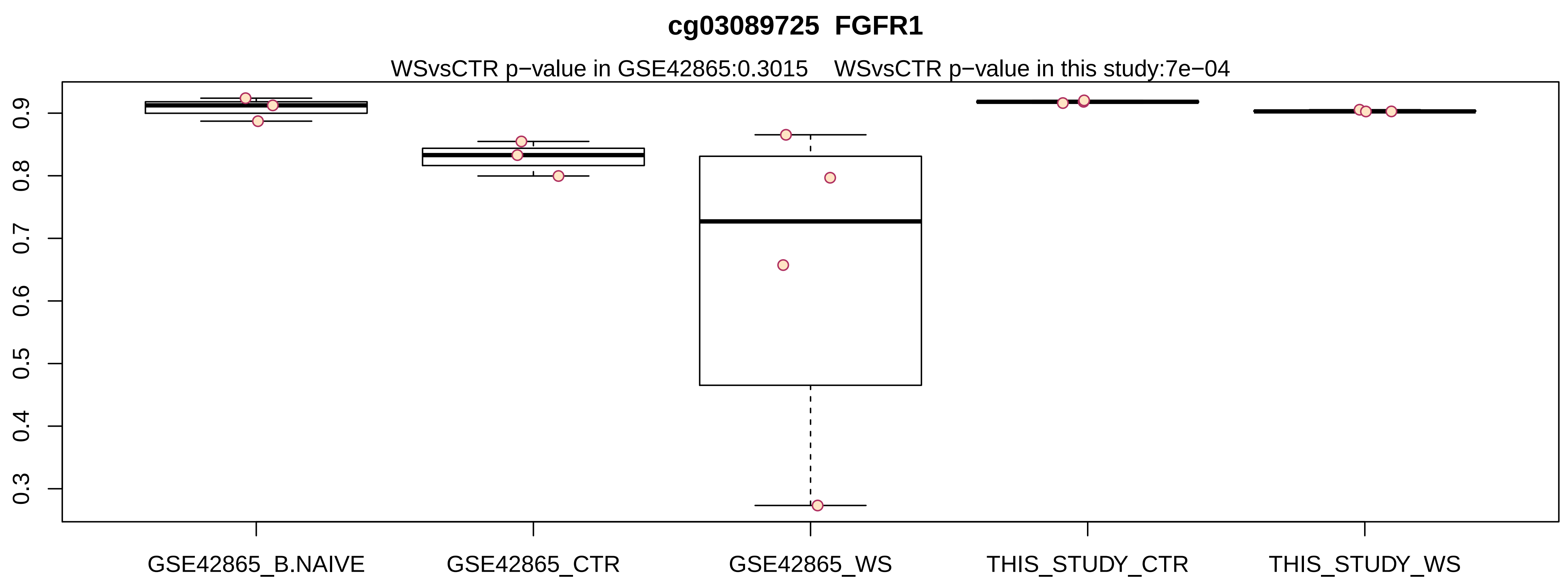
cg03089725 FGFR1
WSvsCTR p−value in GSE42865:0.3015 WSvsCTR p−value in this study:7e−04
0.3
0.4
0.5
0.6
0.7
0.8
0.9
GSE42865_B.NAIVE
GSE42865_CTR
GSE42865_WS
THIS_STUDY_CTR
THIS_STUDY_WS

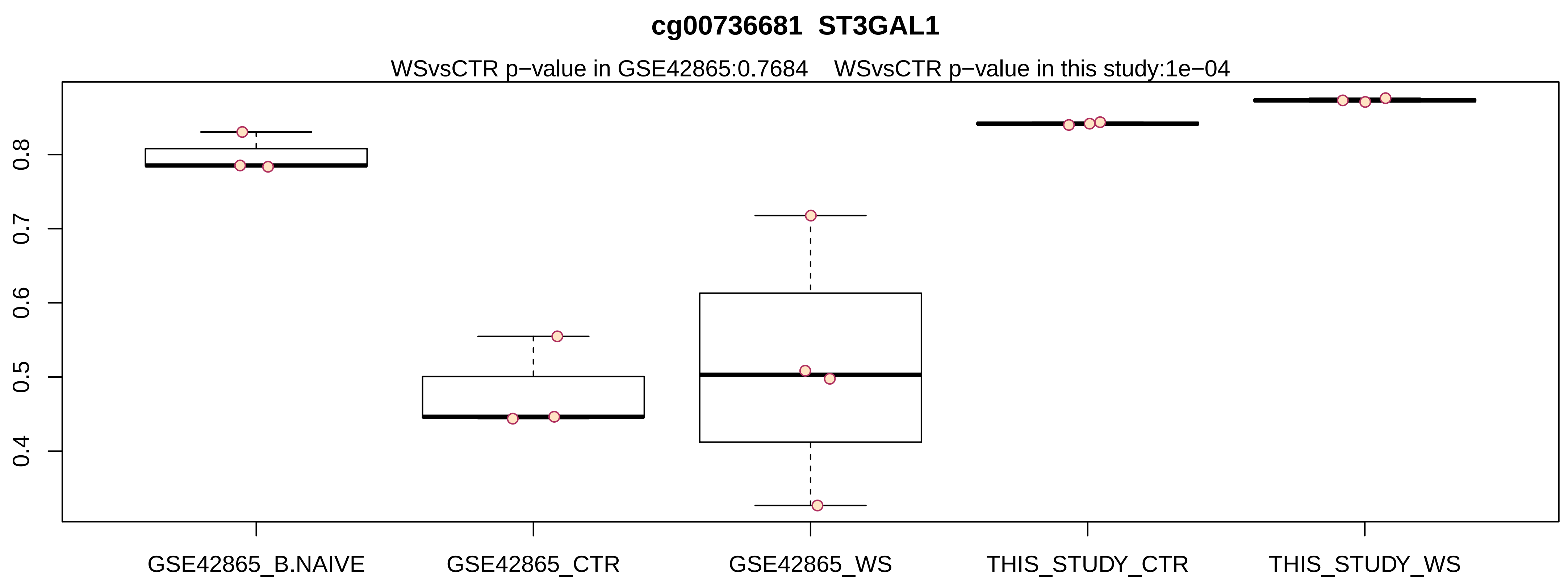
cg00736681 ST3GAL1
WSvsCTR p−value in GSE42865:0.7684 WSvsCTR p−value in this study:1e−04
0.4
0.5
0.6
0.7
0.8
GSE42865_B.NAIVE
GSE42865_CTR
GSE42865_WS
THIS_STUDY_CTR
THIS_STUDY_WS

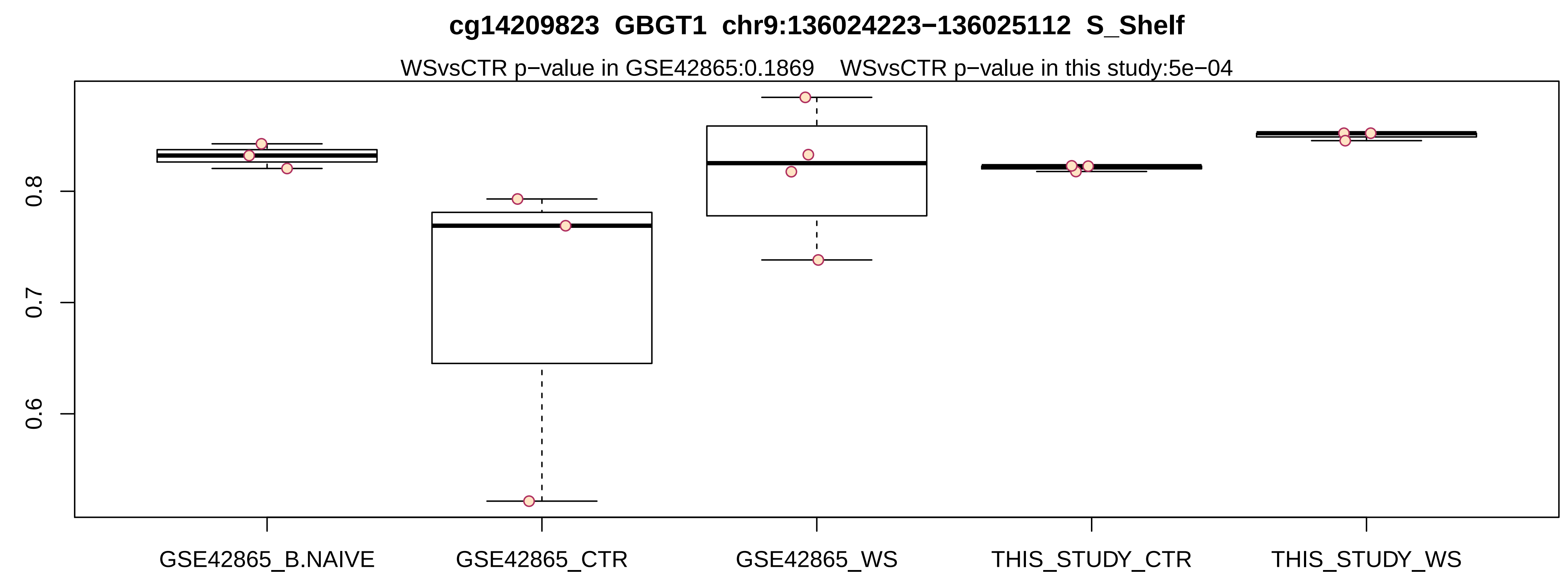

cg14209823 GBGT1 chr9:136024223−136025112 S_Shelf
WSvsCTR p−value in GSE42865:0.1869 WSvsCTR p−value in this study:5e−04
0.6
0.7
0.8
GSE42865_B.NAIVE
GSE42865_CTR
GSE42865_WS
THIS_STUDY_CTR
THIS_STUDY_WS
